# Suppression of spectral gap and flat bands on a cuprate superconductor side-surface

**DOI:** 10.1038/s41467-026-78020-y

**Published:** 2026-10-07

**Authors:** Gabriele Domaine, Mihir Date, Sydney K. Y. Dufresne, Natalie Lehmann, Daiyu Geng, Tohru Kurosawa, Amit Kumar, Jiaju Wang, Tianlun Yu, Chien-Ching Chang, Swosti P. Sarangi, Ding Pei, Yiran Liu, Julia Küspert, Shigemi Terakawa, Markel Pardo Almanza, Jiabao Yang, Izabela Biało, Matthew D. Watson, Timur K. Kim, Stephen M. Hayden, Kritika Singh, Banabir Pal, Matteo Minola, Johan Chang, Naoki Momono, Migaku Oda, Stuart S. P. Parkin, Andreas P. Schnyder, Niels B. M. Schröter

**Affiliations:** 1 Max Planck Institut für Mikrostrukturphysik, Halle, Germany; 2 Max-Planck-Institut für Festkörperforschung, Stuttgart, Germany; 3 Diamond Light Source Ltd, Harwell Science and Innovation Campus, Didcot, UK; 4 Department of Applied Physics, Hokkaido University, Sapporo, Japan; 5 Synchrotron SOLEIL, L’Orme des Merisiers, Saint-Aubin, France; 6 Physik-Institut, Universität Zürich, Zürich, Switzerland; 7 Department of Applied Physics, Graduate School of Engineering, The University of Osaka, Osaka, Japan; 8 Center for Future Innovation, Graduate School of Engineering, The University of Osaka, Osaka, Japan; 9 H. H. Wills Physics Laboratory, University of Bristol, Bristol, UK; 10 Institute of Materials Physics, Helmholtz-Zentrum Hereon, Geesthacht, Germany; 11 Faculty of Science and Engineering, Muroran Institute of Technology, Muroran, Japan; 12 Department of Physics, Hokkaido University, Sapporo, Japan; 13 Institut für Physik, Martin-Luther-Universität Halle-Wittenberg, Halle (Saale), Germany; 14 Halle-Berlin-Regensburg Cluster of Excellence CCE, Halle (Saale), Germany

**Keywords:** Topological matter, Superconducting properties and materials, Condensed-matter physics

## Abstract

Side surfaces of cuprate superconductors are expected to display a suppressed *d*-wave order parameter and zero-energy topological flat bands with a large density of states, making them susceptible to symmetry broken orders. Yet such surfaces have never been investigated with momentum-resolved, surface-sensitive probes, because high-temperature superconductors rarely cleave along them. Using focused-ion-beam milling to define a controlled breaking point, we expose pristine (110) side surfaces of overdoped La_2−*x*_Sr_*x*_CuO_4_ (*x* = 0.22) suitable for angle-resolved photoemission. We do not resolve an opening or closing of a superconducting spectral gap within our energy resolution ( ~ 4 meV), in agreement with theoretical predictions. Surprisingly, the expected zero-energy flat band peak is also suppressed, despite the high topographic quality of the surface. Self-consistent Bogoliubov–de Gennes calculations show that the measured geometric roughness of the cleaved surface is too weak to eliminate these modes. The calculations further demonstrate that bulk inhomogeneities characteristic of high-temperature superconductors, modelled as moderate Anderson-type disorder, can broaden the flat-band states beyond detectability. Our results provide the first momentum-resolved view of the electronic structure on a cuprate side surface and identify disorder as a plausible mechanism obscuring the spectroscopic signatures of the predicted flat-band states.

## Abstract (web summary)

The authors use focused-ion-beam milling to expose the (110) side surface of overdoped La_2x_Sr_x_CuO_4_, where a zero-energy topological flat band is expected. Angle-resolved photoemission experiments do not show the expected flat-band peak due to broadening of the flat band by Anderson-type disorder.

## Introduction

**Fig. 1:**
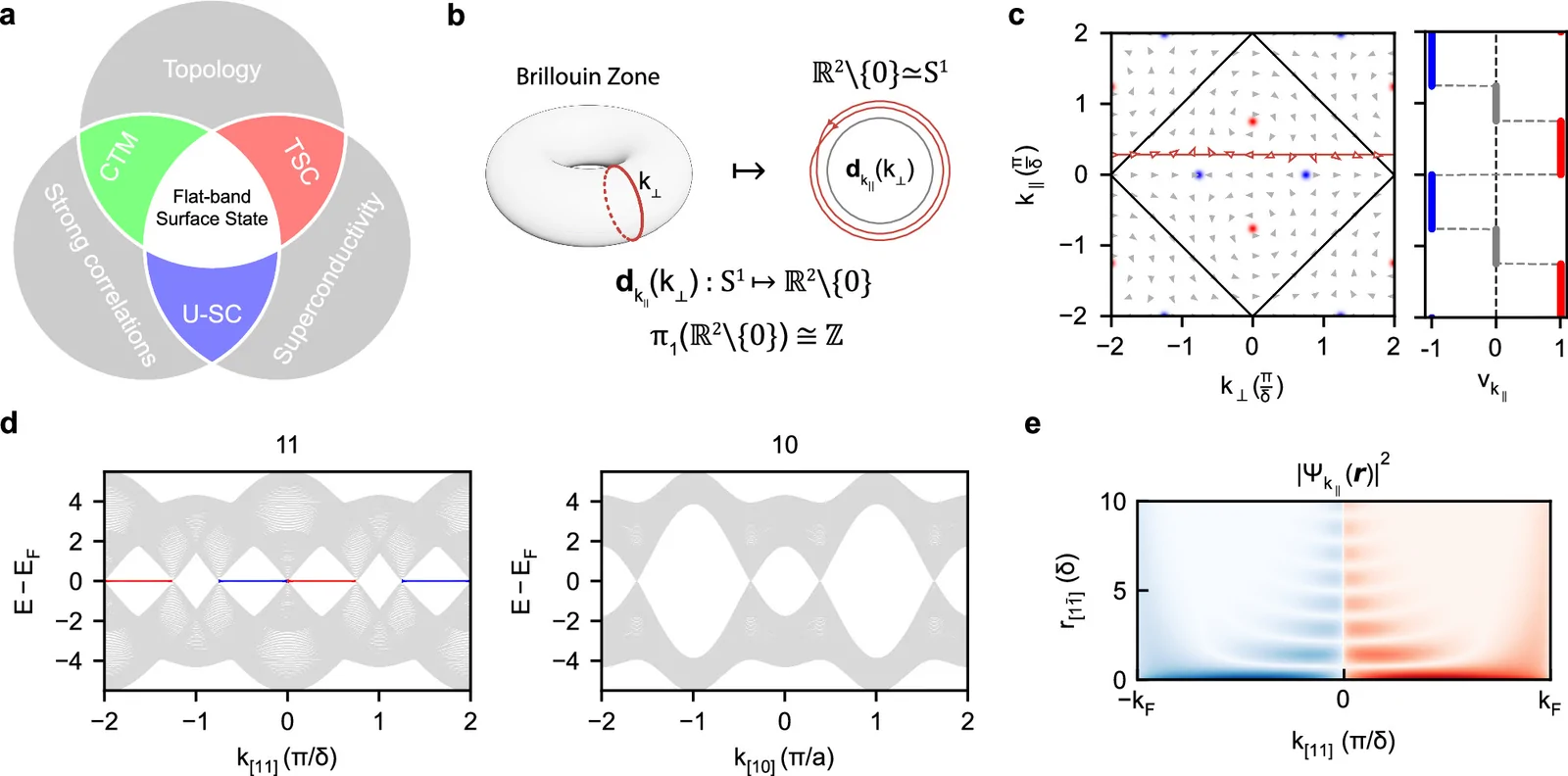
Edge states in clean ${d}_{{x}^{2}-{y}^{2}}$-wave superconductors. **a** Illustration of the realization of novel phenomena at the intersection of topology, strong correlations, and superconductivity, ranging from topological superconductivity (TSC), unconventional superconductivity (USC), and correlated topological matter (CTM). **b** Illustration of the mapping defined by the field ${{{\boldsymbol{d}}}}_{{k}_{\parallel }}$ from one-dimensional submanifolds of the Brillouin zone, which is homeomorphic to a 2-Torus, into the group ${{\mathbb{R}}}^{2}\setminus \{0\}$, which is homotopy equivalent to *S*^1^. **c** Illustration of the vector field ***d***_***k***_ for a ${d}_{{x}^{2}-{y}^{2}}$-wave superconductor in the first two Brillouin zones of a square lattice, so that *k*_⊥_ = *k*_[11]_ and ${k}_{\parallel }={k}_{[1\bar{1}]}$. The red horizontal line corresponds to a loop in momentum space as in the previous panel. The superconducting nodes of winding number +1 and −1 are shown in red and blue, respectively. The right inset shows the resulting winding numbers ${\nu }_{{k}_{\parallel }}$ computed along *k*_⊥_ and color-coded based on their sign, which also identifies their chirality. **d** Band structures for different edge orientations, with the two eigenvalues closest to the center of the spectrum highlighted in red and blue according to their chirality eigenvalues. The figure on the left corresponds to a [11] edge, in the same configuration as in (**c**), where the non-trivial values of the winding numbers lead to the formation of zero-energy flat bands connecting the projections of the superconducting nodes of opposite winding numbers. On the other hand, the figure on the right corresponds to a [10] edge, where the winding number is trivial for all *k*_∥_ = *k*_[10]_. In all the figures we defined $\delta=a\sqrt{(2)}$, with *a* the lattice constant of the square lattice. **e** Exponential decay of the edge states away from the edge (${r}_{[1\bar{1}]}=0$) and their delocalization in the bulk in correspondence with the projection of the superconducting nodes at *k*_[11]_ = − *k*_*F*_, 0, *k*_*F*_. The analytical expression of the edge-state wavefunction is derived in the Supplementary Section IA.

The intersection of superconductivity, topology, and strong correlations underlies many of the most unconventional quantum phases known today^1–16^. Within this broader landscape, the side surfaces of cuprate *d*-wave superconductors constitute a promising platform for studying the interplay of topology and strong correlations (Fig. 1a). Owing to the chiral symmetry of the Bogoliubov–de Gennes Hamiltonian, most of these surfaces host topological flat-band states pinned at the Fermi level^17–19^ (Fig. 1b–e). The extensive degeneracy of these states makes them highly susceptible to symmetry-breaking instabilities. This tendency is further amplified by the pair-breaking nature of these surfaces, which suppresses the local superconducting order parameter^20,21^ and thereby makes it energetically easier for competing orders to emerge. As a result, a variety of symmetry-broken phases have been predicted to emerge, including time-reversal symmetry-breaking states^20,22–24^, mixed-parity superconductivity^25^, phase-crystal states^26–30^, and magnetic order^30–32^.

Despite this potential and decades of experimental investigations of the cuprates, experimental evidence of order-parameter suppression due to pair-breaking edges remains limited to step-edges^33,34^, while direct identifications of topological side-surface states remain limited to zero-bias conductance peaks (ZBCPs) in tunneling measurements^35–40^. However, these signatures have often been subject to ambiguous interpretations. There have been numerous reports of ZBCPs obtained on surfaces that are not expected to support topological surface states^41–46^, whereas other studies have failed to observe a ZBCP altogether^47–49^. Several mechanisms have been proposed to reconcile these disparate findings, including the presence of inter-domain interfaces^50^, disorder-induced low-energy spectral weight in the quasiparticle DOS^51^, impurity-induced bound states near pair-breaking surfaces^52^, and the coexistence of multiple facets with different orientations^20,34^. However, the lack of momentum resolution in tunneling experiments fundamentally limits the ability to distinguish topological flat bands from topologically trivial zero-bias features. A surface-sensitive, momentum-resolved probe is therefore needed, as the surface states are confined within a coherence length from the surface and exhibit a characteristic momentum-space distribution not expected for disorder-induced in-gap states. In addition, momentum resolution is essential for accurately identifying and characterizing potential symmetry-broken phases.

However, such measurements have been hindered by the layered structure of the cuprate superconductors, which prevents clean side surfaces from being obtained by conventional cleavage. Recently, however, a novel sample-preparation technique for Angle-Resolved Photoemission Spectroscopy (ARPES) was demonstrated on the normal state of Sr_2_RuO_4_^53^. In this approach, focused-ion-beam (FIB) milling is used to mill micro-notches into the sample in order to enforce a desired cleavage plane perpendicular to the layers. Here, we apply this approach for the first time to a cuprate high-temperature superconductor. Using FIB milling to define a cleaving plane, we expose pristine (110) side surfaces of overdoped La_2−*x*_Sr_*x*_CuO_4_ (LSCO) with *x* = 0.22 suitable for ARPES, enabling the first momentum-resolved measurements on a cuprate side surface in the superconducting state. So far, ARPES measurements have only investigated the (001) top surface of this compound and found a superconducting gap of 15–25 meV^54^.

Our measurements reveal a collapse of the spectral gap on the (110) surface, but surprisingly, no peak associated with the predicted flat-band surface states is observed in the raw spectra, despite high instrumental energy and momentum resolution. Fully momentum-resolved, self-consistent Bogoliubov–de Gennes tight-binding calculations indicate that the experimentally measured surface roughness, within the experimental resolution, is insufficient to account for this absence. Moreover, motivated by the characteristic bulk inhomogeneity observed in high-temperature cuprate superconductors^55–59^, we introduce moderate Anderson disorder with a magnitude comparable to the superconducting gap into our model and show that such disorder can strongly suppress the flat-band surface states. Our results show that side-surface ARPES enabled by FIB cleaving provides momentum-resolved access to the electronic structure of previously inaccessible cuprate surfaces, and reveal the importance of both geometric and Anderson-type disorder in the detection of these surface states, laying groundwork for future studies of correlated topological superconductivity.

## Results and discussions

### Expected momentum structure of flat band surface states

The expected momentum structure of the topological side-surface states originates from a topological ${\mathbb{Z}}$ winding number that the superconducting quasiparticles carry near the nodes of the d-wave order parameter, which is protected by the chiral symmetry of the cuprate superconductors (Fig. 1b, see also the Supplementary Section IA). Because the spectrum is gapless, this invariant can only be defined on one-dimensional loops that avoid the nodes. A natural choice is therefore to consider loops indexed by the momentum parallel to the edge, so that one can identify a topological sector—where the winding is non-zero and requires the existence of zero-energy edge states^18,19^—and a trivial sector, where the winding vanishes. For a d-wave order parameter, the topological sector is bounded by the projection of two superconducting nodes with opposite winding number (Fig. 1c), so that in momentum space the topological states form flat bands that connect the projected superconducting nodes (Fig. 1d, e). This leads to a unique alternating momentum structure where the flat bands appear in some but not all of the projected bulk band gaps, becoming the largest for the [11] edge, which motivated us to search for them with ARPES.

### Surface preparation and characterization

**Fig. 2:**
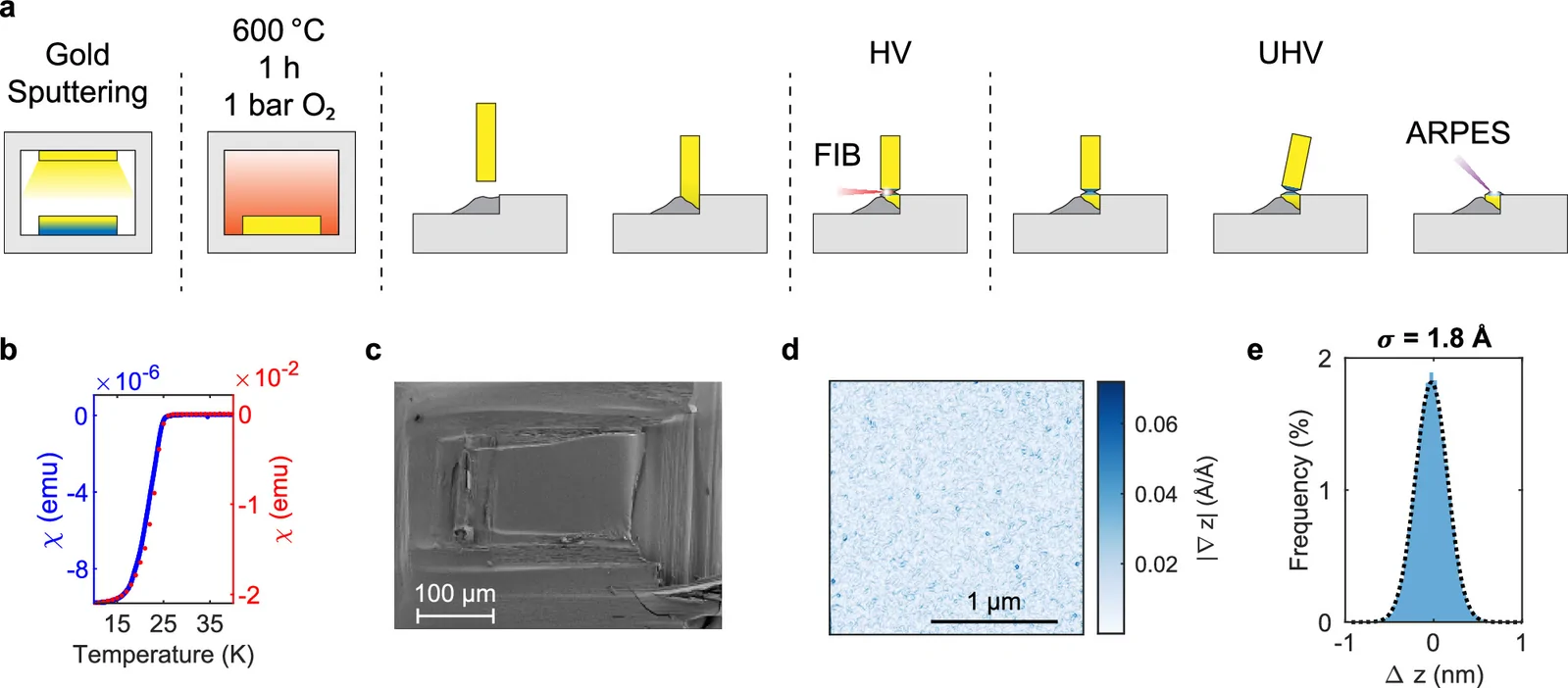
Sample preparation. **a** Illustration showing the different stages of the sample preparation. **b** Temperature dependence of the magnetic susceptibility measured by SQUID magnetometry for a piece of the original rod (red) and of a small part of the milled region of the pillar (blue). Measurements were performed under zero-field-cooled conditions with an applied magnetic field of 5 Oe (red) and 2 Oe (blue), showing no change in the critical temperature during sample preparation. **c** Scanning electron microscope image of the sample after the mechanical cleave. **d** Topography gradient map of the cleaved surface obtained by atomic force microscope and **e** its corresponding height distribution showing a roughness of only 1.8 Å (for comparison, the in-plane lattice constant of LSCO is 3.78 Å). The roughness is defined as the root-mean-square deviation of the height profile.

A schematic of the sample preparation is shown in Fig. 2a. The samples are oriented with Laue and then gold-coated and annealed to make a good electrical contact. The samples are then glued, a micro-notch is carved with FIB, and the samples are subsequently cleaved in the ARPES chamber. This process does not affect the bulk superconductivity of the samples, as can be seen from measurements of the magnetic susceptibility before and after the milling process (Fig. 2b). When measuring the magnetic susceptibility after the milling, only a thin slab at the top of the cleaved crystal was used in order to maximize the fraction of milled sample being measured. Figure 2c–e shows a scanning electron microscope (SEM) image of a cleaved surface after focused ion beam milling, and the atomic force microscope (AFM) characterization of the surface roughness. The roughness of the cleaved surface, defined as the root-mean-square deviation of the height profile, was determined to be approximately 1.8 Å, which corresponds to about one half of the in-plane lattice constant of LSCO^60^, or equivalently, to roughly one third of the unit cell dimension along the [110] crystallographic direction. This roughness is comparable to reported values for YBa_2_Cu_3_O_7−*δ*_ epitaxial thin films where a uniform ZBCP has been observed with scanning tunneling spectroscopy^36^.

### Fermi surface characterization

**Fig. 3:**
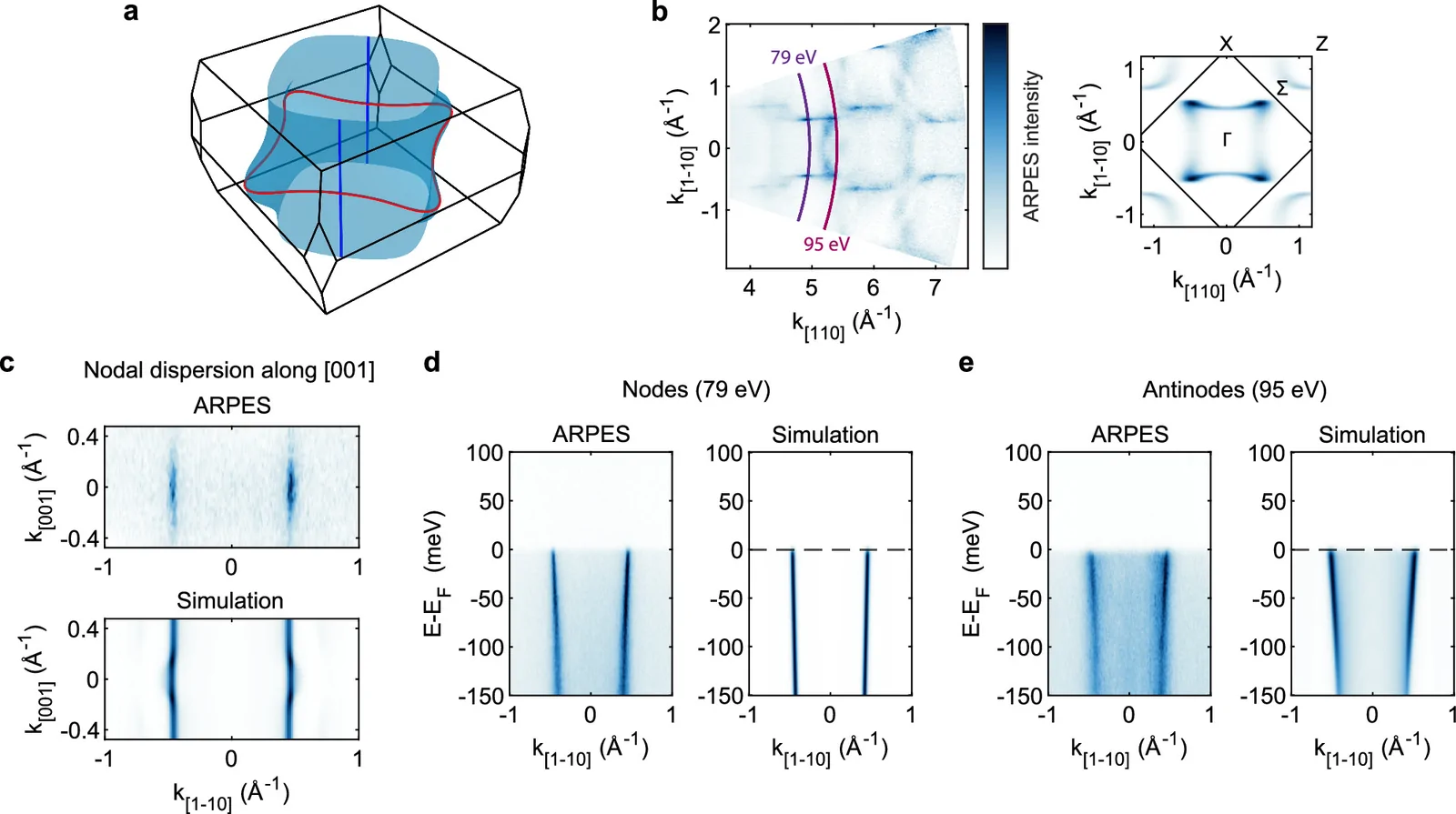
ARPES spectra of a (110) surface of LSCO. **a** Calculated three-dimensional Fermi surface of LSCO showing the contours at *k*_*z*_ = 0 (red) as in (**b**) and the nodal cut (blue) as in (**c**). **b** Varying the photon energy on a (110) surface yields a cut of the well-known (001) Fermi surface of LSCO shown in the left plot. The right plot shows the calculated (001) Fermi surface at the Γ*X*Σ plane, for comparison. **c** On the other hand, varying the analyzer deflector angle allows to directly obtain the dispersion of the bands along the [001] direction. Here the photon energy was 79 eV, corresponding to the nodal cut shown also in the next panel. **d** Dispersion of the bands at the superconducting nodes taken with a photon energy of 79 eV, and **e** at the antinodes taken with a photon energy of 95 eV. Notice that due to *k*_*z*_ broadening, the small dispersion of the bands at the antinodes is not recognizable, as the bands become smeared out along the [110] direction, along which they have a large dispersion. This results in the strong intensity observed between the two visible bands. All the calculations include a *k*_*z*_ broadening of Δ*k*_[110]_ = 0.2 Å^−1^, based on the typical inelastic mean-free path of photoelectrons with energies in the Vacuum Ultraviolet (VUV) region, which is about 5 Å. All measurements were performed at 6 K.

The quality of the cleaved surface enables us to acquire high-resolution ARPES spectra, which in turn allow for a complete mapping of the three-dimensional Brillouin zone of LSCO (Fig. 3). Due to the orientation of the cleaved side surface, varying the incident photon energy permits momentum scans along the [110] crystallographic direction (i.e. along *k*_*z*_ perpendicular to the cleaved side surface), thereby recovering the well-known Fermi surface in the (001) plane of LSCO (Fig. 3b). Conversely, a simple deflection scan of the electron analyzer provides direct access to the electronic structure along the plane spanned by the [001] and [1$\bar{1}$0] crystallographic directions (Fig. 3c). Finally, by selecting appropriate photon energies, we can resolve the electronic dispersions at both the nodal and antinodal regions of the Brillouin zone (Fig. 3d, e). It should be noted that, due to *k*_*z*_ broadening, the weak dispersion of the bands at the antinodal region cannot be resolved. This effect is also evident in the tight-binding calculations (see the “Methods” section “Three-dimensional Fermi surface”), where the *k*_*z*_ broadening was explicitly included. The inelastic mean free path of the photoemitted electrons was evaluated using the Tanuma-Powell-Penn (TPP-2M) formula^61^ (see the “Methods” section “Tanuma-Powell-Penn formula”) and used to determine the magnitude of the *k*_*z*_ broadening, yielding a value on the order of 0.2 Å^−1^. With this amount of broadening, the nodal and antinodal regions remain distinguishable. Such broadening can reduce the contrast of superconducting-gap features, but it would still allow for the identification of a superconducting gap if there were any.

The tight-binding contour shown for comparison in Fig. 3b was generated from the one-band LSCO parametrization of ref. ^62^. In this comparison, the dressed hopping parameters were kept fixed to the literature values, and only the chemical potential was rigidly shifted so as to reproduce the value of *k*_*F*_ measured experimentally in the nodal cut. This calculation is therefore intended as a visual guide to the experimental momentum-space structure, and small discrepancies between the measured intensity and this idealized contour are expected due to the simplified one-band nature of the model, photoemission matrix-element effects, residual distortions of the experimental momentum conversion, and the finite *k*_*z*_ broadening inherent to the side-surface photon-energy map. The effective doping of the measured surface was instead estimated from the experimentally extracted Fermi-surface area using the Luttinger-count procedure described in Supplementary Section IF, giving a surface hole doping of approximately *p* ≃ 0.19. This value is compatible with the nominal bulk doping *p* = 0.22 when accounting for the small known discrepancies between Luttinger-count estimates and nominal Sr concentration reported also in conventional LSCO(001) ARPES measurements^63^, for which we also note that LEED measurements did not find evidence for a surface reconstruction^64^. Thus, within the accuracy of the analysis, the measured side surface remains in the overdoped superconducting regime and does not indicate a significant surface reconstruction of the type reported in some other cuprates^65^.

### Broadening of the surface states and gap frustration by pair-breaking

**Fig. 4:**
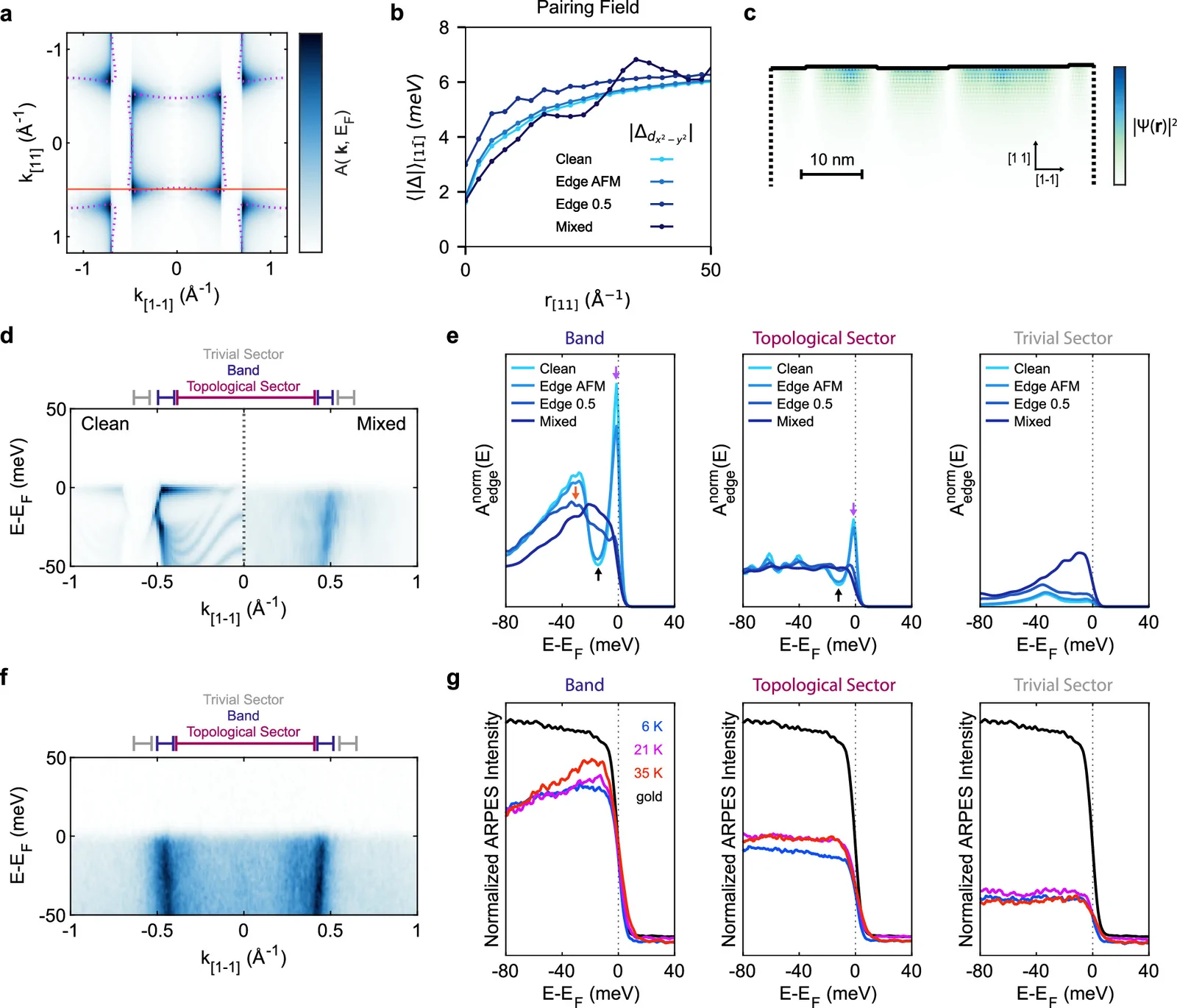
Boundary effects at a (110) surface of a ${d}_{{x}^{2}-{y}^{2}}$-wave superconductor. **a** Simulated spectral function of the [11] edge at the Fermi level. **b** ${d}_{{x}^{2}-{y}^{2}}$ pairing amplitude versus distance from the [11] edge for a clean system (C), AFM-derived geometric edge roughness (GE_AFM_), probabilistic roughness (GE_0.5_), and GE_AFM_ combined with bulk Anderson disorder (BA). The BA disorder is Gaussian with a standard deviation of 100 meV. **c** Real-space expectation value of edge-state eigenstates for AFM-derived geometric edge roughness. States are selected by their eigenvalues and participation ratios. Dotted lines indicate periodic boundary conditions. **d** Simulated antinodal spectral functions for C (left) and GE_AFM_ + BA disorder (right), extracted along the red line in (**a**). **e** Simulated antinodal EDCs for different disorder realizations and *k*[11]; purple, black, and orange arrows mark the surface-state peak, DOS dip, and coherence peak, respectively. **f** ARPES spectra measured with 95 eV photons and at 6 K (see Fig. 3b). **g** Antinodal EDCs (*hν* = 95 eV) normalized by total intensity at different temperatures, compared with the gold DOS obtained at 6 K. The integration ranges are indicated by the colored lines above (**d**, **f**) (see also the Supplementary Section IG). Reported values are mean intensities within these ranges, normalized to the total intensity. For the ARPES data, a background subtraction following ref. ^90^ was applied prior to the EDC extraction. All calculations are performed at 0 K.

Although surface states are typically regarded as effectively two-dimensional states localized at the boundary of the system, this picture is modified in the limit of a small gap, as occurs in the case of topological superconductors. For comparison, typical chiral topological semimetals exhibit projected gaps on the order of 0.1–1 eV^66,67^ leading to strong confinement of Fermi-arc surface states at the surface, whereas the magnitude of the superconducting gap of LSCO at the antinodes is typically 10–20 meV^54,68^, consistent with our (001)-surface reference measurement on a comparably prepared sample (Supplementary Section IE). In this regime, the surface states are no longer strongly confined to the boundary but instead acquire a finite degree of bulk character, and the spectral weight develops a dependence on the *k*_*z*_ momentum component perpendicular to the surface (Fig. 4a). This behavior can also be understood by considering the limit where the bulk superconducting gap approaches zero. In this case, the system should host only bulk states, and the spectral weight associated with zero-energy modes should evolve continuously into the spectral weight of the normal-state Fermi surface bounded by the projection of the superconducting nodes (see the Supplementary Section IB). From an experimental standpoint, one must then select regions of the Brillouin zone corresponding to *k*_*z*_ in which the spectral weight of the bulk superconducting state deviates the most from the spectral weight of the surface state, while ensuring that the surface-state spectral function retains sufficient intensity.

For ${d}_{{x}^{2}-{y}^{2}}$-wave superconductors, this condition should be satisfied near the antinodes (*hν* = 95 eV, see Fig. 3e), where the opening of the superconducting gap would be expected to deplete the bulk states at the Fermi level. However, this gap actually gets filled in the near surface region, since a ${d}_{{x}^{2}-{y}^{2}}$-wave order parameter is suppressed at a (110) surface due to the phase shift acquired by reflected quasiparticles, which in turn frustrates the order parameter over a length scale comparable to the superconducting coherence length (Fig. 4b, c and refs. ^20,21,33^), which is on the order of a few nanometers in the cuprates^69–71^, larger than the typical probing depth of ARPES (see the “Methods” section “Tanuma-Powell-Penn formula”). Therefore, one in general does not expect the superconducting gap to be observable at a (110) surface, which poses a potential complication for separating the edge and bulk spectral weight. Remarkably, despite the suppression of the superconducting gap, the simulated spectra corresponding to a *k*_*z*_ value at the antinodes on the (110) surface still show a discernible dip and peak in the spectra (Fig. 4d, black arrow in Fig. 4e). This feature may serve as a characteristic signature for identifying the surface states irrespective of the gap suppression. Indeed, this local suppression is not prominently reflected in the edge spectrum, because the low-energy edge spectral function is dominated by zero-energy Andreev bound states. Consistently, our calculations show that including a self-consistent suppression of the order parameter near the edge produces negligible changes in the low-energy edge spectral function (Supplementary Section IJ).

Nevertheless, although bulk superconductivity has been confirmed in our samples by magnetic susceptibility measurements (Fig. 2b), and the surface doping level is consistent with the bulk value (see Supplementary Section IF), our spectra at *k*_*z*_ values corresponding to the antinodes show no clear dip–peak structure at the Fermi level within the experimental energy resolution of 3–4 meV (see Fig. 4g and the corresponding integration ranges in Supplementary Section IG). Even accounting for the uncertainty in the estimated surface doping, moderate deviations from the expected doping would only negligibly shift the nodal coordinates^63^, and thus the momentum extent of the topological flat band, without substantially modifying the spectral function. If the doping change were large enough to generate a gapless metallic layer proximity-coupled to the underlying *d*-wave superconducting state, one would not expect a trivial broadening of the flat band. Instead, this scenario would produce discrete quantum-well states (Supplementary Section IK), for which we find no evidence in the experimental data. Finally, we do not resolve any temperature-dependent spectral feature that could be associated with the opening or closing of a superconducting gap, which is expected to be approximately 20 meV at our doping level^54^. Although this observation does not by itself prove order-parameter suppression, given its weak influence on the edge spectral function, it is nevertheless consistent with expectations for a pair-breaking boundary dominated by Andreev physics, as discussed above. Notice that the small temperature dependence of the intensity observed in both the band and Topological sector Energy Distribution Curves (EDCs) was not reproduced in another sample that was measured at different temperatures (Supplementary Section IH); thus, we attribute this to extrinsic effects such as minor thermal drift or variations in surface conditions.

To account for these observations, we incorporated geometric edge disorder in our simulations, implemented by removing sites along the edge using an arbitrary section of the experimentally measured surface topography (Fig. 2e) obtained by AFM (“Methods” “Two-dimensional slab”). Owing to the single-orbital nature of our model, the effective spatial resolution along the [11] direction is limited to $\frac{a\sqrt{2}}{2}$, where *a* is the lattice constant. The resulting simulated spectra still exhibit the dip and surface-state peak (Fig. 4e), since the disordered edges still exhibit sufficiently long clean segments extending over multiple coherence lengths (Fig. 4c, top panel). To achieve a substantial broadening of the surface states, the surface height must vary with very high in-plane spatial frequency—that is, the roughness must entirely eliminate any contiguous flat regions down to the size of a single unit cell. Such lateral resolution is beyond the capabilities of conventional AFM setups, which are typically limited to a few nanometers^72–74^. Moreover, the small sample size (on the order of a few hundred micrometers) makes it difficult to reliably engage with a scanning tunneling microscopy (STM) tip. Nevertheless, our results establish an upper bound on the disorder autocorrelation length and show that, for the disorder resolvable by our AFM measurement, the surface states remain intact. Finally, disorder with sufficiently high in-plane spatial frequency would be expected to partially lift the suppression of the gap at the edge (Fig. 4b and orange arrow in Fig. 4e). Although the emergence of a fully developed gap would likely remain unobservable under these conditions, one may nevertheless expect subtle modifications of the low-energy spectral features in agreement with the closure of the gap upon varying the temperature across the critical temperature. However, we do not observe such signatures.

Beyond geometric disorder at the surface, it is well established that most high-temperature cuprate superconductors exhibit substantial intrinsic disorder, arising primarily from chemical doping and oxygen vacancies^51,55–58^. While more realistic descriptions usually account for this disorder through a distribution of point-like and extended impurity potentials with characteristic strengths ranging from several tens to several hundreds of meV^75–77^, the exact microscopic details are not required for developing qualitative intuition regarding its impact on the surface states. Accordingly, consistent with the deliberately minimal character of the present model, we approximate the effects of such intrinsic bulk inhomogeneity by an Anderson-type random onsite mass term applied throughout the simulated slab. This choice reflects the fact that the relevant disorder sources in cuprates, such as dopant disorder and oxygen vacancies, are not confined to the outermost surface layer. Nevertheless, the broadening of the flat-band surface state is governed mainly by the component of this disorder that overlaps with the surface-state wave functions, which are localized near the boundary over a length scale set by the superconducting coherence length. Importantly, although disorder preserving chiral symmetry does not mix states with the same chirality eigenvalues, scattering between different chirality sectors is still allowed (Supplementary Section IC). The efficiency of this broadening is controlled not only by the overall disorder strength, but also by the disorder correlation length and by the Fourier weight of the disorder potential at the momentum transfer connecting opposite chirality sectors. Since eigenstates of opposite chirality reside at opposite momenta, this inter-sector coupling necessarily involves large-momentum-transfer scattering processes. Uncorrelated onsite disorder has a broad Fourier spectrum and therefore provides an efficient limiting case for testing the sensitivity of the flat-band peak to short-length-scale scattering. Other short-range disorder sources, such as pointlike impurities, strongly localized defect potentials, or high-frequency surface impurity potentials, are expected to have a qualitatively similar effect if they generate comparable large-momentum-transfer scattering. By contrast, spatially smooth or extended disorder potentials would have reduced large-momentum-transfer components and would therefore be expected to broaden the flat-band peak less efficiently than the uncorrelated onsite disorder used here. For the disorder realization shown in Fig. 4d, e, we find that the inclusion of a random mass term at each site, with a magnitude drawn from a Gaussian distribution with a standard deviation of 100 meV, lifts the degeneracy of the flat band and strongly suppresses the associated peak. A comparison of different disorder strengths shows that the flat-band peak remains partially visible for weaker disorder; see the Supplementary Section IM where additional disorder realizations are also compared. We further observe a relative increase in spectral weight in the trivial sector near the Fermi level (Fig. 4e); however, this feature may be experimentally challenging to resolve due to background intensity and the possible contribution of geometric disorder with higher in-plane spatial frequencies. The simulated cut at the antinodes with both edge roughness and onsite disorder is shown in the right half of Fig. 4d, highlighting the visual agreement with the experimental results (Fig. 4f). Notice that this amount of disorder is not inconsistent with the observation of a superconducting gap at surfaces where there is no surface state, such as the (001) surface, which is typically measured by ARPES experiments (Supplementary Section II).

### Discussion

With this interpretation, the ZBCPs observed in STM measurements^33,34,36^ can plausibly be attributed to the differences in spatial resolution compared to ARPES, which is limited by the beam spot size (approximately 50 μm in our measurements), so that one cannot isolate nanoscale regions with lower disorder. Indeed, as shown in Fig. 4c, the edge roughness already produces an inhomogeneous spatial distribution of the edge-state wavefunction. Upon introducing Anderson disorder, this spatial fragmentation is further enhanced, such that the surface states may reside only on isolated segments of the boundary whose contribution to the photoemission intensity is strongly diluted when averaging over the full beam spot area. This effect has also been reported in STM measurements at step edges^33^, where the relative prominence of the zero-bias peak was found to vary substantially between different positions along the edge. Crucially, the inclusion of on-site disorder enhances this spatial inhomogeneity, further complicating the isolation of the edge state by ARPES. Even when a relatively uniform ZBCP was observed in STM measurements^36^, the reported spatial range only covers about 16 nm, which is two orders of magnitude smaller than our spatial resolution.

On the other hand, tunnel junction measurements, which also probe a larger area but do not require an atomically smooth surface, still observe a zero-bias peak. One possible explanation is that dilute disorder can displace the surface states away from the topmost layer^78^, reducing their visibility in surface-sensitive probes like VUV ARPES while allowing them to contribute to the tunneling signal. An additional explanation is that at least some of the ZBCPs observed in tunneling measurements could be due to interface states arising between domains with different orientations^50^. In the light of this complicated picture, one must notice that disorder can manifest in various forms which can have markedly different effects on the surface states depending on their symmetries as well as their spatial distribution. For instance, one could consider onsite disorder, such as chemical potential disorder or mass disorder (used in our calculations), as well as disorder in the hopping terms. These types of disorder can furthermore be either localized near the edge or extended throughout the bulk, and their distribution can, for instance, be Gaussian or dilute^78^, and exhibit varying degrees of spatial correlation. Determining which type of disorder affects a certain measurement is thus evidently challenging in an experimental setting. Adsorbates provide a possible source of surface disorder. However, their spectroscopic signatures evolve gradually over several hours after cleavage (Supplementary Section IO), implying that any associated broadening of the flat-band states or modification of the superconducting gap should likewise develop progressively. Since we observe no corresponding time-dependent reconstruction of the low-energy spectra, adsorbates are unlikely to be the leading mechanism behind our observations. We also note that interaction-driven surface instabilities may also reconstruct the flat-band spectral weight. For instance, in our self-consistent calculations we find a small subdominant imaginary *s*-wave component localized near the edge (see Supplementary Section IN). This type of instability can split the zero-energy surface states, but since such an order remains subdominant to the primary *d*-wave gap, its characteristic energy scale is expected to be smaller than the superconducting gap. Consistently, after convolution with the experimental energy resolution, the subdominant component in our calculations does not visibly alter the flat-band peak. Other possible instabilities could in principle also split or redistribute the flat-band spectral weight. However, such orders are typically expected to develop at a secondary transition temperature below the superconducting transition temperature. This both reflects their small characteristic energy scale and suggests that a dominant instability-driven explanation should produce a measurable temperature-dependent reconstruction of the low-energy spectra. Since we do not observe such a reconstruction across the measured temperature range, these instabilities are unlikely to be the dominant mechanism responsible for our observations. A full quantitative treatment of all such states would require additional interaction channels and order parameters beyond the scope of the present work, which is to provide a minimal sufficient mechanism within our BdG framework.

Our work, however, highlights that this new cleaving technique is capable of producing surfaces with a topographic roughness that, within our experimental resolution and according to our microscopic calculations, is not sufficient to significantly lift the degeneracy of the surface states, thus highlighting the applicability of the technique to quasi-two-dimensional systems. Furthermore, from a measurement perspective, it indicates that the main challenge in detecting momentum-resolved signatures of these flat-band surface states by ARPES lies in the ability to resolve states characterized by a strong spatial inhomogeneity, which under certain circumstances might be pushed away from the topmost surface layers. On the other hand, from a materials point of view, our results demonstrate the importance of minimizing bulk disorder, for instance by studying materials with low defect densities and near-stoichiometric compositions. Thus, our results lay essential groundwork for future investigations of topological superconductors using ARPES, while also demonstrating how to study materials with surface-sensitive probes for which conventional mechanical cleavage is challenging.

## Methods

### Sample preparation

Single crystals were grown by the traveling solvent floating-zone method. Crystal growth was performed under an oxygen pressure of 2 amt. Post-growth annealing was conducted at 800 ^∘^C in an oxygen atmosphere for 100 h. The Strontium concentration was determined from the critical temperature^79,80^. Small pillars with approximate dimensions of 0.5 × 0.5 × 1.5 mm^3^ were cut from the resulting rod, with the longest axis aligned along the desired cleavage direction. To minimize the contact resistance between the sample and the sample stub, a thin layer of gold was deposited on the surface of the pillars, followed by an annealing treatment in an oxygen atmosphere at 1 bar for 1 h at 600 ^∘^C^81^. The samples were then mounted on copper stubs and fixed in place using EPO-TEK^®^ H21D silver epoxy, which was cured at 80 ^∘^C for $90\,\min$. The stub was subsequently mounted on a custom-made SEM extender, allowing for a manipulator tilt of up to −35^∘^. This geometry enabled perpendicular milling of all sides of the pillar without the need for repeated sample removal and remounting. To minimize possible local heating during the milling process, two strategies were employed: (*i*) milling was performed in parallel on opposite sides of the pillar (i.e., alternating milling cycles between opposing faces), and (*ii*) the maximum beam current was restricted to 500 nA and progressively reduced down to 1 nA as the cross-section of the pillar was thinned to approximately 150 × 150 μm^2^. The comparison of the superconducting critical temperature, obtained from the temperature dependence of the magnetic susceptibility of both the as-grown floating-zone rod and a small milled portion of the sample, revealed no significant change in onset temperature or overall behavior (Fig. 2b). Finally, the copper stub was directly mounted onto an ARPES sample plate, prepared for in-situ mechanical cleaving achieved by gently applying side pressure to the top of the pillar.

### ARPES

The ARPES measurements were performed at the I05 beamline of Diamond Light Source^82^ with an MBS A-1 hemispherical analyzer (MB Scientific AB) under an ultrahigh-vacuum pressure of approximately 1–2 × 10^−10^ mbar. The photon energies used were *hν* = 79 eV for the nodal cut, *hν* = 95 eV for the antinodal cut, and in the range of *hν* = 40–200 eV for the photon energy dependence maps. The sample temperature ranged from 6 K to 35 K. The combined energy resolution was approximately 3–4 meV with an approximate beam spot diameter (FWHM) of 40–50 μm. Several samples were measured over extended periods, as required for high-resolution measurements, by acquiring consecutive datasets that allowed us to track the temporal evolution of the spectra. Within the experimental reproducibility, we did not observe any systematic time-dependent degradation of the Fermi surface, disappearance of the dispersive bands, or emergence of new low-energy spectral features.

### Three-dimensional Fermi surface

The simulated three-dimensional Fermi surface of LSCO shown as a main-text visual comparison was generated using the one-band tight-binding parametrization of ref. ^62^. The hopping parameters were kept fixed to the literature values, and the chemical potential was set to *μ* = −393.3 meV in order to match the experimentally measured nodal Fermi momentum, *k*_*F*_ = −0.4605Å^−1^. This calculation was used only as a reference contour for visual comparison with the measured ARPES intensity. The surface doping was determined independently from the fitted experimental Fermi-surface area, as described in Supplementary Section IF.

### Tanuma-Powell-Penn formula

The TPP-2M formula^61^ allows us to obtain an estimate of the inelastic mean free path of photoemitted electrons based on a series of materials parameters. Using a density of 7 g/cm^3^ and a band gap of 20 meV, corresponding to the superconducting gap at the antinodes for *x* = 0.22^54^, we obtain an inelastic mean free path *λ* = 4.7413Å, which is in agreement with published estimates^83^. The inelastic mean free path describes the attenuation of the electron escape probability. Therefore, the corresponding wavefunction amplitude has an envelope *e*^−*z*/(2*λ*)^, and the squared modulus of the Fourier transform of this exponentially decaying amplitude is a Lorentzian with full width at half maximum equal to 1/*λ*. This allows us to obtain an estimate for the momentum broadening of Δ*k*_*z*_ ≈ 0.21 Å^−1^.

### Two-dimensional slab

In order to simulate the spectral function of the edge state, we constructed a single-orbital tight-binding model on a square lattice geometry defined over a cylindrical slab. Specifically, we imposed periodic boundary conditions along one spatial direction and open boundary conditions along the orthogonal direction, thereby generating two opposing edges oriented along the [11] crystallographic direction. The cylindrical slab comprised 280 sites in the direction perpendicular to the edge and 200 sites in the direction parallel to the edge. The Hamiltonian incorporated hopping terms up to the third nearest neighbor, with the corresponding hopping amplitudes obtained from previously published fitting results to ARPES data on samples with the same doping levels^63^. Furthermore, we used a nearest-neighbor pairing field whose local value was determined self-consistently (see the “Methods” section “Self-consistent Bogoliubov-de Gennes gap equation”). The geometric edge disorder was implemented using a representative line profile extracted from the AFM topography. To assess whether this profile was statistically representative of the measured surface morphology, we analyzed randomly selected AFM line cuts with the same physical length as the simulated edge (Supplementary Section IL). The line profile used in the main-text tight-binding calculation lies within the 10th–90th percentile range of this distribution for both the RMS roughness and the autocorrelation length, but was deliberately chosen from the lower-autocorrelation-length side of this representative interval. This choice provides a conservative upper estimate of the effect of the measured geometric edge disorder. For a fixed roughness amplitude, a shorter autocorrelation length corresponds to more rapidly varying edge morphology, enhancing large-momentum scattering and therefore maximizing the expected disorder-induced mixing of edge-state momenta. Consequently, if this relatively short-correlated profile does not produce sufficient broadening of the edge states, smoother profiles with longer autocorrelation lengths are unlikely to account for our observations.

Random on-site disorder was introduced through a mass term, given in the Nambu basis by

$${V}_{i}\,=\,{v}_{i}\,{\sigma }_{z},$$

where *σ*_*z*_ denotes the corresponding Pauli matrix and *v*_*i*_ is a site-dependent random variable drawn from a Gaussian distribution with a standard deviation equal to the disorder strength. The disorder spectra shown in the main text correspond to a single representative realization rather than to an ensemble average. This choice was made because the self-consistent calculation is performed on a 280 × 200 lattice, corresponding to 56,000 real-space sites and a BdG Hamiltonian of dimension 112,000, which must be diagonalized at each self-consistency iteration. The calculated ARPES spectral function is nevertheless partially self-averaging, since it is obtained from eigenstates extending along the full simulated edge and weighted over the photoelectron escape depth, rather than from the local density of states at a single site. The full Hamiltonian was then diagonalized in real space and, to account for the surface sensitivity inherent to ARPES measurements, the resulting eigenstates were weighted by an exponential function decaying into the bulk with a characteristic length scale equal to twice the inelastic mean free path of photoemitted electrons. This factor of two arises because the inelastic mean free path describes the attenuation of the squared modulus, whereas the weighting is applied to the eigenstate amplitudes. The inelastic mean free path was estimated using the TPP-2M formula; see the “Methods” Section “Tanuma-Powell-Penn formula.” Notice that because the lattice spacing for the discrete Fourier transform is comparable to the inelastic mean free path, some degree of aliasing artifacts is expected. Moreover, the finite size of the slab acts as a rectangular window, introducing spectral leakage. Both of these effects can result in a partial overestimation of the effective *k*_*z*_ broadening. Finally, in order to access the full momentum dependence probed by ARPES, we applied a two-dimensional Fourier transform to the exponentially weighted eigenstates—hence directly including a *k*_*z*_ broadening—and computed the momentum-dependent spectral function^84,85^, where in the retarded Green’s function we used a broadening of 3 meV to match the experimental energy resolution.

### Self-consistent Bogoliubov–de Gennes gap equation

The d-wave superconductivity can be captured within a mean-field treatment of the t-J model, where the quartic interaction term—omitting the density term—is decoupled in the pairing channel^86^

1 $${{{\mathcal{H}}}}_{J}=J{\sum}_{\langle ij\rangle }{{{\bf{S}}}}_{i}\cdot {{{\bf{S}}}}_{j}=-\frac{J}{2}{\sum}_{\langle ij\rangle }{\Delta }_{ij}{\varepsilon }_{\alpha \beta }{c}_{i,\alpha }^{{\dagger} }{c}_{j,\beta }^{{\dagger} }+{\Delta }_{ij}^{*}{\varepsilon }_{\alpha \beta }{c}_{j,\beta }{c}_{i,\alpha }-| {\Delta }_{ij}{| }^{2},$$

where *ε*_*αβ*_ is the Levi-Civita symbol with *ε*_*↑↓*_ = 1, and the spin-singlet pairing amplitude is defined as

2 $${\Delta }_{ij}=-\langle {c}_{i,\uparrow }{c}_{j,\downarrow }-{c}_{i,\downarrow }{c}_{j,\uparrow }\rangle .$$

In the absence of spin-orbit coupling, the system preserves SU(2) spin symmetry, and the Hamiltonian decouples into two identical singlet pairing blocks

3 $${{{\mathcal{H}}}}_{J}={{{\mathcal{H}}}}_{\uparrow \downarrow }\oplus {{{\mathcal{H}}}}_{\downarrow \uparrow }.$$

Consequently, the problem can be reformulated in a reduced Nambu basis, where for the Nambu spinor

$${\Psi }_{i}=\left(\begin{array}{c}{c}_{i\uparrow }\\ {c}_{i\downarrow }^{{\dagger} }\end{array}\right),$$

Eqs. (1) and (2) are:

4 $${{{\mathcal{H}}}}_{J}=J{\sum}_{\langle ij\rangle }{{{\bf{S}}}}_{i}\cdot {{{\bf{S}}}}_{j}=-\frac{J}{2}{\sum}_{\langle ij\rangle }{\Delta }_{ij}{c}_{i\uparrow }^{{\dagger} }{c}_{j\downarrow }^{{\dagger} }+{\Delta }_{ij}^{*}{c}_{j\downarrow }{c}_{i\uparrow }-| {\Delta }_{ij}{| }^{2},$$

and

5 $${\Delta }_{ij}=-\langle {c}_{i\uparrow }{c}_{j\downarrow }\rangle .$$

Neglecting the effects of the projection on the Hilbert space without doubly occupied sites, as well as the corrections to the kinetic terms and the chemical potential, we only need to impose self-consistency of the pairing gap^26^, which is done by solving the Bogoliubov–de Gennes self-consistent gap equation^87,88^

6 $${\Delta }_{ij}\,=\,V{\sum}_{{E}_{n} > 0}{u}_{n}(i)\,{v}_{n}^{*}(j)\,\tanh \left(\frac{{E}_{n}}{2{k}_{B}T}\right),$$

Where *V* denotes the effective pairing interaction, *u*_*n*_(*i*) and *v*_*n*_(*i*) are the local amplitudes of the Bogoliubov quasiparticles, and *E*_*n*_ are their corresponding eigenvalues. The value of *V* = 153 meV (*V* = 215 meV when the onsite disorder is introduced) was chosen so as to get a gap of approximately 24 meV at the antinodes, in approximate agreement with experimental values^54^.

In the numerical implementation, the pairing field is represented explicitly on nearest-neighbor bonds and bonds connected to removed boundary sites are excluded from the self-consistency loop. The initial guess is chosen to have a dominant ${d}_{{x}^{2}-{y}^{2}}$ sign structure, with opposite signs on the two inequivalent nearest-neighbor bond directions, together with a small imaginary extended-*s*-wave seed, similarly to what was done in other works^26^. This seed is used only to avoid artificially excluding a possible subdominant time-reversal-symmetry-breaking component. During the self-consistency loop, each complex nearest-neighbor bond amplitude is updated independently. Thus, the magnitude and phase of the bond-resolved pairing field are allowed to vary spatially near the boundary, near rough edge segments, and in the presence of onsite disorder. We do not impose a spatially uniform *d*-wave amplitude, nor do we project the solution onto a fixed *d*-wave or *d* + *is*-wave form during the iteration. The only projection applied after each update is the Hermitian consistency projection of the pairing, *Δ* → (*Δ* + *Δ*^†^)/2, which ensures that the matrix used in the Hamiltonian remains Hermitian and allows for a faster diagonalization.

To improve convergence to the fixed-point solution, we used a fixed-depth Anderson mixing scheme^64^ with history depth *m* = 5. At each iteration, the BdG gap update defines a nonlinear fixed-point map $F({\Delta }_{k})={\Delta }_{k+1}^{({{\rm{new}}})}$. The Anderson-extrapolated input and output fields were combined using a damping parameter *β*,

$${\Delta }_{k+1}=(1-\beta ){\Delta }_{k}^{(A)}+\beta {F}_{k}^{(A)}.$$

We used *β* = 0.7 for calculations with onsite disorder and *β* = 0.8 otherwise. Convergence was assessed using both the relative change in the order parameter and the relative fixed-point residual.

## Supplementary information

Supplementary Information

Transparent Peer Review file

## Data Availability

The primary data generated in this study and the main scripts used for the numerical calculations have been deposited in the Open Research Data Repository of the Max Planck Society under DOI 10.17617/3.UGDBUE, see ref. ^89^.
