# Supplementary material for: Suppression of spectral gap and flat bands on a cuprate superconductor side-surface
Source: Nat Commun. 2026 Oct 7;17:10573. doi: 10.1038/s41467-026-78020-y (PMC13645798; doi:10.1038/s41467-026-78020-y)
Supplement: Supplementary file 1 [file 41467_2026_78020_MOESM1_ESM.pdf]

Suppression of Spectral Gap and Flat Bands on a Cuprate Superconductor Side-Surface

Gabriele Domaine[1,2,*] Mihir Date[1,3], Sydney K. Y. Dufresne[1], Natalie Lehmann[1], Daiyu Geng[1], Tohru Kurosawa[4], Amit Kumar[1], Jiaju Wang[1], Tianlun Yu[1], Chien-Ching Chang[1], Swosti P. Sarangi[1], Ding Pei[5], Yiran Liu[2], Julia Küspert[6], Shigemi Terakawa[1,7,8], Markel Pardo Almanza[1], Jiabao Yang[1], Izabela Biało[6], Matthew D. Watson[3], Timur K. Kim[3], Stephen M. Hayden[9], Kritika Singh[10], Banabir Pal[1], Matteo Minola[2], Johan Chang[6], Naoki Momono[11], Migaku Oda[4], Stuart S. P. Parkin[1], Andreas P. Schnyder[2], and Niels B. M. Schröter[1,12, 13†]

[1]Max Planck Institut für Mikrostrukturphysik, Weinberg 2, 06120 Halle, Germany

[2]Max-Planck-Institut für Festkörperforschung, Heisenbergstrasse 1, D-70569 Stuttgart, Germany

[3]Diamond Light Source Ltd, Harwell Science and Innovation Campus, Didcot, OX11 0DE, United Kingdom

[4]Department of Applied Physics, Hokkaido University, Sapporo 060-8628, Japan

[5]Synchrotron SOLEIL, L'Orme des Merisiers, Saint-Aubin, 91190, France

[6]Physik-Institut, Universität Zürich, Winterthurerstrasse 190, CH-8057, Zürich, Switzerland

[7]Department of Applied Physics, Graduate School of Engineering, The University of Osaka, Osaka 565-0871, Japan

[8]Center for Future Innovation, Graduate School of Engineering, The University of Osaka, Osaka 565-0871, Japan

[9]H. H. Wills Physics Laboratory, University of Bristol, Bristol BS8 1TL, United Kingdom

[10]Institute of Materials Physics, Helmholtz-Zentrum Hereon, Geesthacht, 21502, Germany

[11]Faculty of Science and Engineering, Muroran Institute of Technology, Muroran 050-8585, Japan

[12]Martin-Luther-Universität Halle-Wittenberg, Institut für Physik, Von-Danckelmann-Platz 3, 06120, Halle (Saale), Germany and

[13]Halle-Berlin-Regensburg Cluster of Excellence CCE, Halle (Saale), Germany

(Dated: August 28, 2026)

CONTENTS

I. Supplementary 3
- A. Analytical expression of the edge-state wave function 3
- B. Out-of-plane momentum dependence 9
- C. Scattering sectors 9
- D. LAUE pattern 11
- E. Superconducting gap on the (001) surface for a sample ($x = 0.19$) prepared under the same conditions 12
- F. Estimation of the doping level 14
- G. EDC integration ranges 20
- H. Additional EDCs 21
- I. Spatially resolved density of states 22
- J. Effect of the gap suppression on the edge states 23
- K. Effect of a thin metallic layer 24
- L. AFM topography 25
- M. Different disorder strengths and realizations 27
- N. Minority s-wave component 29
- O. Shallow core-level photoemission spectra 30

References 33

* g.domaine@fkf.mpg.de

† niels.schroeter@mpi-halle.mpg.de

I. SUPPLEMENTARY

A. Analytical expression of the edge-state wave function

An effectively single-band d-wave superconductor belongs to the symmetry class CI. Since it is a gapless system, it is not possible to define a global topological invariant, however, away from the nodes, the presence of the chiral symmetry allows to define a group of maps $d_k : S^1 \mapsto \mathbb{R} \setminus \{0\} \simeq S^1$. For non-contractable loops $k \in S^1 \subseteq T^2$, the equivalence classes of the maps form the fundamental homotopy group $\pi_1 (\mathbb{R} \setminus \{0\}) = \mathbb{Z}$, where each class can be labeled by a winding number. A non-zero value of this topological invariant along a path with constant momentum parallel to an edge, denoted as $k_{\parallel}$, implies the presence of a zero-energy edge state with the same momentum $k_{\parallel}$ [1, 2].

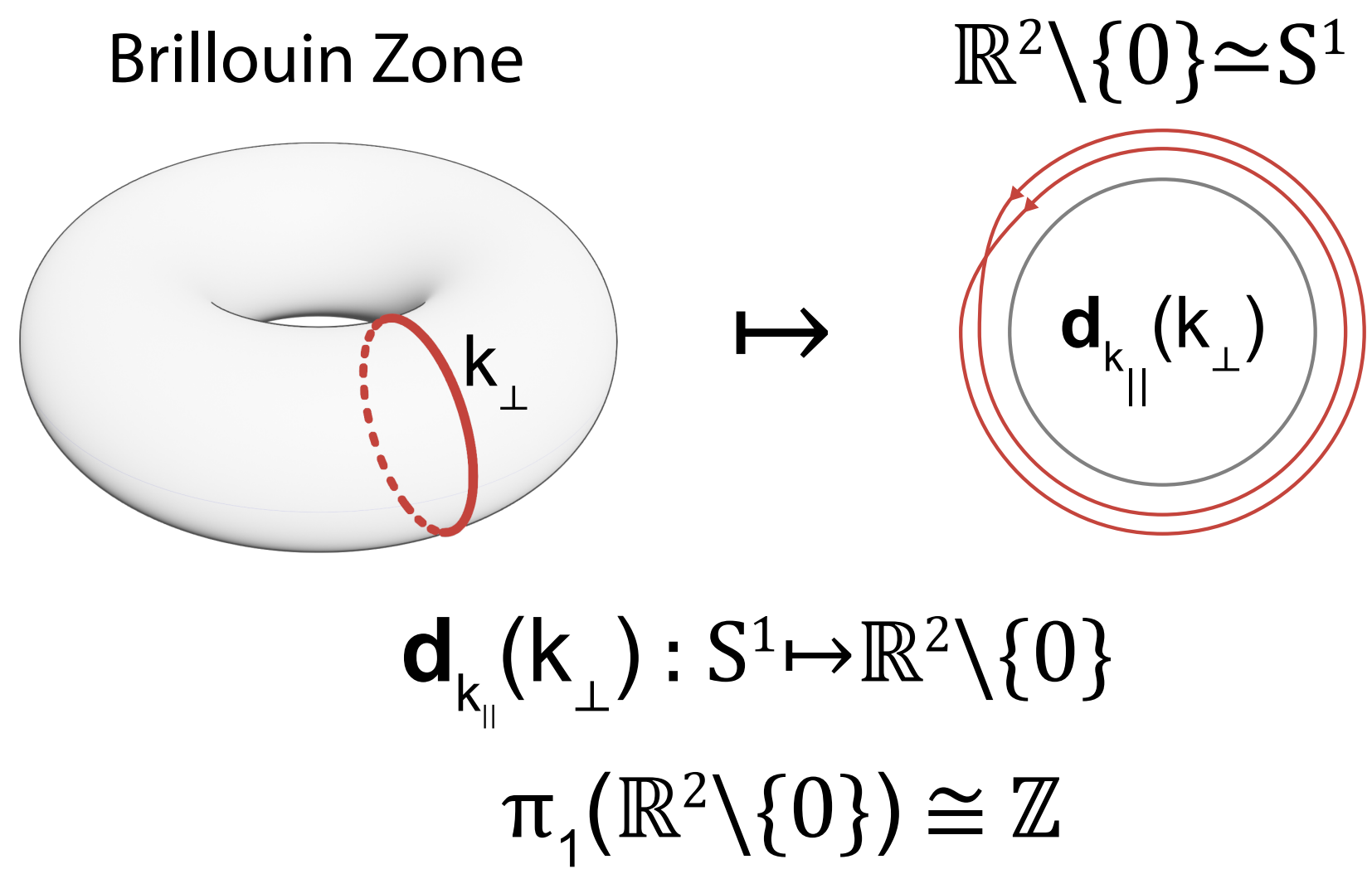

SUPPLEMENTARY FIG. 1. Let $X \coloneqq [-\pi, \pi] \times [-\pi, \pi]$ be a square Brillouin zone with the equivalence relation $(k_{\parallel}, -\pi) \sim (k_{\parallel}, \pi)$ and $(-\pi, k_{\perp}) \sim (\pi, k_{\perp})$. Then the quotient space $X/\sim$ is a T^2 torus. Then paths with a constant momentum component are topologically equivalent to non-contractable S^1. Each map d_k is then connecting the elements of these loops to the elements of another S^1 loop.

A single-band class CI superconductor can be described by the following Bogoliubov-de Gennes (BdG) Hamiltonian:

$$\mathcal{H}(\boldsymbol{k}) = \frac{1}{2} \sum_{\boldsymbol{k}} (c^{\dagger}_{\boldsymbol{k}\uparrow}, c_{-\boldsymbol{k}\downarrow}) \mathcal{H}_{BdG}(\boldsymbol{k}) \begin{pmatrix} c_{\boldsymbol{k}\uparrow} \\ c^{\dagger}_{-\boldsymbol{k}\downarrow} \end{pmatrix} \tag{1}$$

$$\mathcal{H}_{BdG}(\boldsymbol{k}) = \begin{pmatrix} \varepsilon_{\boldsymbol{k}} & \Delta_{\boldsymbol{k}} \\ \Delta_{\boldsymbol{k}} & -\varepsilon_{\boldsymbol{k}} \end{pmatrix} \tag{2}$$

Where for a square lattice belonging to the two-dimensional point group D_4 we have:

$$\varepsilon_{\boldsymbol{k}} = -4t \cos\left(k_{\parallel}\frac{\delta}{2}\right)\cos\left(k_{\perp}\frac{\delta}{2}\right) - \mu \tag{3}$$

$$\Delta_{\boldsymbol{k}} = -4|\Delta| \sin\left(k_{\parallel}\frac{\delta}{2}\right)\sin\left(k_{\perp}\frac{\delta}{2}\right) \tag{4}$$

Here, for convenience, we have rotated the square lattice so that $\delta = a\sqrt{2}$, with a being the lattice spacing between two nearest neighbours.

By construction, this BdG Hamiltonian has a spinless time-reversal symmetry $\mathcal{T} = \mathcal{K}$, where $\mathcal{K}$ is the complex conjugation operator, and a charge conjugation or particle-hole symmetry $\mathcal{C} = \sigma_y \mathcal{K}$. Combining these two symmetries we additionally obtain a chiral symmetry which anticommutes with the BdG Hamiltonian:

$$\{\Gamma, \mathcal{H}_{BdG}(\boldsymbol{k})\} = 0 \;, \qquad \Gamma = \sigma_y \tag{5}$$

By diagonalizing the chiral operator we can obtain the unitary matrix U_Γ that maps an operator to its chiral basis representation.

$$U_\Gamma^\dagger \Gamma U_\Gamma = \sigma_z \;, \qquad U_\Gamma = \frac{1}{\sqrt{2}}\begin{pmatrix} 1 & 1 \\ i & -i \end{pmatrix} \tag{6}$$

So that we can bring $\mathcal{H}_{BdG}$ into the following off-diagonal form:

$$\begin{aligned} \mathcal{H}_{BdG}^{(\Gamma)}(\boldsymbol{k}) &= U_\Gamma^\dagger \mathcal{H}_{BdG}(\boldsymbol{k}) U_\Gamma \\ &= \begin{pmatrix} 0 & q_{\boldsymbol{k}} \\ q_{\boldsymbol{k}}^* & 0 \end{pmatrix} \\ &= \begin{pmatrix} \Re(q_{\boldsymbol{k}}) \\ -\Im(q_{\boldsymbol{k}}) \end{pmatrix} \cdot \boldsymbol{\sigma} \\ &= \boldsymbol{d}_{\boldsymbol{k}} \cdot \boldsymbol{\sigma} \end{aligned} \tag{7}$$

Where $q_{\boldsymbol{k}} = \varepsilon_{\boldsymbol{k}} - i\Delta_{\boldsymbol{k}}$

This simply amounts to perform a change of basis from the particle-hole basis to the chiral basis:

$$\begin{aligned} U_\Gamma^\dagger \begin{pmatrix} c_{\boldsymbol{k}\uparrow} \\ c_{-\boldsymbol{k}\downarrow}^\dagger \end{pmatrix} &= \frac{1}{\sqrt{2}} \begin{pmatrix} c_{\boldsymbol{k}\uparrow} - ic_{-\boldsymbol{k}\downarrow}^\dagger \\ c_{\boldsymbol{k}\uparrow} + ic_{-\boldsymbol{k}\downarrow}^\dagger \end{pmatrix} \\ &= \begin{pmatrix} \gamma_{\boldsymbol{k},+} \\ \gamma_{\boldsymbol{k},-} \end{pmatrix} = \boldsymbol{\gamma}_{\boldsymbol{k}} \end{aligned} \tag{8}$$

Where $\gamma_{\boldsymbol{k},+}$ and $\gamma_{\boldsymbol{k},-}$ are annihilation operators of quasiparticles with chirality +1 and -1, respectively.

Using 7 and 8, the Hamiltonian of 1 can be rewritten as:

$$\mathcal{H}(\boldsymbol{k}) = \frac{1}{2} \sum_{\boldsymbol{k}} \boldsymbol{\gamma}_{\boldsymbol{k}}^\dagger \mathcal{H}_{BdG}^{(\Gamma)}(\boldsymbol{k}) \boldsymbol{\gamma}_{\boldsymbol{k}} \ . \tag{9}$$

We now want to use this result to get an expression for the in-gap states wave function. To do so, we begin by noting that the eigenvalue equation $\mathcal{H}_{BdG}\Psi_{k_\parallel} = E_{BdG}\Psi_{k_\parallel}$, for energies satisfying $|E_{BdG}| < \Delta_{\boldsymbol{k}}$, only allows for exponentially decaying solutions. These eigenstates are obtained by allowing the crystal momentum to take on complex values. Thus, we can devise the following ansatz consisting of a linear superposition of plane waves that decay exponentially into the bulk with a characteristic lengthscale $\xi = \frac{1}{\Re(\kappa^{\gamma\eta})}$:

$$\Psi_{k_\parallel, -i\kappa_m^n}(r_\parallel, r_\perp) = \sum_{n,m} C_m^n \psi_m^n e^{\kappa_m^n r_\perp} e^{ik_\parallel r_\parallel} \ . \tag{10}$$

Because the flat-bands are pinned at the Fermi level, we can get the allowed values for κ by solving the secular equation $\mathcal{H}_{BdG}^{(\Gamma)}(k_\parallel, -i\kappa_m^n)\Psi_{k_\parallel}(r_\parallel, r_\perp) = 0$, where we have used the momentum operator $-i\partial_r$. After expanding the trigonometric functions to second order in $\boldsymbol{k}$, we get two solutions for $q_{\boldsymbol{k}}$ and two solutions for $q_{\boldsymbol{k}}^*$:

$$\kappa^{\gamma\eta} = -\gamma \frac{|\Delta|}{t} k_\parallel + (-1)^{\eta+1} \frac{1}{t} \sqrt{\left(|\Delta|^2 + t^2\right) {k_\parallel}^2 - \frac{t(4t+\mu)}{a^2}} \tag{11}$$

Where $\gamma \in \{+,-\}$ and $\eta \in \{1,2\}$. The four solutions are plotted in Figure 2. Here γ indicates the eigenvalues of the chiral symmetry operator, since $q_{\boldsymbol{k}}$ and $q_{\boldsymbol{k}}^*$ respectively yield the solutions with chirality -1 and $+1$, spanned by $\gamma_{\boldsymbol{r},-}^\dagger |0\rangle$ and $\gamma_{\boldsymbol{r},+}^\dagger |0\rangle$ according to $\Gamma\gamma_{\boldsymbol{r},\pm}^\dagger |0\rangle = \pm\gamma_{\boldsymbol{r},\pm}^\dagger |0\rangle$. This leads to:

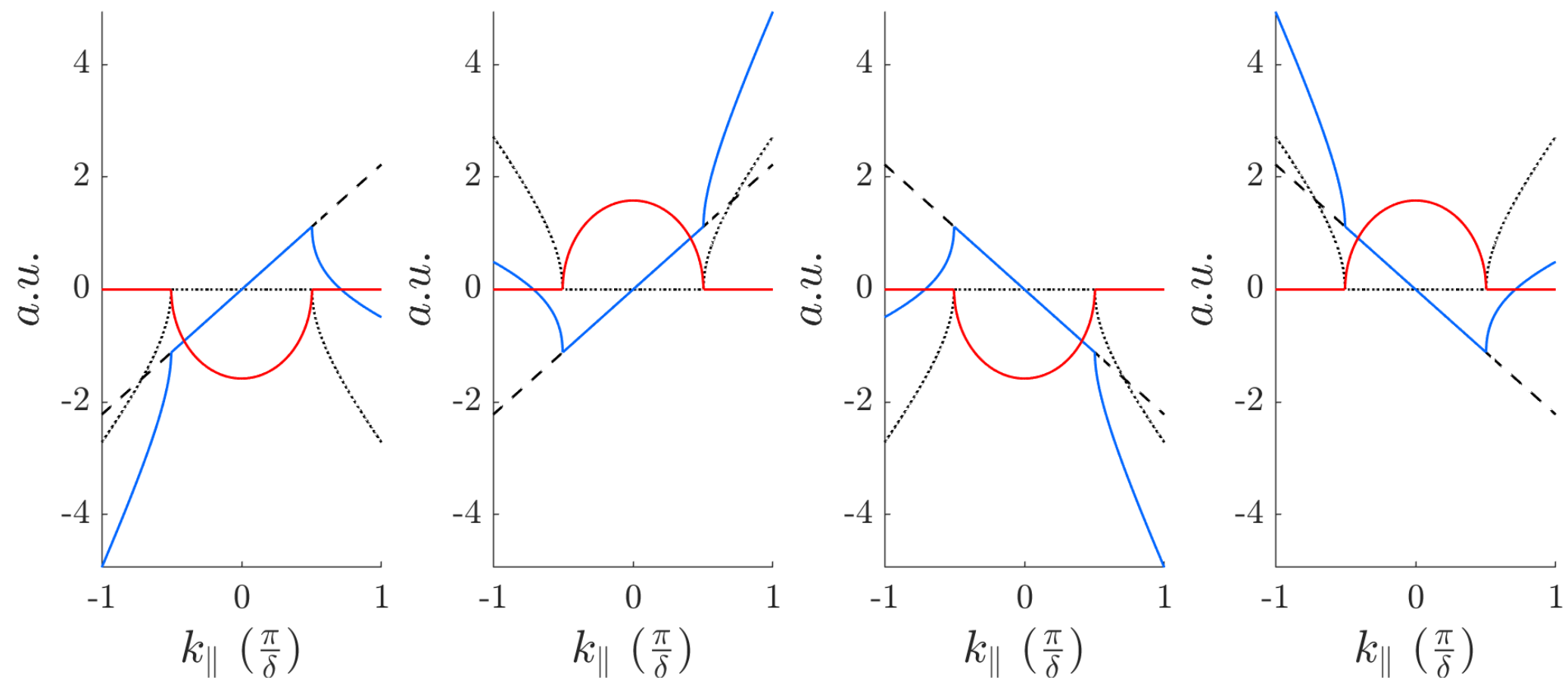

SUPPLEMENTARY FIG. 2. Real (blue) and imaginary parts (red) of the four allowed $\kappa^{\gamma\eta}$ for $|\Delta| = t$. The dashed and dotted lines show the two contributions to the real part, coming respectively from the first and second term in 11.

$$\Psi_{k_\parallel}(\boldsymbol{r}) = \sum_{\gamma,\eta} C_{k_\parallel}^{\gamma\eta} e^{\boldsymbol{K}\cdot\boldsymbol{r}} \gamma_{\boldsymbol{r},\gamma}^\dagger |0\rangle \tag{12}$$

$$= \sum_{\gamma} \varphi_{k_\parallel}^{\gamma} \gamma_{\boldsymbol{r},\gamma}^\dagger |0\rangle \,. \tag{13}$$

Where:

$$\boldsymbol{K} = \begin{pmatrix} K_\parallel \\ K_\perp \end{pmatrix} = \begin{pmatrix} 0 \\ \mathfrak{R}\left(\kappa^{\gamma\eta}\right) \end{pmatrix} + i \begin{pmatrix} k_\parallel \\ \mathfrak{I}\left(\kappa^{\gamma\eta}\right) \end{pmatrix} \,, \tag{14}$$

$$\boldsymbol{r} = \begin{pmatrix} r_\parallel \\ r_\perp \end{pmatrix} . \tag{15}$$

To determine the coefficients $C_{k_\parallel}^{\gamma\eta}$ we focus on a semi-infinite system with $r_\perp > 0$ and an edge at $r_\perp = 0$, upon imposing that $\Psi_{k_\parallel, -i\kappa^{\gamma\eta}}(r_\parallel, r_\perp = \infty) = 0$ we find the following conditions:

$$\begin{cases} C_{k_\parallel}^{-\eta} = 0 & k_\parallel \in [-k_F, 0] \\ C_{k_\parallel}^{+\eta} = 0 & k_\parallel \in [0, k_F] \end{cases} \tag{16}$$

Furthermore the system has a time reversal symmetry, which in the chiral basis takes the form $\mathcal{T}_\Gamma = U_\Gamma^\dagger \mathcal{T} U_\Gamma = \sigma_x \mathcal{K}$. After applying this operator to the wave function, we get the conditions:

$$\begin{cases} \varphi^{+}_{k_\parallel} = \left(\varphi^{-}_{k_\parallel}\right)^{*} \\ \varphi^{-}_{k_\parallel} = \left(\varphi^{+}_{k_\parallel}\right)^{*} \end{cases} \tag{17}$$

Lastly, the semi-infinite system also has a mirror plane $\sigma_v = \sigma_x \mathcal{K}_\parallel$ perpendicular to the edge, where the subscript denotes the fact that the complex conjugation operator acts only on $K_\parallel$. So that we get:

$$\begin{cases} \varphi^{+}_{k_\parallel} = \mathcal{K}_\parallel \varphi^{-}_{k_\parallel} \\ \varphi^{-}_{k_\parallel} = \mathcal{K}_\parallel \varphi^{+}_{k_\parallel} \end{cases} \tag{18}$$

Combining 16, 17 and 18 we obtain:

$$\sin\left(\Im\left(\kappa^{\gamma 1}\right)\right)\left(C^{\gamma 1}_{k_\parallel} e^{\Re\left(\kappa^{\gamma 1}\right)} - C^{\gamma 2}_{k_\parallel} e^{\Re\left(\kappa^{\gamma 2}\right)}\right) = 0. \tag{19}$$

Where we used the fact that $\Im\left(\kappa^{+}_{1}\right) = (-1)^{\alpha+1}\Im\left(\kappa^{\gamma\eta}\right)$. Because $\Im\left(\kappa^{\gamma 1}\right) = 0$ in the interval $k^{*}_{\parallel} = \sqrt{\frac{2t(4t+\mu)}{\delta^2(|\Delta|^2+t^2)}} \leq |k_\parallel| \leq \sqrt{\frac{2(4t+\mu)}{t\delta^2}} = k_F$, the equation is always verified in this range. On the other hand, when $\Im\left(\kappa^{\gamma 1}\right) \neq 0$ we have that $\Re\left(\kappa^{\gamma 1}\right) = \Re\left(\kappa^{\gamma 2}\right)$, so that $C^{\gamma 1}_{k_\parallel} = C^{\gamma 2}_{k_\parallel}$. We can then impose that the wave function be continuous and differentiable at $k^{*}_{\parallel}$:

$$\left.\varphi^{\gamma}_{|k_\parallel|<k^{*+}_\parallel}\right|_{k_\parallel = k^{*-}_\parallel} = \left.\varphi^{\gamma}_{|k_\parallel|>k^{*+}_\parallel}\right|_{k_\parallel = k^{*+}_\parallel} \tag{20}$$

$$\left.\frac{d}{dk_\parallel}\varphi^{\gamma}_{|k_\parallel|<k^{*+}_\parallel}\right|_{k_\parallel = k^{*-}_\parallel} = \left.\frac{d}{dk_\parallel}\varphi^{\gamma}_{|k_\parallel|>k^{*+}_\parallel}\right|_{k_\parallel = k^{*+}_\parallel}, \tag{21}$$

which ultimately requires that $C^{\gamma 1}_{k_\parallel} = C^{\gamma 2}_{k_\parallel}$ be verified for all $k_\parallel$. Finally by using again 18 we obtain that:

$$C_{k_\parallel} = C^{+\eta}_{k_\parallel} = C^{-\eta}_{-k_\parallel}. \qquad \square \tag{22}$$

Because for each state identified by $k_\parallel$ there can only be one chiral fermion, we can get an expression for $C_{k_\parallel}$ by imposing that:

$$1 = \int_0^\infty \left|\Psi^{\gamma}_{k_\parallel}(\boldsymbol{r})\right|^2 dr = |C|^2 \sum_{\eta,\eta'} \left(-\frac{1}{2\left(\kappa^{\gamma\eta} + \kappa^{\gamma\eta'\,*}\right)}\right)$$

$$C = \begin{cases} \left[\sum_{\eta,\eta'}\left(-\frac{1}{2(\kappa^{-,\eta}+\kappa^{-,\eta'\,*})}\right)\right]^{-\frac{1}{2}}, & -k_F < k_\parallel < 0, \\ \left[\sum_{\eta,\eta'}\left(-\frac{1}{2(\kappa^{+,\eta}+\kappa^{+,\eta'\,*})}\right)\right]^{-\frac{1}{2}}, & 0 < k_\parallel < k_F. \end{cases}$$

The wave function that we obtained is plotted in Figure 3.

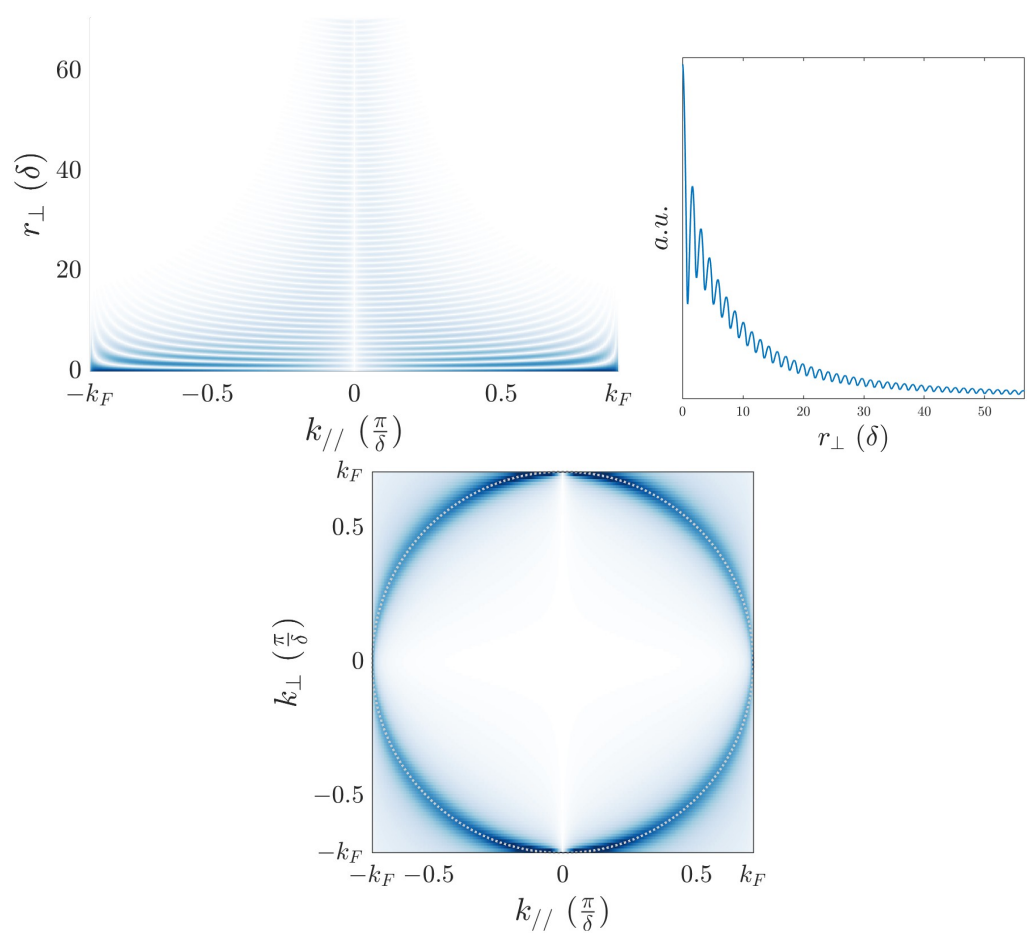

SUPPLEMENTARY FIG. 3. (Top left) Absolute value of the Fourier transform of the wave function along $k_\parallel$, $\mathcal{F}_{k_\parallel}\left(\Psi_{k_\parallel}(\boldsymbol{r})\right) = \Psi(k_\parallel, r_\perp)$ and its integration along $k_\parallel$ (top right). (Bottom) Absolute value of the full Fourier transform of the wave function $\mathcal{F}_{\boldsymbol{k}}\left(\Psi_{k_\parallel}(\boldsymbol{r})\right) = \Psi(\boldsymbol{k})$. In all cases we used $|\Delta|/t = 0.1$.

B. Out-of-plane momentum dependence

Even though we do not impose periodic boundary conditions in the direction perpendicular to the edge, in practice, because for most superconductors $\Delta \ll t$, to a first approximation, one can still obtain a relatively well defined momentum perpendicular to the edge. In fact, if we look at the Fourier transform of $e^{\kappa^{\gamma\eta} r_\perp}$:

$$\mathcal{F}\left(e^{\kappa^{\gamma\eta} r_\perp}\right) = \frac{2\Re\left(\kappa^{\gamma\eta}\right)}{\Re\left(\kappa^{\gamma\eta}\right)^2 + k_\perp^2} * \delta\left(k_\perp - \Im\left(\kappa^{\gamma\eta}\right)\right), \tag{23}$$

where to limit ourselves to real expressions we have also included the solutions for $r_\perp < 0$ satisfying $\Psi_{k_\parallel}(r_\parallel, r_\perp = \infty) = 0$, we see that we simply have a convolution of a Lorentzian $\mathcal{L}(k_\perp; 0, \Re\left(\kappa^{\gamma\eta}\right))$ and a Dirac delta function. Using the sifting property of the Dirac delta function and the fact that $\lim_{\gamma\to 0} \mathcal{L}(x; x_0, \gamma) = \delta\left(x - x_0\right)$, we get that:

$$\lim_{\Delta/t\to 0} \mathcal{F}\left(e^{\kappa^{\gamma\eta} r_\perp}\right) = \delta\left(k_\perp - \Im\left(\kappa^{\gamma\eta}\right)\right), \tag{24}$$

which means that, as expected, for small gap-to-bandwidth ratios, the dependence of the spectral weight on $k_\perp$ becomes non-negligible. By equating Equation 3 to zero, we can derive the expression of the perpendicular component of the Fermi momentum:

$$k_\perp^F = \frac{1}{t}\sqrt{t^2 \frac{t(4t+\mu)}{a^2} - k_\parallel^{\;2}}. \tag{25}$$

We can then easily verify the equivalence:

$$\lim_{\Delta/t\to 0} \Im\left(\kappa^{\gamma\eta}\right) = k_\perp^F. \tag{26}$$

This means that in the limit of a small superconducting gap, the spectral weight of the edge state is transferred to the Fermi surface of the normal state. This is directly visible in Fig. 3, where because of the expansion to second order, the Fermi surface is a circle.

C. Scattering sectors

Consider a random onsite disorder acting on each site of the form expressed in the chiral basis:

$$V_q = \sum_{\boldsymbol{r}} v(\boldsymbol{r})\, \sigma_x\, e^{-i\boldsymbol{q}\cdot\boldsymbol{r}}, \tag{27}$$

we can verify that it preserves the chiral symmetry of the Hamiltonian 2 by computing its anticommutation relation with the chiral symmetry operator which in this basis is simply σ_z:

$$\{\sigma_z, \sigma_x\} = 0. \tag{28}$$

The chirality eigenstates are:

$$|+\rangle = \begin{pmatrix} 1 \\ 0 \end{pmatrix}, \quad |-\rangle = \begin{pmatrix} 0 \\ 1 \end{pmatrix}.$$

So that the matrix elements of σ_x are:

$$\langle\gamma|\sigma_x|\gamma'\rangle = 1 - \delta_{\gamma,\gamma'}, \qquad \gamma \in \{+,-\}. \tag{29}$$

Thus a random onsite potential that preserves the chiral symmetry of the Hamiltonian mediates scattering only between states of opposite chirality. Scattering within the same chirality sector is instead forbidden.

D. LAUE pattern

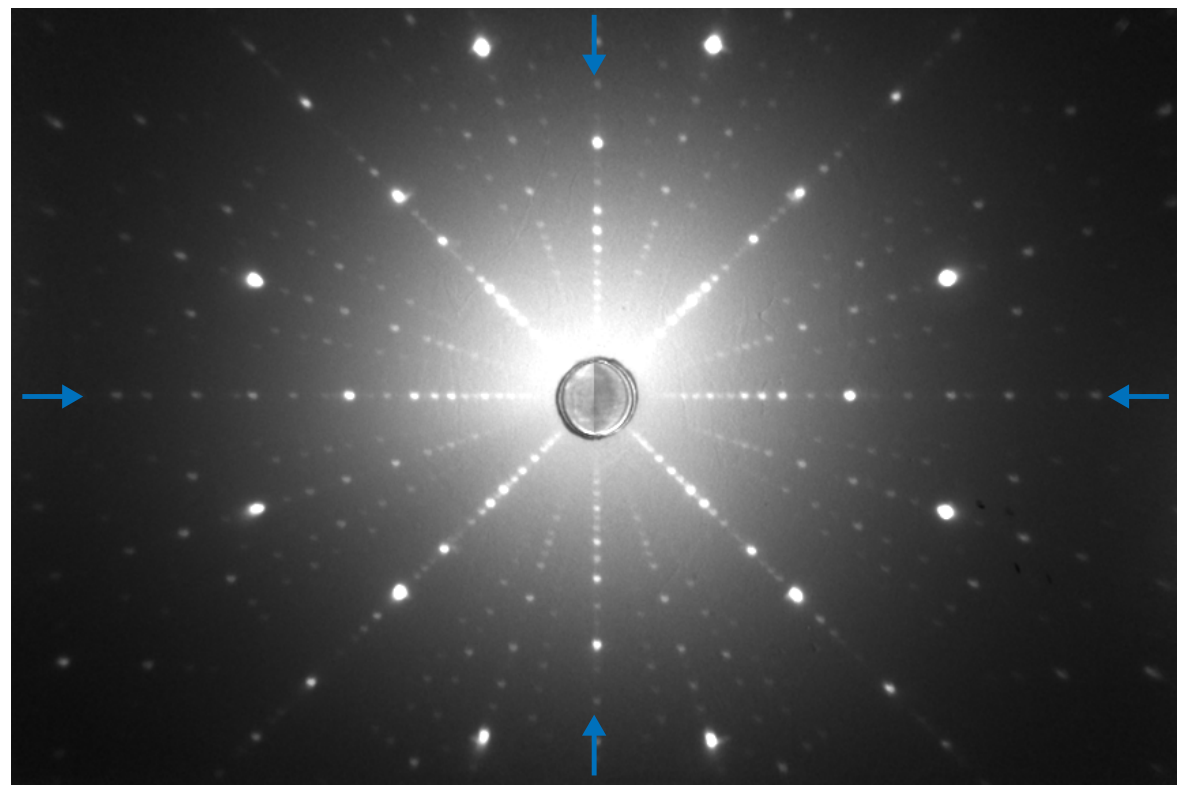

SUPPLEMENTARY FIG. 4. Laue back-scattering diffraction pattern from a (001) surface used to orient the crystals along the [110] direction, indicated by the blue arrows.

E. Superconducting gap on the (001) surface for a sample ($x = 0.19$) prepared under the same conditions

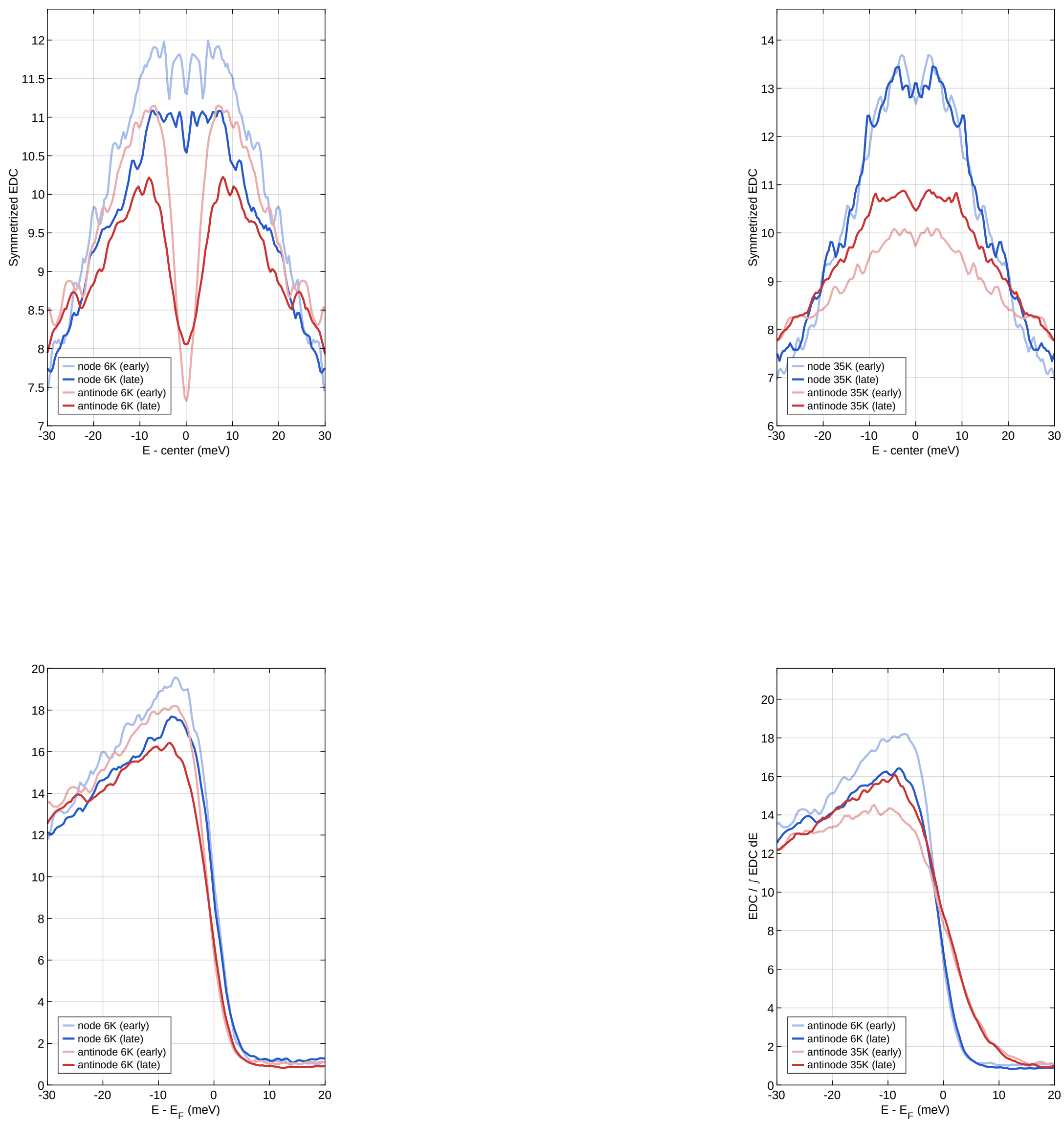

SUPPLEMENTARY FIG. 5. **Gap-related spectral feature on a reference** (001) **surface.** ARPES spectra from a reference LSCO sample prepared under comparable conditions show a resolvable gap-related feature on the conventional (001) surface. This indicates that the sample-preparation procedure does not generically preclude the observation of superconducting gap features in ARPES.

All spectra were first corrected for the instrumental Fermi-edge shape using a gold reference measured under identical conditions. Energy distribution curves (EDCs) at the nodal points were extracted in the second Brillouin zone, while both nodal and antinodal spectra were measured with LH polarization at a photon energy of 65 eV with a combined resolution of approximately 4-5 meV. The nodal spectra were used as an internal reference in order to minimize differences in space-charge and conventional charging effects between the gold reference and the sample. Accordingly, the leading-edge midpoint (LEM) of the nodal EDCs at 6 K (and analogously at 35 K) were mutually aligned, and the corresponding shifts were applied to the antinodal spectra. The measurements were performed over two temperature cycles ("early" and "late"), and gold reference spectra were acquired throughout the experiment; fitting the Fermi edge with a Fermi–Dirac distribution convoluted with a Gaussian resolution function yielded a standard error of 0.125 meV, allowing the Fermi level to be treated as effectively constant. Additional flux-dependence measurements on the gold reference and on the sample at the nodes and antinodes were used to extrapolate the LEM and EF to zero flux, correcting for residual space-charge effects by 0.49 meV and 0.61 meV, respectively. The corrected spectra were subsequently symmetrized, yielding a gap of approximately 7 meV from the quasiparticle peak positions in the symmetrized EDCs. While this value is somewhat smaller than some reported in the literature obtained by fitting the symmetrized EDCs with a self-energy–based spectral function including resolution broadening [3], it is comparable to others [4], where the gap was extracted by Fermi-function and normal-state division rather than symmetrization. Given the strong sensitivity of both the symmetrization and the division to small E_F shifts, we consider our results to be consistent with previous reports, with the additional observation of a small LEM shift of a couple of meV.

F. Estimation of the doping level

We estimated the carrier concentration of the measured LSCO side surface from the Fermi-surface area obtained by fitting the ARPES intensity map with a one-band tight-binding model. The procedure follows the Luttinger-count approach, where the Fermi surface fixes the occupied band area and therefore the hole doping of the single CuO_2-derived band.

The ARPES intensity was first integrated over 16 meV energy windows and the experimental isoenergy contours were extracted. The resulting points were fitted to the one-band LSCO tight-binding dispersion defined in Ref. [5] with the addition of a rigid momentum shift, an intrinsic band-anisotropy parameter, and small affine and quadratic corrections to the momentum conversion, so as to account for small distortions of the experimental data. These additional parameters were therefore allowed in the fitting step, but removed before performing the Luttinger count.

The objective function was dominated by the Fermi-level slice. The residuals from deeper binding-energy slices were exponentially down-weighted, so that these points stabilize the local curvature and bandwidth without forcing the low-energy one-band model to reproduce the higher-binding-energy dispersion where the measured bands are affected by renormalization effects and deviate from the minimal tight-binding description. The point residuals were evaluated in a contour-distance form,

$$r_i = \sqrt{w(E_i)}\frac{E_{\mathrm{TB}}(\mathbf{k}_i) - E_i}{\sqrt{\sigma_E^2 + \left(\sigma_k|\nabla_{\mathbf{k}}E_{\mathrm{TB}}|\right)^2}}, \tag{30}$$

where $E_{\mathrm{TB}}(\mathbf{k}_i) - E_i$ is the energy mismatch between the tight-binding model and the extracted experimental point i. The factor $w(E_i)$ is the energy-slice weight: it is largest for the Fermi-level slice and decreases for the finite-binding-energy slices, so that the fit is primarily controlled by the measured Fermi surface while the deeper slices act only as weak constraints on the local dispersion and bandwidth. The quantities $\sigma_E = 5$ meV and $\sigma_k = 0.030$ Å^{-1} are approximate experimental uncertainties in the energy and momentum position of the extracted contour points, respectively. Thus the denominator is the effective energy uncertainty associated with point i. It contains the direct energy uncertainty, σ_E, and the energy uncertainty generated by a finite momentum uncertainty, σ_k, which is proportional to the local slope of the band. The two uncertainty contributions are added in quadrature because under the assumption that they represent independent sources of error.

This normalization avoids an artificial preference for solutions with an unphysically small bandwidth. With a pure energy residual, the optimizer can reduce the apparent energy mismatch by flattening the band, even if the model contour is not a better representation of the measured Fermi surface. The contour-distance residual instead measures the mismatch relative to the uncertainty with which an isoenergy contour can be located in momentum space.

Because as mentioned above we are restricted to points close to the Fermi level (in our

case between 0 meV and −50 meV), the fitting problem is underconstrained, especially with respect to the overall bandwidth. To circumvent this limitation without imposing a fixed bandwidth, we weakly anchored the in-plane hopping parameters to the dressed LSCO values of Ref. [5]. The remaining additional parameters, including the k_z-related terms, rigid momentum shifts, and momentum-warp terms, were anchored to zero. These terms therefore act as regularized corrections that can account for small global distortions of the experimental momentum conversion, without replacing the physical tight-binding dispersion. Finally, the fit favored solutions in which the largest hopping remains the nearest-neighbor in-plane hopping. The k_z-related terms were further constrained by soft penalties for $|t_z| > 0.25|t|$, $|t_{zp}| > 0.15|t|$, and $|t_{zp}| > |t_z|$. Thus, this regularization allows the model parameters to vary in order to accommodate the measured Fermi surface, but prevents collapse to an unphysically small bandwidth or to an excessively large out-of-plane correction.

In addition to these soft penalties, the final solution was required to have a physically acceptable Fermi-surface topology. The accepted solutions were those with a single closed pocket per active Brillouin zone (either hole- or electron-like) and no additional unresolved or spurious pockets. This constraint eliminates isolated low-objective solutions in which the fit creates extra Fermi-surface sheets that are not expected for LSCO and are not observed experimentally. Thus, in order to favour a convergence to these physical solution, we included a soft no-pocket penalty around the center of the active Γ-plane Brillouin zones to suppress spurious near-Γ low-energy contours.

After fitting, the hole doping was computed from the occupied area of the fitted band. For each Brillouin-zone polygon, the occupied fraction was determined by counting the fraction of a dense k-space grid for which $E_{\mathrm{TB}}(\mathbf{k}) < 0$. In the extended-zone geometry used here, the effective occupied fraction was obtained by averaging the occupied fractions of the Γ- and Z-plane polygons,

$$f_{\mathrm{occ}} = \frac{1}{2}\left(\frac{A_\Gamma(E_{\mathrm{TB}} < 0)}{A_{\mathrm{BZ},\Gamma}} + \frac{A_Z(E_{\mathrm{TB}} < 0)}{A_{\mathrm{BZ},Z}}\right). \tag{31}$$

For a spin-degenerate single band, the electron filling is $n = 2f_{\mathrm{occ}}$, so that the hole doping relative to half filling is

$$p = 1 - 2f_{\mathrm{occ}}. \tag{32}$$

In order to avoid an initial guess bias, we repeated the fit from 96 different initial parameter sets. Many random starts converged to the same physical doping basin centered at $p \simeq 0.19$, and the best low-objective physical solutions agree within approximately 10^{-3} in p. This convergence indicates that the inferred doping is not set by a single fine-tuned initial condition, but by the Fermi-surface area selected by the experimental data and the physical topology constraints.

This obtained doping value should be regarded as an estimate of the doping regime rather than a high-precision determination of the surface carrier density. Small deviations between the Luttinger count and the nominal hole doping have already been observed in ARPES

studies of the conventional (001) surface of LSCO [6]. In the present case, the side-surface geometry introduces an additional uncertainty because the photon-energy map is affected by finite k_z-broadening effects. For this reason, the extracted value is compatible with the nominal doping $p = 0.22$ of our sample and places the surface confidently within the superconducting region of LSCO.

To estimate the uncertainty of the Luttinger count, we constructed a weighted ensemble of tight-binding solutions around the selected best fit. Candidate parameter sets were generated by perturbing the fitted tight-binding and momentum-conversion parameters within small proposal widths around the selected solution. Each candidate was then compared to the selected fit at the experimental contour points in the full fitted momentum frame, where the ARPES data constrain the model.

For each candidate model, we computed the energy difference ΔE_i from the selected model at every fitted point i. This difference was normalized by the local experimental uncertainty expected from both energy and momentum resolution,

$$d_i = \frac{|\Delta E_i|}{\sqrt{\sigma_E^2 + \left(\sigma_{k_x} \frac{\partial E_{\mathrm{TB}}}{\partial k_x}\right)^2 + \left(\sigma_{k_y} \frac{\partial E_{\mathrm{TB}}}{\partial k_y}\right)^2}}, \tag{33}$$

where the derivatives were evaluated for the selected tight-binding solution. For the uncertainty estimate shown in Fig. 8, we used $E_{\mathrm{FWHM}} = 5$ meV, $k_{x,\mathrm{FWHM}} = 0.200$ Å^{-1}, and $k_{y,\mathrm{FWHM}} = 0.050$ Å^{-1}, converted to Gaussian widths according to $\sigma = \mathrm{FWHM}/2.355$.

Rather than imposing a hard cutoff, each candidate was assigned a continuous compatibility weight

$$W = \exp\left[-\frac{1}{2}\left(\max_i d_i\right)^2\right]. \tag{34}$$

Thus, solutions whose calculated contours remain within the experimental uncertainty of the selected fit receive large weight, while solutions requiring displacements larger than the experimental resolution are exponentially suppressed. For each weighted candidate, the intrinsic Luttinger count was recomputed. The resulting weighted doping distribution was then fitted with a skew-normal distribution. The 95% confidence interval gives $0.130 < p < 0.267$, with a central value near the selected multistart result $p \simeq 0.19$.

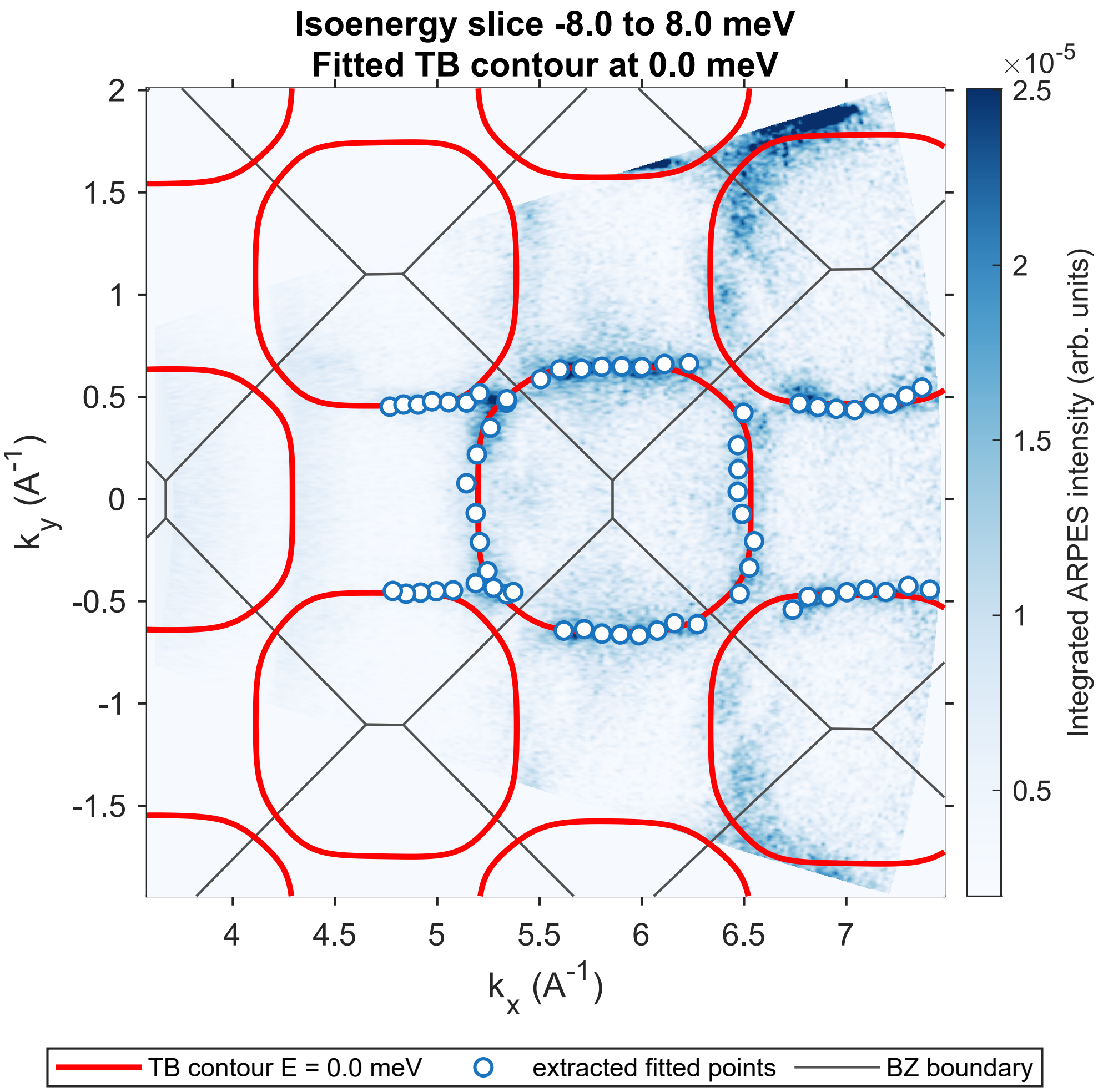

SUPPLEMENTARY FIG. 6. **Tight-binding fit to the experimental Fermi surface.** ARPES intensity integrated over the Fermi-level window -8 meV $< E <$ 8 meV, shown with the extracted experimental Fermi-surface points and the selected tight-binding Fermi contour.Red lines show the $E_{\rm TB} = 0$ contour of the selected tight-binding solution, and gray lines indicate the projected Brillouin-zone boundaries.

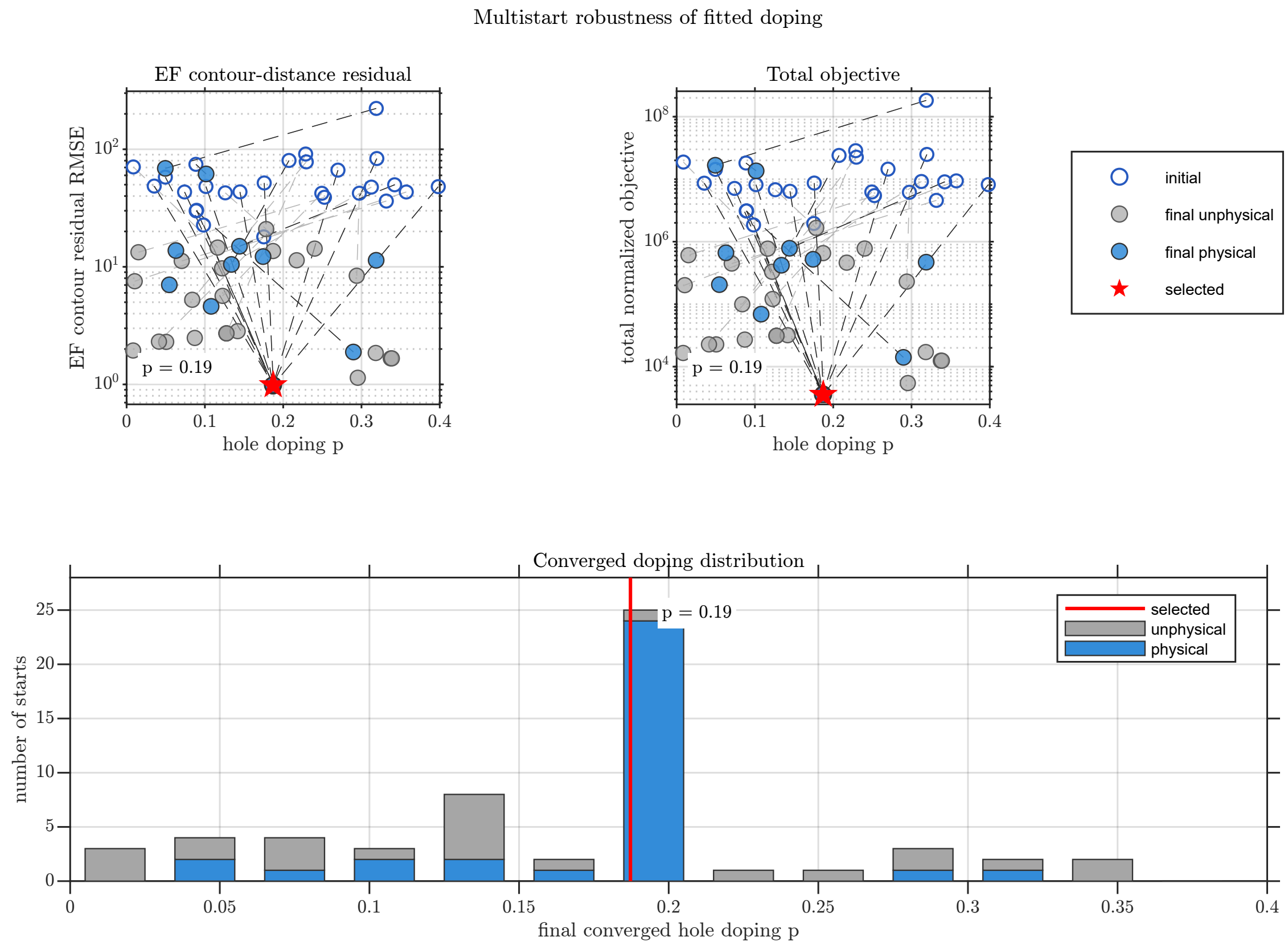

SUPPLEMENTARY FIG. 7. **Multistart robustness of the fitted doping.** The upper panels show the Fermi-level contour-distance residual and the total normalized objective as functions of the hole doping obtained from the converged multistart fits. Open symbols denote initial guesses, gray filled symbols denote converged solutions rejected by the physical-topology criteria, blue filled symbols denote physical converged solutions, and the red star marks the selected solution. The lower panel shows the distribution of converged hole dopings. The dominant physical basin is centered near $p \simeq 0.19$, consistent with the Luttinger count of the selected tight-binding Fermi surface.

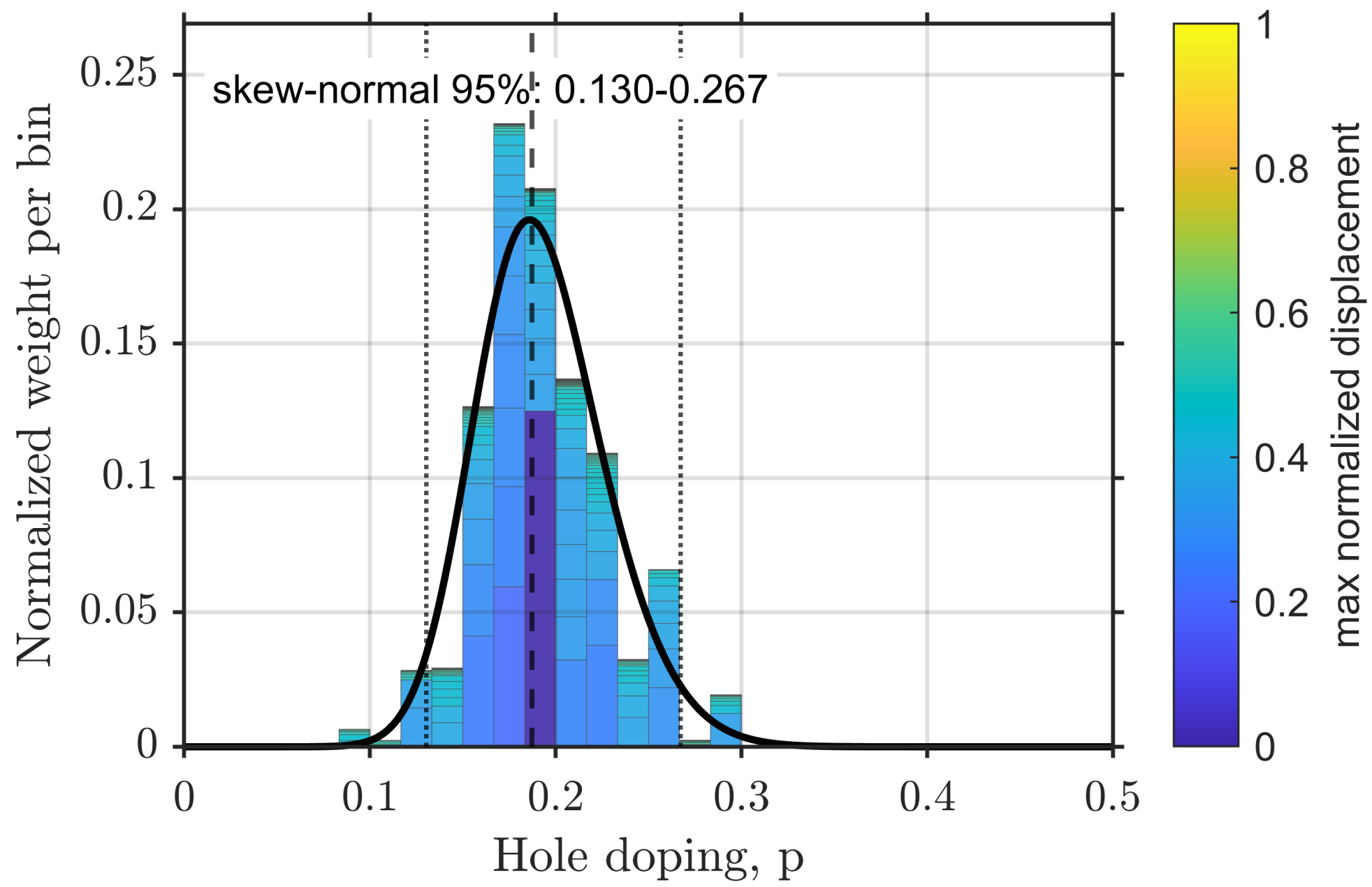

SUPPLEMENTARY FIG. 8. **Weighted parameter-ensemble estimate of the uncertainty in the Luttinger count.** Distribution of hole dopings obtained by perturbing the parameters of the selected tight-binding solution and retaining the resulting models with a continuous compatibility weight. Each block in the histogram columns corresponds to one candidate tight-binding solution; its horizontal position gives the intrinsic Luttinger doping p, while its height is proportional to the normalized compatibility weight of that solution. The color encodes the maximum normalized model displacement from the selected fit, measured in units of the experimental uncertainty. The vertical dashed line marks the doping obtained from the selected multistart solution. The black curve is a skew-normal fit to the weighted distribution, and the dotted vertical lines indicate the corresponding 2.5th and 97.5th percentiles. This analysis gives a 95% interval of approximately $0.130 < p < 0.267$, corresponding to $p = 0.19^{+0.08}_{-0.06}$.

G. EDC integration ranges

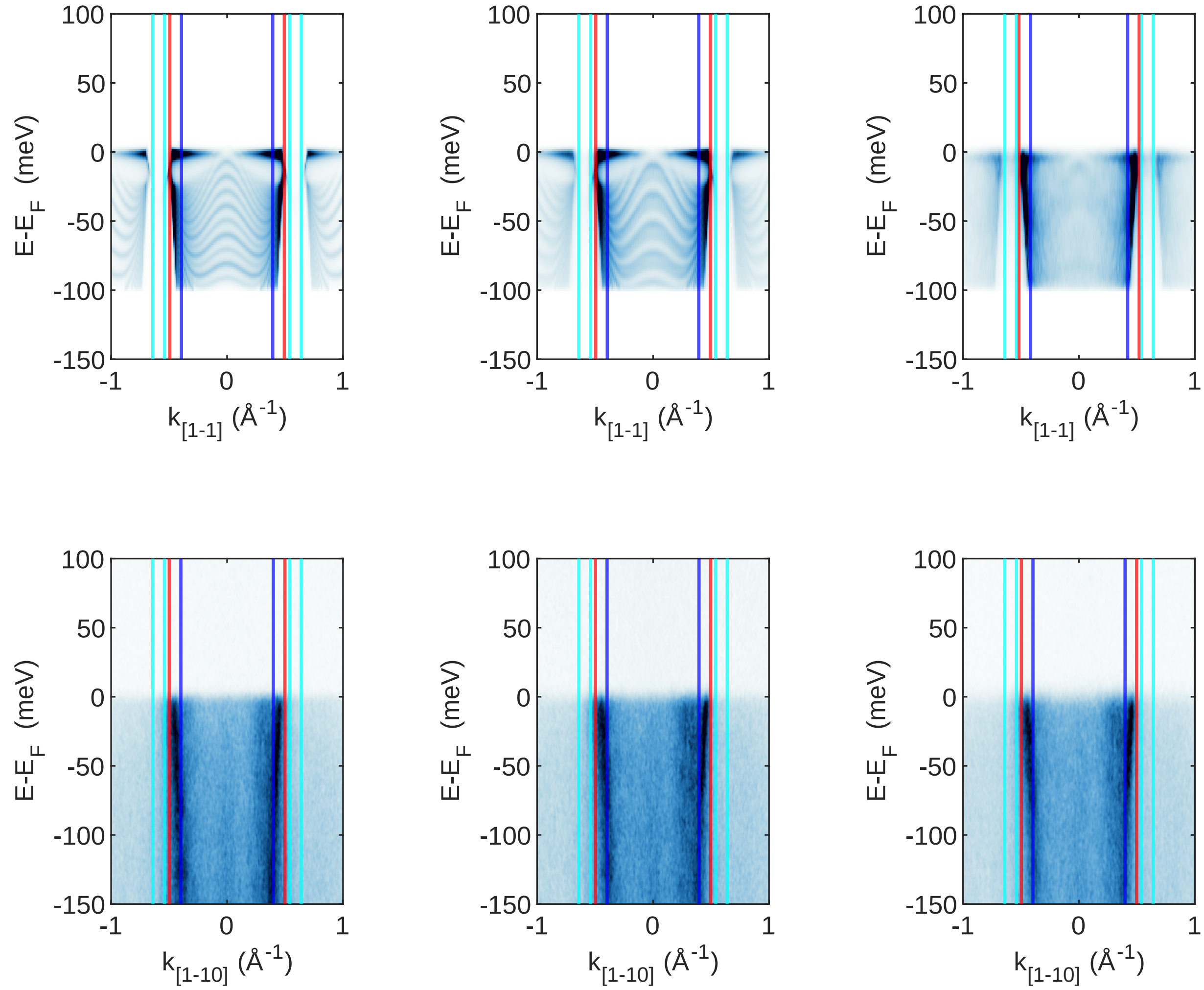

SUPPLEMENTARY FIG. 9. Illustration of the integration ranges for the various EDCs. The region between the cyan and red lines corresponds to the trivial sector ($\nu = 0$), the region between the red and blue line corresponds to the antinode while the region between the blue bands corresponds to the topological sector ($\nu = \pm 1$).

H. Additional EDCs

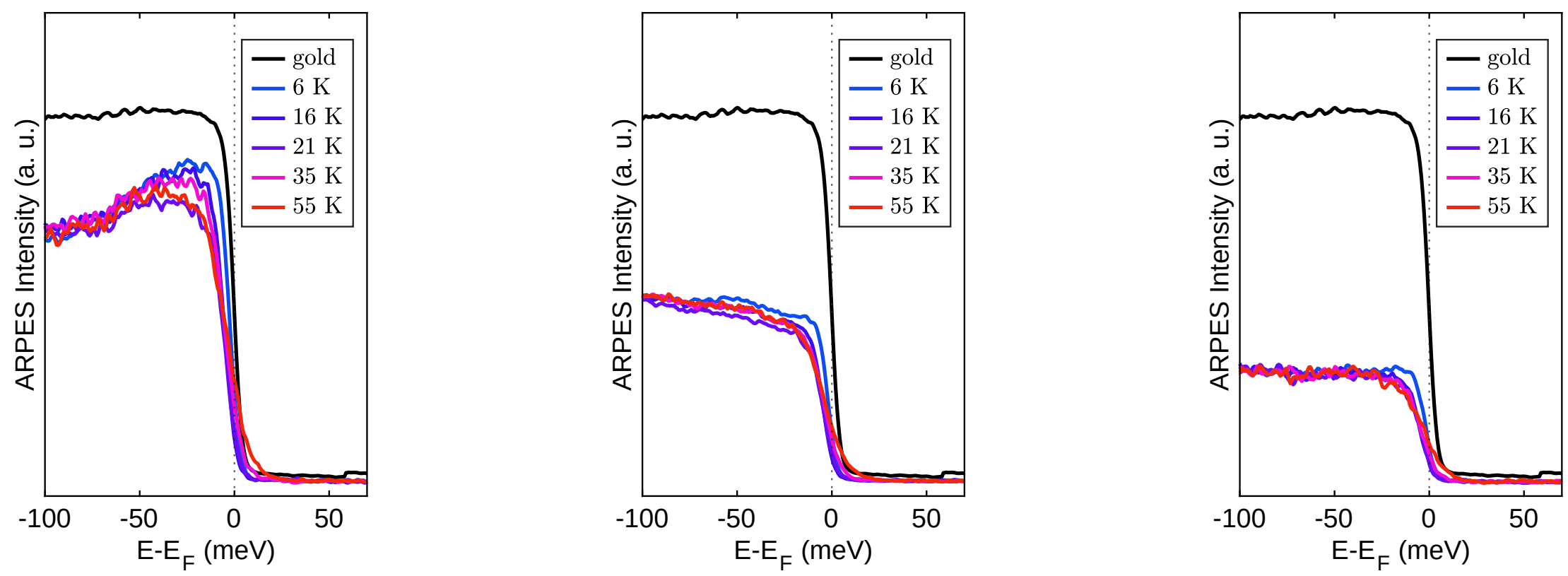

SUPPLEMENTARY FIG. 10. EDCs obtained the same way as in Fig. 4e,g for a different sample.

I. Spatially resolved density of states

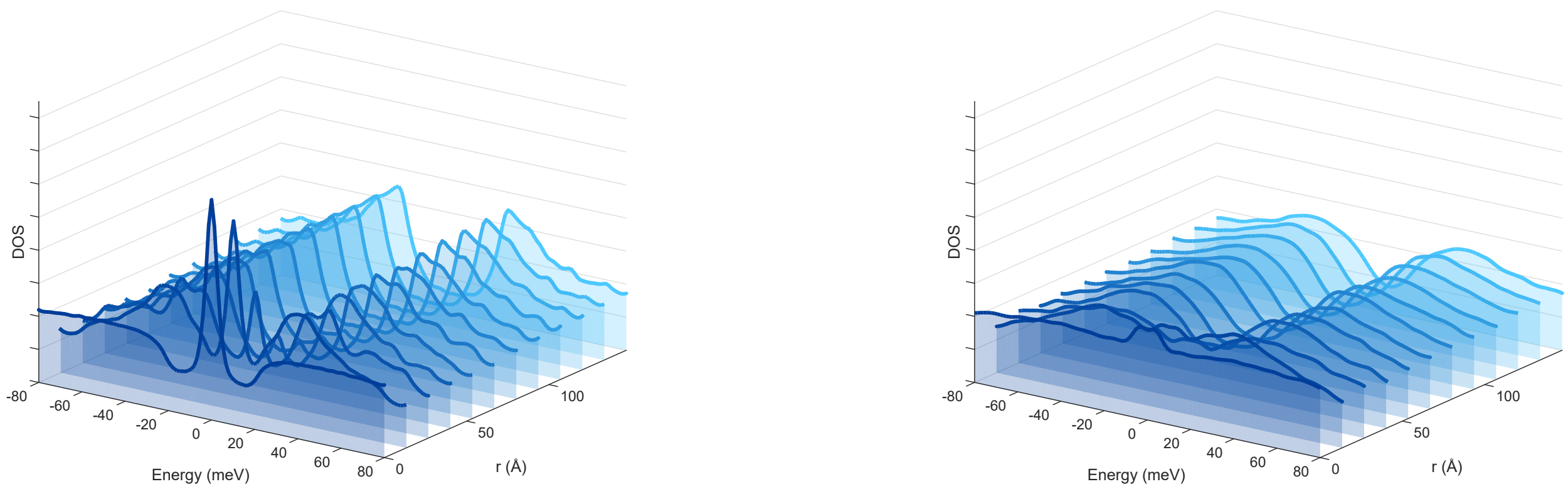

SUPPLEMENTARY FIG. 11. Spatially-resolved density of states for the clean (left) and the GE+BA disordered (right) sample, showing that one can have a bulk gap even for disorder magnitudes that lift the degeneracy of the surface states.

J. Effect of the gap suppression on the edge states

At a pair-breaking (110) boundary of a $d_{x^2-y^2}$-wave superconductor, the local superconducting order parameter is expected to be suppressed near the surface. However, this order-parameter suppression does not translate directly into the opening or closing of a conventional spectral gap in the side-surface ARPES spectral function. In the clean limit, the low-energy edge spectral function is dominated by zero-energy Andreev bound states. As a result, the edge spectral function remains qualitatively similar whether the pairing field is determined self-consistently and suppressed near the surface, or whether a constant bulk-like pairing amplitude is imposed everywhere, up to the edge. Figure 12 compares these two cases. The main effect of self-consistent order-parameter suppression is a modest redistribution of spectral weight and a slight change in the spatial extent of the edge state, rather than the appearance of a conventional superconducting gap feature. This is why the absence of a resolvable gap-like feature in the experimental side-surface spectra should not be interpreted as a direct measurement of the local order parameter. Instead, it is consistent with the expected pair-breaking character of the (110) boundary and with the dominance of low-energy edge spectral weight in the clean theoretical limit.

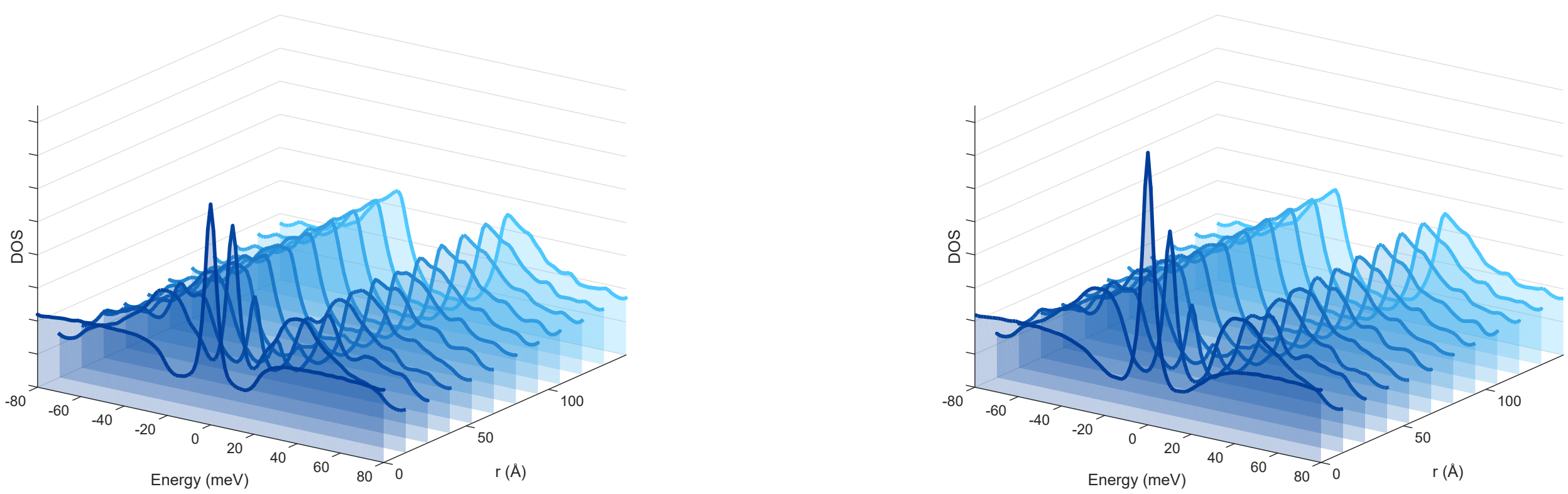

SUPPLEMENTARY FIG. 12. Spatially-resolved density of states for a clean system with gap suppression at the edge (left) and without self consistency of the gap equation, i.e. the gap at the edge is equal to the gap in the bulk (right). The effect of the gap suppression on the spectral function is to have an edge state slightly more delocalized into the bulk as well as narrowing the "gap-like" dip at the very edge.

K. Effect of a thin metallic layer

A possible consequence of a strong surface doping reconstruction could be the formation of a thin metallic layer at the surface, proximity-coupled to the underlying d-wave superconducting bulk. To test whether such a scenario could by itself explain the absence of the flat-band peak in the experiment, we simulated a three-atomic-layer-thick metallic region attached to a [11] edge of a generic d-wave superconductor. The metallic layer does not simply remove the low-energy boundary spectral weight. Instead, confinement within the thin metallic region produces additional quantum-well states at energies comparable to the superconducting gap. These features are not observed in the experimental spectra. Thus, even if a thin metallic surface layer were present, it would not by itself provide a natural explanation for the disappearance of the flat-band-related spectral structure. An additional broadening mechanism, such as disorder, would still be required to suppress these low-energy boundary features to the level observed experimentally.

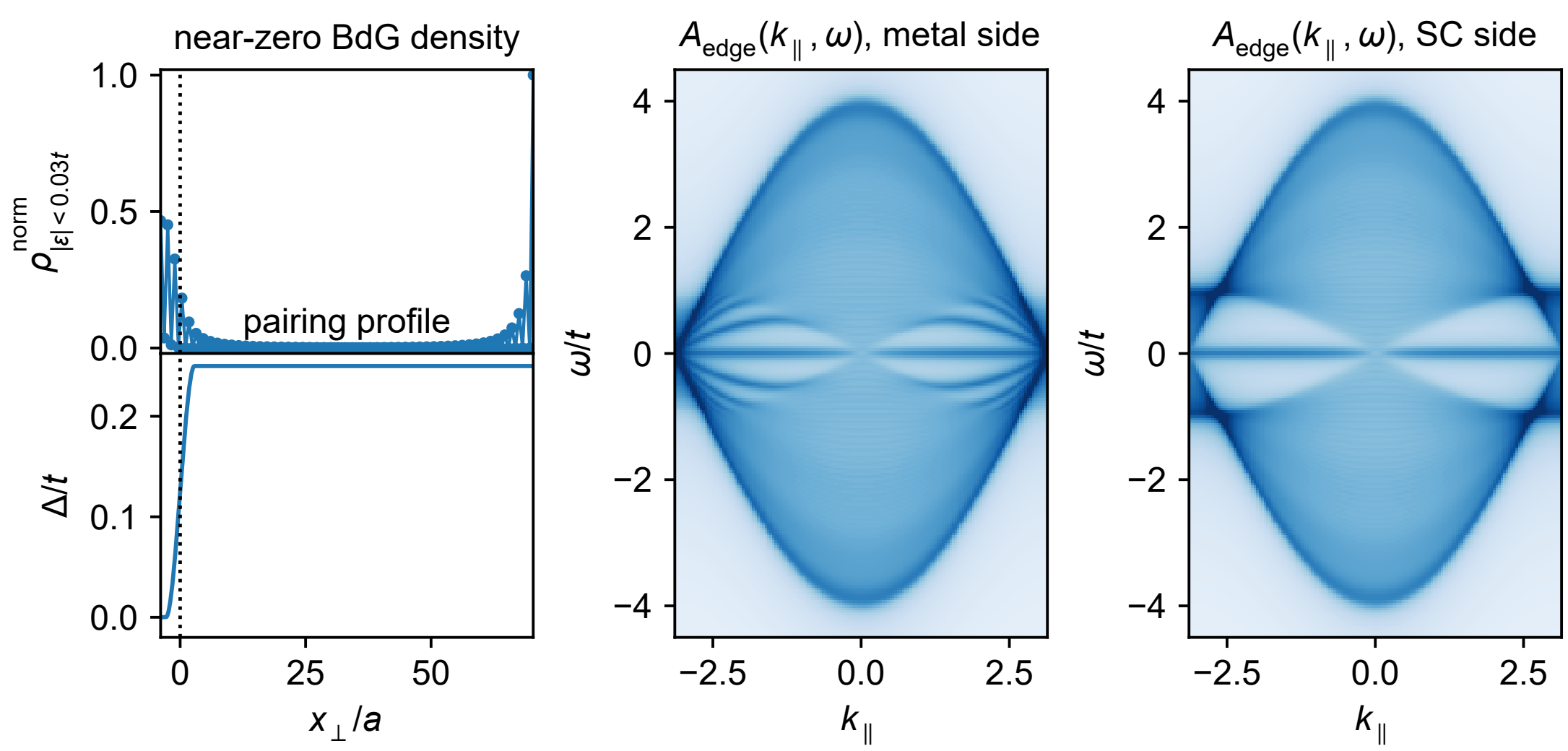

SUPPLEMENTARY FIG. 13. Momentum-integrated, spatially resolved low-energy density of states for a three-atomic-layer-thick metallic layer proximitized by a [11] edge of a d-wave superconductor (left panel). The metallic layer gives rise to quantum-well states in the left-edge spectral function (central panel), which are absent for a clean superconducting edge (right panel).

L. AFM topography

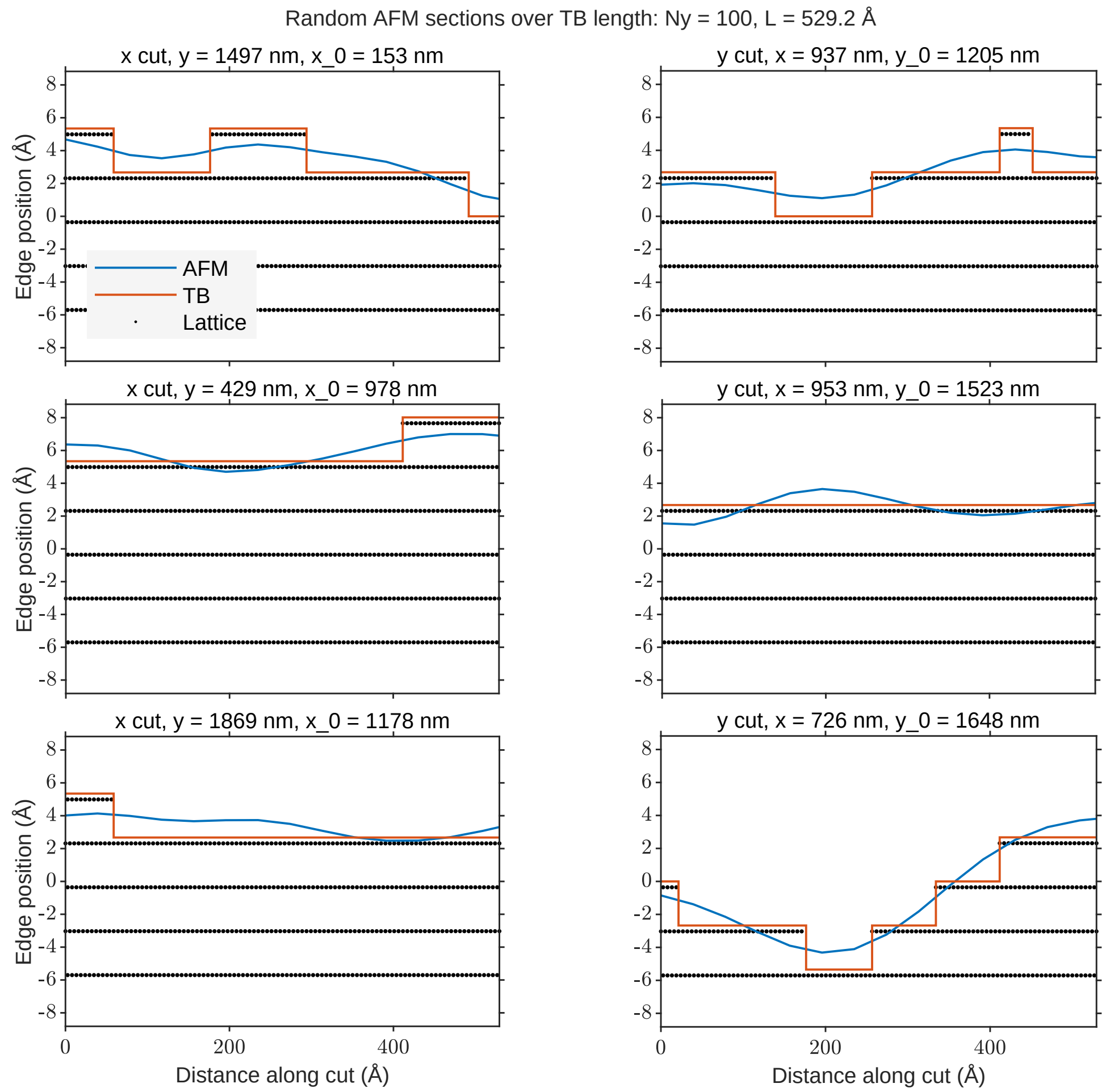

SUPPLEMENTARY FIG. 14. Representative AFM-derived edge profiles used to construct the corresponding tight-binding edge geometries. Each panel shows a line cut extracted from the AFM topography, converted to an edge displacement in Å, together with the discretized tight-binding edge profile and the retained lattice sites. The first panel shows the AFM section used for the calculations presented in the main text. The labels "x cut" and "y cut" denote line cuts taken along the fast-scan and slow-scan directions of the AFM image, respectively. For each cut, the listed x_0 and y_0 denotes the distance of the plotted segment from the edge of the AFM data.

To assess the representativeness of the AFM-derived edge profile used in the main-text calculation, we randomly sampled line cuts from the AFM topography with the same physical length as the simulated tight-binding edge. For each line cut we computed the RMS roughness R_q and an autocorrelation length ξ. The AFM section used in the main-text calculation lies within the region bounded by the 10th and 90th percentiles of the random-

section distribution, indicating that it is representative of the measured surface morphology rather than an outlier.

Within this representative set, the chosen section has a relatively short autocorrelation length. This choice is conservative for the purpose of estimating the effect of AFM-resolvable roughness on the flat-band surface state, since shorter lateral correlation lengths enhance large-momentum scattering and are expected to broaden the surface state more efficiently than smoother profiles with the same RMS roughness. Therefore, the persistence of the flat-band peak for this AFM-derived profile places an upper bound on the broadening attributable to the experimentally resolved surface morphology.

We emphasize that this bound is limited by the lateral resolution of the AFM measurement. Roughness components with wavelengths below the AFM lateral-resolution limit, in particular unit-cell-scale height variations, are not contained in the measured profile. When the AFM profile is mapped onto the lattice model it therefore produces comparatively extended flat segments relative to the atomic length scale. The separate probabilistic $GE_{0.5}$ model is used in the main text to approximate this opposite, high-spatial-frequency limit.

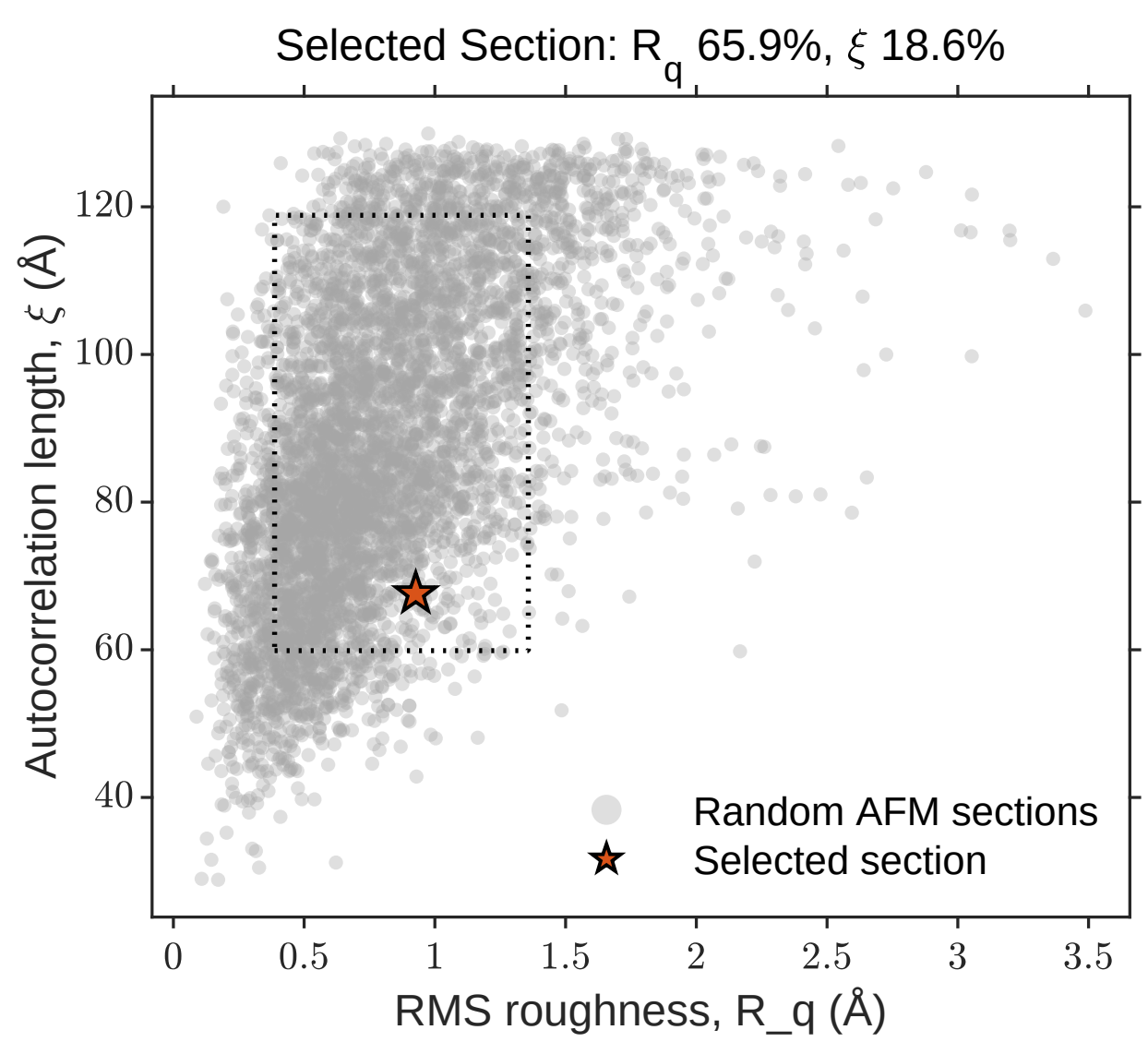

SUPPLEMENTARY FIG. 15. Representativeness analysis of the fixed AFM section used in the first panel of the previous Figure. Gray points show randomly sampled AFM sections of the same physical length as the tight-binding model. Each section is characterized by its RMS roughness, R_q, and autocorrelation length, ξ, computed from the AFM-derived edge profile. The highlighted marker denotes the arbitrary section used in our calculations. The dotted rectangle indicates the region bounded by the 10th and 90th percentiles of the random-section distribution.

M. Different disorder strengths and realizations

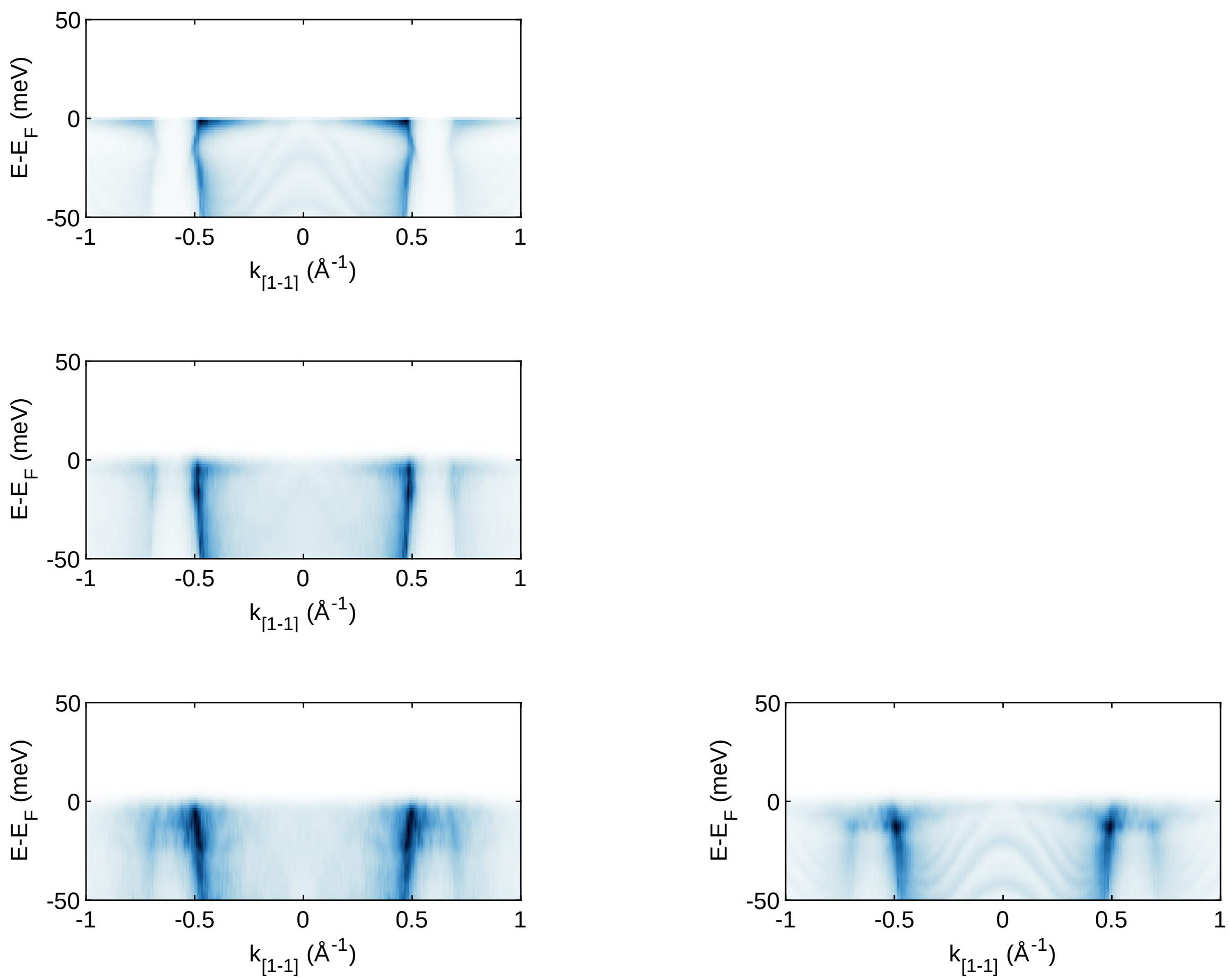

SUPPLEMENTARY FIG. 16. The same spectral function shown in the main text, now evaluated for different disorder strengths. From top to bottom, the first three panels correspond to disorder strengths of 20 meV, 50 meV, and 100 meV. The bottom-right panel also corresponds to 100 meV disorder, but with the disorder potential applied only to the 6 atomic layers closest to the edge.

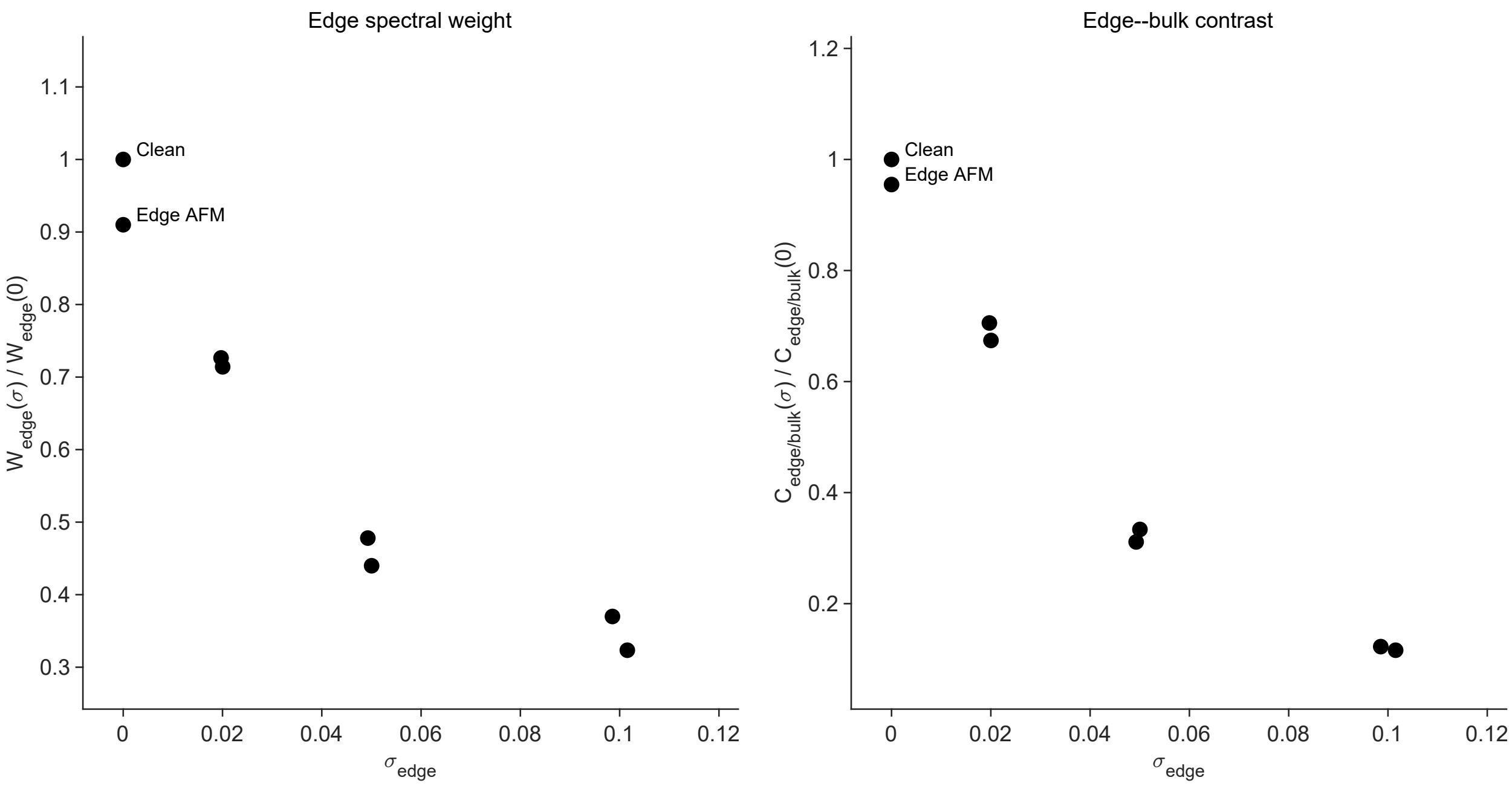

SUPPLEMENTARY FIG. 17. Disorder dependence of the zero-energy flat-band spectral weight at the edge. The horizontal axis in both panels is the standard deviation of the edge disorder, σ_{edge}, computed directly from the disorder profile used in each simulation. The spectral weight is integrated over a low-energy window $E \in [-2.5, 2.5]\,\mathrm{meV}$. Left: normalized integrated edge spectral weight, $W_{\mathrm{edge}}(\sigma)/W_{\mathrm{edge}}(0)$, where $W_{\mathrm{edge}}(\sigma) = \int_{-2.5\,\mathrm{meV}}^{2.5\,\mathrm{meV}} \mathrm{DOS}_{\mathrm{edge}}(E,\sigma)\,dE$. Right: normalized edge–bulk contrast, $C_{\mathrm{edge/bulk}}(\sigma)/C_{\mathrm{edge/bulk}}(0)$, where $C_{\mathrm{edge/bulk}} = W_{\mathrm{edge}}/W_{\mathrm{bulk}}$. The bulk spectral weight is evaluated at the center of the slab using the same number of layers as used for the edge region. All quantities are normalized to the clean zero-disorder reference.

N. Minority s-wave component

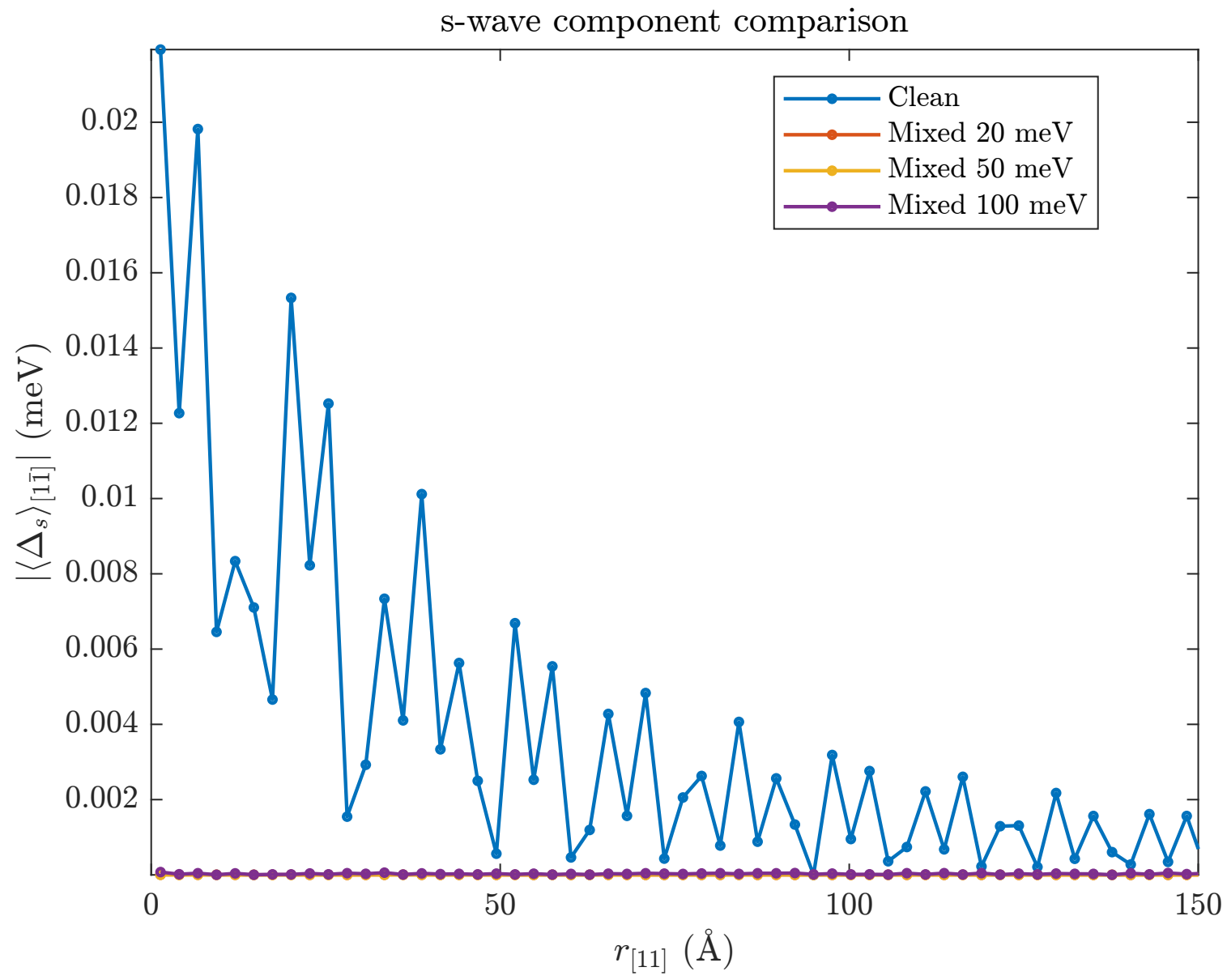

SUPPLEMENTARY FIG. 18. Spatial profile of the bond-resolved imaginary s-wave superconducting component for the clean system and disorder strengths of 20, 50, and 100 meV. This component is very sensitive to onsite disorder (because of its small associated order parameter) and effectively vanishes already for a 20 meV disorder.

O. Shallow core-level photoemission spectra

To assess the surface composition and its evolution during the measurement, we acquired photoemission spectra over the valence-band and shallow core-level energy ranges. The survey spectrum in Fig. 19a contains the expected shallow core levels of $La_{2-x}Sr_xCuO_4$. Possible impurity and adsorbate reference levels are indicated in red. Since shallow levels such as C $2s$ and N $2s$ depend strongly on the local chemical environment, they are represented by Gaussian envelopes rather than by unique binding energies. These envelopes therefore indicate plausible energy ranges but do not constitute direct elemental assignments. This chemical variability, together with the overlap of several candidate contributions, is consistent with the absence of a distinct peak that can be unambiguously assigned to an adsorbate. We therefore characterize the gradual surface degradation through the spectral changes near the valence band, where the strongest evolution is observed. Fig. 19b compares spectra measured shortly after cleavage and after $\Delta t = 18$ h 56 min. By contrast, the remaining features at larger binding energies are mostly unchanged. The intensity of the approximately 3 eV feature increases with time, whereas the weaker feature near 12 eV decreases in relative intensity. The reference binding energies were obtained from the NIST X-ray Photoelectron Spectroscopy Database [7, 8].

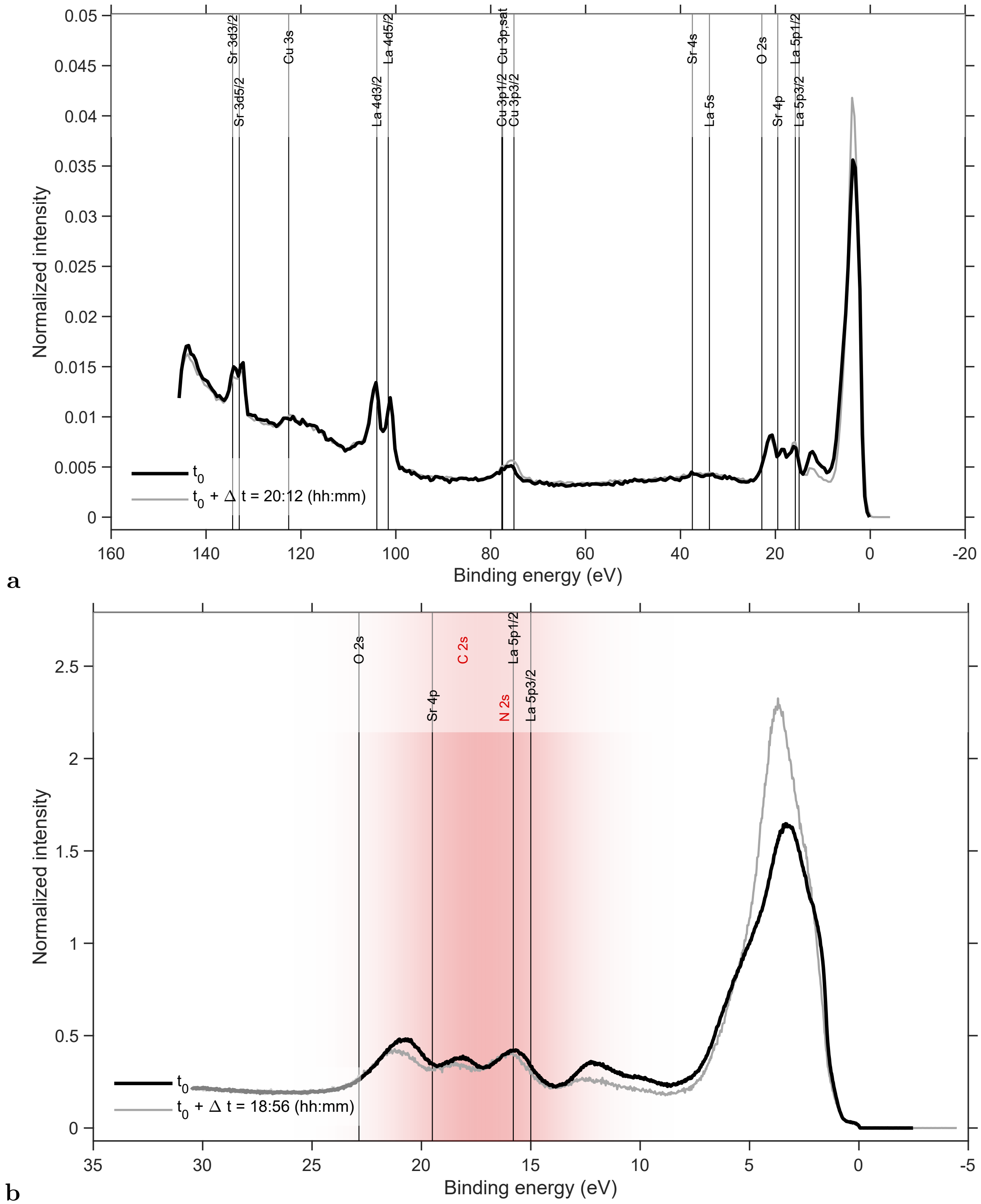

SUPPLEMENTARY FIG. 19. **Shallow core-level and low-binding-energy photoemission spectra.** **a**) Survey spectrum measured at the beginning of the experiment and after Δt = 20 h 12 min with the expected LSCO shallow core-level binding energies indicated by black reference lines. **b**) Low-binding-energy spectra measured at the beginning of the experiment and after Δt = 18 h 56 min. Red lines and shaded envelopes indicate reference energies and chemically dependent energy ranges associated with possible impurities or adsorbates. Reference data were obtained from the NIST X-ray Photoelectron Spectroscopy Database [7, 8].

To quantify this evolution, the features near 3 eV and 12 eV were integrated after subtraction of their local backgrounds. Their peak positions were obtained from the maxima of the background-subtracted spectra using local quadratic interpolation. The resulting time dependences are shown in Fig. 20. The dominant changes in both spectral weight and peak position extend over approximately the first 10–15 h, after which the evolution appears to mostly saturate. The finite evolution time is plausible for a gradual accumulation of adsorbates, with substantial surface coverage being reached only several hours after cleavage. However, a quantitative relation between adsorbate coverage and either flat-band broadening or superconducting-gap suppression cannot be established from these spectra alone. Such a relation would require microscopic knowledge of the adsorbate species, their bonding to the surface, and their spatial distribution. Nevertheless, the observed timescale suggests that any adsorbate-induced modification of the low-energy electronic structure would develop progressively during the typical post-cleavage measurement period, rather than producing an immediate detrimental effect directly after cleavage. For this reason adsorbates are unlikely to be the leading mechanism explaining our ARPES measurements.

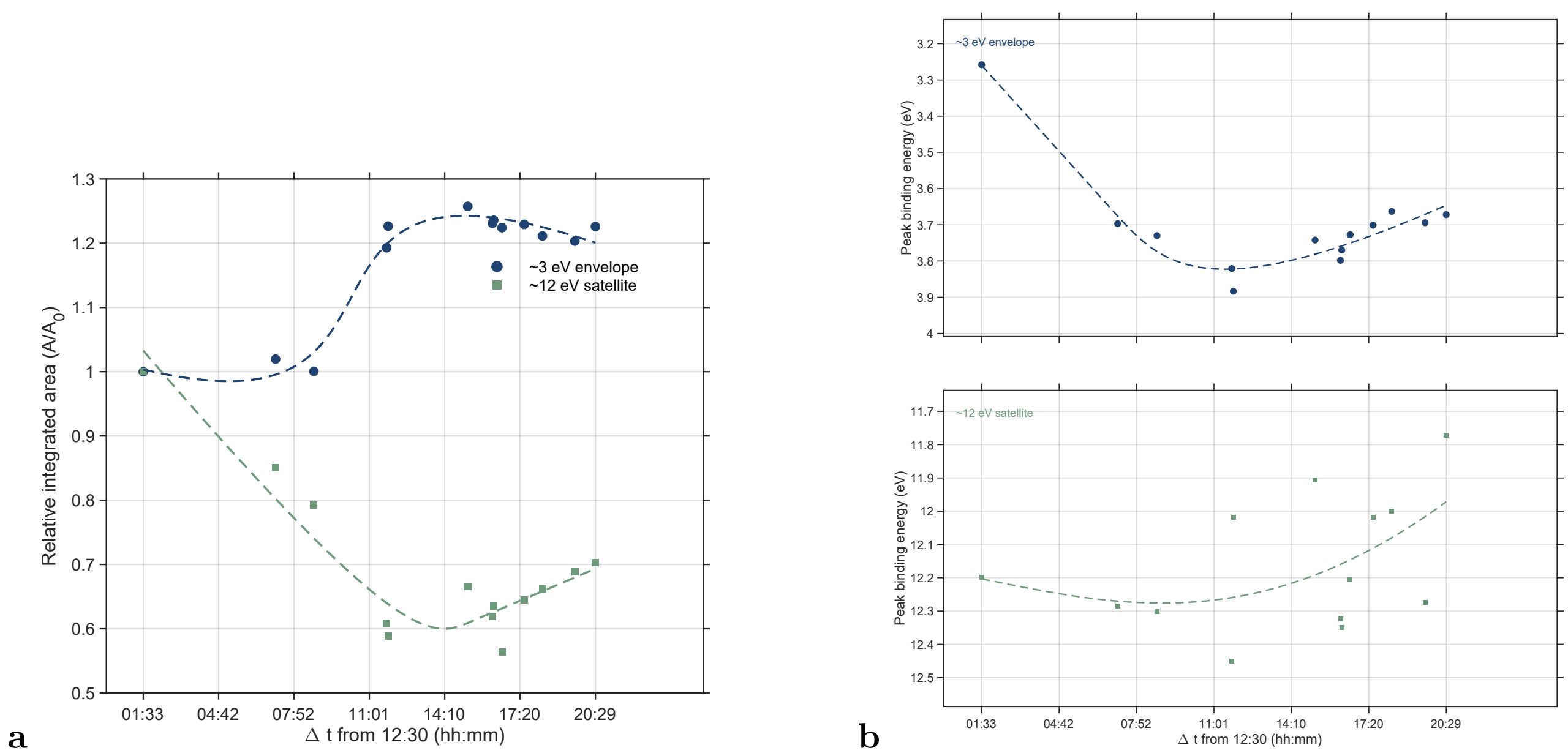

SUPPLEMENTARY FIG. 20. **Time evolution of the low-binding-energy spectral features.** **a**) Integrated areas of the approximately 3 eV envelope and 12 eV satellite, normalized to their first measured values. **b**) Corresponding peak binding energies, with the 3 eV envelope shown in the upper panel and the 12 eV satellite in the lower panel. Dashed curves are smooth guides to the eye.

[1] S. Ryu and Y. Hatsugai, Physical Review Letters **89** (2002), 10.1103/physrevlett.89.077002.

[2] M. Sato, Y. Tanaka, K. Yada, and T. Yokoyama, Physical Review B **83** (2011), 10.1103/physrevb.83.224511.

[3] Y. Zhong, Z. Chen, S.-D. Chen, K.-J. Xu, M. Hashimoto, Y. He, S.-i. Uchida, D. Lu, S.-K. Mo, and Z.-X. Shen, Proceedings of the National Academy of Sciences **119** (2022), 10.1073/pnas.2204630119.

[4] T. Yoshida, W. Malaeb, S. Ideta, D. H. Lu, R. G. Moor, Z.-X. Shen, M. Okawa, T. Kiss, K. Ishizaka, S. Shin, S. Komiya, Y. Ando, H. Eisaki, S. Uchida, and A. Fujimori, Physical Review B **93** (2016), 10.1103/physrevb.93.014513.

[5] R. S. Markiewicz, S. Sahrakorpi, M. Lindroos, H. Lin, and A. Bansil, Physical Review B **72** (2005), 10.1103/physrevb.72.054519.

[6] T. Yoshida, X. J. Zhou, D. H. Lu, S. Komiya, Y. Ando, H. Eisaki, T. Kakeshita, S. Uchida, Z. Hussain, Z.-X. Shen, and A. Fujimori, Journal of Physics: Condensed Matter **19**, 125209 (2007).

[7] Justin Gorham, en"Nist x-ray photoelectron spectroscopy database - srd 20," (2012).

[8] A. Y. Lee, C. J. Powell, J. M. Gorham, A. Morey, J. H. J. Scott, and R. J. Hanisch, Data Science Journal **23**, 45 (2024).
