# Supplementary material for: Suppression of spectral gap and flat bands on a cuprate superconductor side-surface
Source: Nat Commun. 2026 Oct 7;17:10573. doi: 10.1038/s41467-026-78020-y (PMC13645798; doi:10.1038/s41467-026-78020-y)
Supplement: Supplementary file 2 [file 41467_2026_78020_MOESM2_ESM.pdf]

nature portfolio

Peer Review File

Suppression of Spectral Gap and Flat Bands on a Cuprate Superconductor Side-Surface

Corresponding Author: Dr Niels Schröter

This file contains all reviewer reports in order by version, followed by all author rebuttals in order by version.

Version 0:

Reviewer comments:

Reviewer #1

(Remarks to the Author)
This manuscript reports angle-resolved photoemission spectroscopy (ARPES) measurements on the (110) side surface of overdoped LSCO, enabled by FIB-assisted sample preparation. This approach allows, for the first time, momentum-resolved access to the electronic structure of cuprate side surfaces, which have been historically inaccessible due to cleavage limitations. The authors observe a suppression of the superconducting spectral gap at the side surface and, notably, the absence of the expected zero-energy flat-band states. Through Bogoliubov–de Gennes simulations incorporating geometric roughness and bulk disorder, they argue that moderate Anderson-type disorder can strongly broaden and suppress the surface flat bands. The manuscript is clearly written, and the experimental data are of generally high quality. Given that this work represents the first realization of side-surface ARPES in cuprates, it constitutes a significant technical and conceptual advance.

While I find the study highly interesting and potentially suitable for publication, I believe that several technical issues should be clarified before the manuscript can be accepted.

First, although the surface is reported to be atomically flat, it remains unclear whether the carrier concentration at the surface is quantitatively identical to that of the bulk. The absence of a superconducting gap in the ARPES spectra could in principle arise from a deviation in surface doping rather than intrinsic pair-breaking effects. In Fig. 3b, the experimentally determined Fermi surface does not appear to quantitatively match the simulated one in terms of enclosed volume. While the manuscript mentions an estimate of the surface doping based on the Fermi surface area, a quantitative and transparent analysis is not presented. I therefore strongly encourage the authors to extract the Fermi surface volume directly from the experimental data and demonstrate explicitly that the surface doping level is consistent with the bulk value.

Second, surface flatness does not necessarily guarantee surface cleanliness, which is particularly critical for measurements performed at low temperature (6 K). It is important to establish that the (110) surface remains sufficiently clean and free from adsorbates during the measurement. In this regard, additional evidence such as shallow core-level spectroscopy would be helpful. Furthermore, a discussion of possible surface aging effects would strengthen confidence in the reliability of the spectra.

Third, I would like to raise a concern regarding the role of strong kz broadening inherent to the present measurement geometry. The manuscript estimates a kz broadening on the order of ~0.4 ¥AA-1, which implies that the measured spectra may contain contributions from a wide range of momenta, including near-nodal regions where the superconducting gap is small or vanishing. Such mixing could effectively mask the superconducting gap even at nominally antinodal momenta. It would therefore be important to clarify whether the absence of a gap can be unambiguously attributed to pair-breaking or disorder effects, rather than being influenced by momentum broadening. In addition, it would be useful to clarify whether this effect is fully captured in the simulations, and to what extent it may affect the visibility of the superconducting gap.

In summary, this work presents a highly significant advance by enabling side-surface ARPES measurements on cuprate superconductors for the first time. The manuscript is well written and supported by high-quality data and simulations. I would be happy to recommend publication after the above concerns are adequately addressed.

Reviewer #2

(Remarks to the Author)
This manuscript aims to investigate the zero-energy topological surface flat band on the side surface of a cuprate superconductor which has long been predicted theoretically but has not been directly observed by ARPES. The authors use a FIB-based preparation method to expose the otherwise inaccessible (110) surface and perform momentum-resolved ARPES measurements. Despite this technically demanding experimental approach the expected flat-band peak is not observed. The authors interpret this absence as a consequence of bulk disorder which broadens the surface-state spectral weight and suppresses the ARPES-detectable peak.

First I would like to emphasize that this work represents a highly notable attempt to overcome a long-standing limitation of ARPES namely that the accessible cleaving surface is usually fixed by the crystal structure. Previous STM and tunneling experiments have reported zero-bias peaks which have often been regarded as important evidence for topological surface flat bands. However the lack of momentum resolution in these probes makes it difficult to distinguish topological surface states from disorder-induced or interface-induced in-gap states. In this respect the authors' FIB-based sample preparation and subsequent ARPES measurement on a cuprate side surface constitute a challenging and important experimental advance. This approach opens a route to examine surface electronic structures in momentum space that were previously difficult to access. Given the surface characterization presented by the authors the absence of the expected topological surface flat band is itself an important result that highlights a possible gap between theoretical expectations and experimental reality. The attempt to explain this result in terms of surface roughness and disorder is also a reasonable first line of investigation.

The authors further examine this scenario using real-space BdG simulations. In particular they solve a self-consistent BdG gap equation to analyze how surface roughness and disorder affect the pairing field and the surface spectral function. Such calculations require repeated reconstruction and diagonalization of the BdG Hamiltonian at each iteration and therefore demand substantial computational resources as well as careful treatment of convergence and numerical stability. In this sense the calculations go beyond a purely qualitative argument and represent a meaningful microscopic attempt to examine order-parameter suppression at the surface and disorder-induced broadening of the surface state. Nevertheless there remain several issues in the computational setup and presentation. These limitations raise the question of whether the disorder-based interpretation can be sufficiently generalized from the calculations currently presented.

The manuscript also emphasizes in the Introduction that the topological surface flat band on a cuprate side surface is highly susceptible to various symmetry-breaking instabilities because of its large degeneracy and high density of states. The authors discuss several possible competing surface orders including time-reversal-symmetry-breaking states, mixed-parity superconductivity, phase-crystal states and magnetic order. They also mention several alternative scenarios in connection with previous ZBCP observations such as interface states disorder-induced bound states and facet mixtures. However when interpreting the absence of the flat-band peak in the present experiment the manuscript does not sufficiently discuss how these scenarios may be compatible with or constrained by the present result. The absence of the peak is mainly explained using BdG calculations with surface roughness and bulk disorder while the possible effects of surface instabilities, time-reversal symmetry breaking, phase-crystal formation or edge magnetism on the flat-band spectral weight and ARPES visibility are only limitedly discussed. In addition although interface states and facet mixtures may be partly constrained by the surface characterization and the momentum-resolved nature of ARPES the possible influence of microscopic domain or facet structures on the flat-band spectral weight could be discussed more explicitly. Such a discussion would not weaken the authors' interpretation. Rather it would clarify the relative position of possible explanations and improve the balance and persuasiveness of the manuscript. Therefore the generality and conclusiveness of the present interpretation would benefit from a more balanced discussion of alternative scenarios and a clearer statement of the limitations of the calculations. Since the central interpretation relies substantially on real-space BdG simulations the following points should be considered.

1. A more systematic examination of disorder strength is needed. The manuscript introduces Anderson disorder as a Gaussian onsite mass term and mainly presents results for a standard deviation of 100meV. This value is considerably larger than the superconducting gap scale. Of course considering that impurity potentials in cuprates can reach several tens to several hundreds of meV the choice of 100meV is not necessarily unrealistic. However if the absence of the flat-band peak is to be attributed to disorder-induced broadening it would be useful to show how the flat-band peak the bulk superconducting gap and the edge spectral weight evolve as the disorder strength is varied. For example results for several representative disorder strengths such as 20meV 50meV and 100meV would help clarify whether the suppression of the flat-band peak depends sensitively on a particular disorder scale or is robust over a broader range.

2. The representativeness of the disorder model requires further discussion. The onsite disorder used in the manuscript is a reasonable minimal model for demonstrating that bulk inhomogeneity in cuprates can broaden the flat-band peak. In particular site-to-site Gaussian disorder efficiently introduces short-length-scale scattering and is therefore a suitable starting point for testing whether the flat-band surface state can be broadened by disorder. However disorder in real cuprates may arise from dopants, oxygen vacancies, extended impurity potentials and spatially correlated disorder. These microscopic profiles can directly affect the strength of large-momentum-transfer scattering and the degree of surface-state broadening. For instance spatially smooth or extended disorder may produce weaker scattering between opposite chirality sectors than short-range onsite disorder and may therefore suppress the flat-band peak less strongly than in the present calculation. Although the authors acknowledge in the Discussion that disorder can take various forms this discussion is not sufficiently connected to the representativeness of the specific disorder model used in the simulations. The authors do not necessarily need to compare all possible disorder types computationally. However a more explicit qualitative discussion is needed regarding how well the chosen disorder model represents the disorder landscape of LSCO and whether this choice may overestimate the suppression of the flat-band peak. Since the key conclusion of the manuscript relies strongly on disorder-

induced broadening clarifying the physical meaning and limitations of the chosen disorder model is important.

3. It is unclear whether disorder realizations were averaged. Anderson disorder can produce different local spectral weights and different degrees of flat-band broadening depending on the random configuration. Therefore an ensemble average over several configurations would be desirable. Although some degree of self-averaging may be expected in a sufficiently large system the flat-band surface state considered here can be sensitive to spatial inhomogeneity near the edge and to the details of boundary segments. Thus realization dependence should not be assumed to be negligible a priori. The manuscript does not specify how many disorder configurations were used whether the results were averaged or how large the variation between realizations is. It would therefore be helpful to clarify whether the presented spectra correspond to a representative realization or to an ensemble-averaged result.

4. The statistical character of surface roughness is not sufficiently analyzed. The manuscript incorporates the AFM-measured roughness profile through edge-site removal and concludes that the experimentally resolvable roughness is insufficient to remove the flat-band peak. This is a meaningful approach because it directly uses the measured surface information. However the influence of surface roughness cannot be fully assessed from the RMS roughness alone. Even for the same roughness amplitude a long lateral correlation length can preserve clean edge segments and allow the flat band to survive whereas unit-cell-scale high-frequency roughness can enhance large-momentum scattering and broaden the surface state more efficiently. The authors mention that high in-plane spatial frequency roughness may be important but the spectral function is not systematically examined as a function of roughness autocorrelation length or high-frequency components. Thus the conclusion that the AFM-derived roughness is insufficient to suppress the flat band is convincing within the measured roughness profile but it does not fully characterize the broader effect of surface roughness disorder.

5. The effects of edge disorder and bulk disorder are not sufficiently separated. Since the flat-band surface state is localized near the boundary its response can depend strongly on whether disorder is located at the edge layer within the near-surface region of the coherence length or throughout the bulk. The manuscript does account for the finite probing depth of ARPES through exponential weighting and kz broadening which appropriately reflects the surface sensitivity of the measurement. However the spectral response is not systematically separated according to whether the disorder is edge-localized near-surface-limited or bulk-distributed. Since ARPES is a surface-sensitive probe it would be useful to compare how edge-local onsite disorder and bulk-distributed disorder differently affect the flat-band peak and how bulk disorder is projected onto the surface spectral weight. Although the manuscript compares geometric roughness with bulk Anderson disorder the relative contributions of surface disorder and bulk inhomogeneity to the suppression of the flat-band peak remain in need of further clarification.

6. The numerical implementation of the dx2−y2 pairing symmetry should be described more clearly. The manuscript states that a nearest-neighbor pairing field is determined self-consistently but the way in which the dx2−y2 symmetry is implemented in the actual calculation is not described in sufficient detail. For example it is unclear whether the relative sign between the x-bond and y-bond pairing amplitudes is imposed only through the initial condition whether the d-wave symmetry is explicitly constrained during the self-consistency loop or how freely the bond-resolved pairing field is allowed to vary in the presence of disorder and boundaries. Since the central analysis relies on surface order-parameter suppression and the resulting changes in the flat-band spectral function it is important to clarify how the symmetry structure of the pairing field is maintained and interpreted during the calculation. Providing this information would improve the reproducibility and physical transparency of the self-consistent BdG simulations.

In summary this manuscript presents a technically challenging and meaningful attempt to measure the cuprate side surface using momentum-resolved ARPES. The FIB-based preparation of an otherwise inaccessible crystallographic surface and the direct measurement of its electronic structure are clear strengths of the work. The self-consistent BdG calculations also provide an important framework for examining how surface roughness and disorder affect flat-band visibility. However the central interpretation that the absence of the flat-band peak can be explained by bulk-disorder-induced broadening is still not sufficiently established by the present calculations and discussion. In particular the robustness of the disorder-based scenario the representativeness of the chosen disorder model the role of competing surface-order scenarios and the details of the self-consistent BdG implementation require further clarification. Therefore while I find the experimental approach valuable and potentially important I do not think the manuscript is ready for publication in its present form. I would recommend publication only after major revision addressing the issues raised above.

Version 1:

Reviewer comments:

Reviewer #1

(Remarks to the Author)
I have carefully read the revised manuscript and the authors' responses to my previous comments. The authors have made substantial efforts to improve the manuscript and have satisfactorily addressed the concerns raised in my previous report. In particular, the authors have provided a more quantitative analysis of the surface carrier concentration based on the experimentally determined Fermi surface, added further discussion and experimental evidence regarding surface cleanliness and possible aging effects, and clarified the influence of kz broadening on the observed spectra and its treatment in the simulations. I find the authors' responses to these points convincing and the corresponding revisions appropriate.

Overall, I believe that the manuscript has been significantly improved and that my previous concerns have been adequately addressed. I therefore recommend publication in its present form.

Reviewer #2

(Remarks to the Author)
The authors have responded carefully and constructively to my previous comments. In particular, the revised manuscript now includes a disorder-strength dependence, additional disorder realizations, a statistical analysis of the AFM-derived roughness, a comparison with near-surface-localized disorder, a clearer description of the self-consistent BdG implementation, and a more balanced discussion of alternative surface-instability scenarios. These additions substantially improve the robustness and transparency of the analysis. Given the significant experimental advance represented by the first momentum-resolved ARPES study of a cuprate side surface, I am satisfied that the manuscript is now suitable for publication in Nature Communications.
I retain a modest concern regarding the strength of the causal language used for the disorder interpretation. The calculations convincingly demonstrate that short-range Anderson-type disorder provides a sufficient and physically plausible mechanism for suppressing the flat-band peak, but they do not uniquely establish that disorder is the key microscopic factor in the measured LSCO surface. This limitation is appropriately acknowledged in the rebuttal and Discussion, although the final sentence of the Abstract remains somewhat stronger than the evidence warrants. I encourage the authors to consider a modest softening of this wording, but I do not regard this issue as an obstacle to publication.

Open Access This Peer Review File is licensed under a Creative Commons Attribution 4.0 International License, which permits use, sharing, adaptation, distribution and reproduction in any medium or format, as long as you give appropriate credit to the original author(s) and the source, provide a link to the Creative Commons license, and indicate if changes were made.
In cases where reviewers are anonymous, credit should be given to 'Anonymous Referee' and the source.
The images or other third party material in this Peer Review File are included in the article's Creative Commons license, unless indicated otherwise in a credit line to the material. If material is not included in the article's Creative Commons license and your intended use is not permitted by statutory regulation or exceeds the permitted use, you will need to obtain permission directly from the copyright holder.
To view a copy of this license, visit https://creativecommons.org/licenses/by/4.0/

Response to Reviewers

Manuscript: *Suppression of Spectral Gap and Flat Bands on a Cuprate Superconductor Side-Surface*

Response to Reviewer #1

Comment 1: This manuscript reports angle-resolved photoemission spectroscopy (ARPES) measurements on the (110) side surface of overdoped LSCO, enabled by FIB-assisted sample preparation. This approach allows, for the first time, momentum-resolved access to the electronic structure of cuprate side surfaces, which have been historically inaccessible due to cleavage limitations. The authors observe a suppression of the superconducting spectral gap at the side surface and, notably, the absence of the expected zero-energy flat-band states. Through Bogoliubov–de Gennes simulations incorporating geometric roughness and bulk disorder, they argue that moderate Anderson-type disorder can strongly broaden and suppress the surface flat bands. The manuscript is clearly written, and the experimental data are of generally high quality. Given that this work represents the first realization of side-surface ARPES in cuprates, it constitutes a significant technical and conceptual advance.

While I find the study highly interesting and potentially suitable for publication, I believe that several technical issues should be clarified before the manuscript can be accepted.

Response to comment 1:

We thank the reviewer for considering our work a significant technical and conceptual advance, and for identifying points that required clearer presentation. We have addressed the three specific concerns below.

Comment 2: First, although the surface is reported to be atomically flat, it remains unclear whether the carrier concentration at the surface is quantitatively identical to that of the bulk. The absence of a superconducting gap in the ARPES spectra could in principle arise from a deviation in surface doping rather than intrinsic pair-breaking effects. In Fig. 3b, the experimentally determined Fermi surface does not appear to quantitatively match the simulated one in terms of enclosed volume. While the manuscript mentions an estimate of the surface doping based on the Fermi surface area, a quantitative and transparent analysis is not presented. I therefore strongly encourage the authors to extract the Fermi surface volume directly from the experimental data and demonstrate explicitly that the surface doping level is consistent with the bulk value.

Response to comment 2:

We thank the reviewer for raising this important point. We agree that the carrier concentration of the measured side surface must be addressed explicitly, since a strong surface doping reconstruction could in principle affect the interpretation of the low-energy spectra. However, unlike the well-known surface electronic reconstruction in YBCO(001), which is associated with its polar cleaved surface and termination-dependent charge redistribution, LSCO surfaces are not expected to exhibit an analogous polarity-driven self-doping. In particular, for the relevant LSCO(001) and (110) terminations, no large deviation between the near-surface and bulk hole concentration is expected from analogous electrostatic polarity considerations. We have therefore substantially expanded the Supplementary section describing the Luttinger-count analysis of the experimentally measured Fermi surface.

We also clarify in the main text that the simulated Fermi surface shown in Fig. 3 is not the one used to determine the surface doping. That contour was generated from the one-band tight-binding parametrization of LSCO by Markiewicz et al., using the same dressed hopping parameters and varying only the chemical potential until the nodal Fermi momentum matched the experimental value. It is

therefore intended as a visual guide to the measured momentum-space structure and as a comparison to a standard literature parametrization. It is not expected to reproduce all details of the measured Fermi-surface intensity due to the simplified one-band nature of the model, photoemission matrix-element effects, residual distortions in the experimental momentum conversion, and the finite k_z broadening inherent to the side-surface photon-energy map.

The surface doping was instead estimated independently from the experimentally extracted Fermi-surface area, using the extended fitting and Luttinger-count procedure now described in more detail in Supplementary Sec. G: Estimation of the doping level. In this analysis, the tight-binding model is fitted directly to the experimental Fermi-surface points, with all the parameters allowed to vary. This procedure gives a surface hole concentration of approximately p=0.19. Given the uncertainty of such a Luttinger-count estimate in LSCO, this value should be interpreted as an estimate of the doping regime rather than as a high-precision determination of the absolute surface carrier density. In particular, deviations between Luttinger-count estimates and nominal Sr concentration have already been reported for conventional LSCO(001) ARPES measurements e.g. in the work by Yoshida et al., and in the present side-surface geometry, finite k_z-broadening provides an additional source of uncertainty. Within these uncertainties, the estimated surface doping is compatible with the nominal p=0.22 composition of our sample (which as a reminder should correspond to a superconducting gap size of approximately 15-20 meV[1]) and remains confidently inside the superconducting region of LSCO. To estimate the confidence of this result, we perturbed the selected tight-binding solution within the experimental energy and momentum resolution giving a weighted doping distribution centered near p=0.19, with a 95% interval of approximately p=0.130–0.267, which spans the superconducting regime of LSCO (a more detailed explanation of the methodology is included in the Supplementary Section G: Estimation of the doping level). This conclusion is also consistent with our magnetic-susceptibility measurements, which confirm bulk superconductivity after the FIB preparation procedure. We therefore do not find evidence that the absence of a resolved superconducting gap or flat-band peak can be explained by a surface doping reconstruction that moves the measured surface outside the superconducting regime.

Finally, even a hypothetical much stronger reconstruction would not naturally account for our observations. If the surface were reconstructed into a metallic overdoped layer proximity-coupled to the underlying d-wave superconductor, the Andreev bound states would not be removed a priori; instead, one would expect additional low-energy quantized states on the scale of the superconducting gap (see the Supplementary section L: effects of a thin metallic layer). Conversely, if the surface were reconstructed into an insulating underdoped state, one would not expect to observe the well-defined Fermi surface that we measure. Thus, while we cannot exclude small quantitative deviations between the surface and bulk carrier concentrations, a doping reconstruction does not provide a natural explanation for the missing flat-band peak. This reinforces the conclusion of the manuscript that disorder-induced broadening is the most plausible mechanism.

Revision to the manuscript

Fermi Surface Characterization:

[1] Y. Zhong, Z. Chen, S. Chen, K. Xu, M. Hashimoto, Y. He, S. Uchida, D. Lu, S. Mo, & Z. Shen, Differentiated roles of Lifshitz transition on thermodynamics and superconductivity in La2-xSrxCuO4, Proc. Natl. Acad. Sci. U.S.A. 119 (32) e2204630119, https://doi.org/10.1073/pnas.2204630119 (2022).

The tight-binding contour shown for comparison in Fig. 3b was generated from the one-band LSCO parametrization of Ref. [62]. In this comparison, the dressed hopping parameters were kept fixed to the literature values and only the chemical potential was rigidly shifted so as to reproduce the value of k_F measured experimentally in the nodal cut. This calculation is therefore intended as a visual guide to the experimental momentum-space structure and small discrepancies between the measured intensity and this idealized contour are expected due to the simplified one-band nature of the model, photoemission matrix-element effects, residual distortions of the experimental momentum conversion, and the finite k_z broadening inherent to the side-surface photon-energy map. The effective doping of the measured surface was instead estimated from the experimentally extracted Fermi-surface area using the Luttinger-count procedure described in Supplementary Sec. VI G, giving a surface hole doping of approximately $p \simeq 0.19$. This value is compatible with the nominal bulk doping $p = 0.22$ when accounting for the small known discrepancies between Luttinger-count estimates and nominal Sr concentration reported also in conventional LSCO(001) ARPES measurements [63], for which we also note that LEED measurements did not find evidence for a surface reconstruction [64]. Thus, within the accuracy of the analysis, the measured side surface remains in the overdoped superconducting regime and does not indicate a significant surface reconstruction of the type reported in some other cuprates [65].

Broadening of Surface States and gap frustration by pair breaking:

signature for identifying the surface states irrespective of the gap suppression. Indeed, this local suppression is not prominently reflected in the edge spectrum, because the low-energy edge spectral function is dominated by zero-energy Andreev bound states. Consistently, our calculations show that including a self-consistent suppression of the order parameter near the edge produces negligible changes in the low-energy edge spectral function (Supplementary Sec. VI M).

Nevertheless, although bulk superconductivity has been confirmed in our samples by magnetic susceptibility measurements (Fig. 2b), and the surface doping level is consistent with the bulk value (see Supplementary section VI I), our spectra at k_z values corresponding to the antinodes show no clear dip–peak structure at the Fermi level within the experimental energy resolution of 3–4 meV (see Fig. 4g and the corresponding integration ranges in Supplementary section VI J). Even accounting for the uncertainty in the estimated surface doping, moderate deviations from the expected doping would only negligibly shift the nodal coordinates [70], and thus the momentum extent of the topological flat band, without substantially modifying the spectral function. If the doping change were large enough to generate a gapless metallic layer proximity-coupled to the underlying d-wave superconducting state, one would not expect a trivial broadening of the flat band. Instead, this scenario would produce discrete quantum-well states (Supplementary Sec. VI N), for which we find no evidence in the experimental data. Finally, we do not resolve any temperature-dependent spectral feature that could be associated with the opening or closing of a superconducting gap, which is expected to be approximately 20 meV at our doping level [54]. Although this observation does not by itself prove order-parameter suppression, given its weak influence on the edge spectral function, it is nevertheless consistent with expectations for a pair-breaking boundary dominated by Andreev physics, as discussed above. Notice that the

Methods: Three-dimensional Fermi surface

The simulated three-dimensional Fermi surface of LSCO shown as a main-text visual comparison was generated using the one-band tight-binding parametrization of Ref. [62]. The hopping parameters were kept fixed to the literature values, and the chemical potential was set to $\mu = -393.3$ meV in order to match the experimentally measured nodal Fermi momentum, $k_F = -0.4605$ Å^{-1}. This calculation was used only as a reference contour for visual comparison with the measured ARPES intensity. The surface doping was determined independently from the fitted experimental Fermi-surface area, as described in Supplementary Sec. VI G.

Supplementary: Effects of the gap suppression on the edge states:

At a pair-breaking (110) boundary of a $d_{x^2-y^2}$-wave superconductor, the local superconducting order parameter is expected to be suppressed near the surface. However, this order-parameter suppression does not translate directly into the opening or closing of a conventional spectral gap in the side-surface ARPES spectral function. In the clean limit, the low-energy edge spectral function is dominated by zero-energy Andreev bound states. As a result, the edge spectral function remains qualitatively similar whether the pairing field is determined self-consistently and suppressed near the surface, or whether a constant bulk-like pairing amplitude is imposed everywhere, up to the edge. Figure 13 compares these two cases. The main effect of self-consistent order-parameter suppression is a modest redistribution of spectral weight and a slight change in the spatial extent of the edge state, rather than the appearance of a conventional superconducting gap feature. This is why the absence of a resolvable gap-like feature in the experimental side-surface spectra should not be interpreted as a direct measurement of the local order parameter. Instead, it is consistent with the expected pair-breaking character of the (110) boundary and with the dominance of low-energy edge spectral weight in the clean theoretical limit.

New Supplementary section: Effect of a thin metallic layer:

L. Effect of a thin metallic layer

A possible consequence of a strong surface doping reconstruction could be the formation of a thin metallic layer at the surface, proximity-coupled to the underlying d-wave superconducting bulk. To test whether such a scenario could by itself explain the absence of the flat-band peak in the experiment, we simulated a three-atomic-layer-thick metallic region attached to a [11] edge of a generic d-wave superconductor. The metallic layer does not simply remove the low-energy boundary spectral weight. Instead, confinement within the thin metallic region produces additional quantum-well states at energies comparable to the superconducting gap. These features are not observed in the experimental spectra. Thus, even if a thin metallic surface layer were present, it would not by itself provide a natural explanation for the disappearance of the flat-band-related spectral structure. An additional broadening mechanism, such as disorder, would still be required to suppress these low-energy boundary features to the level observed experimentally.

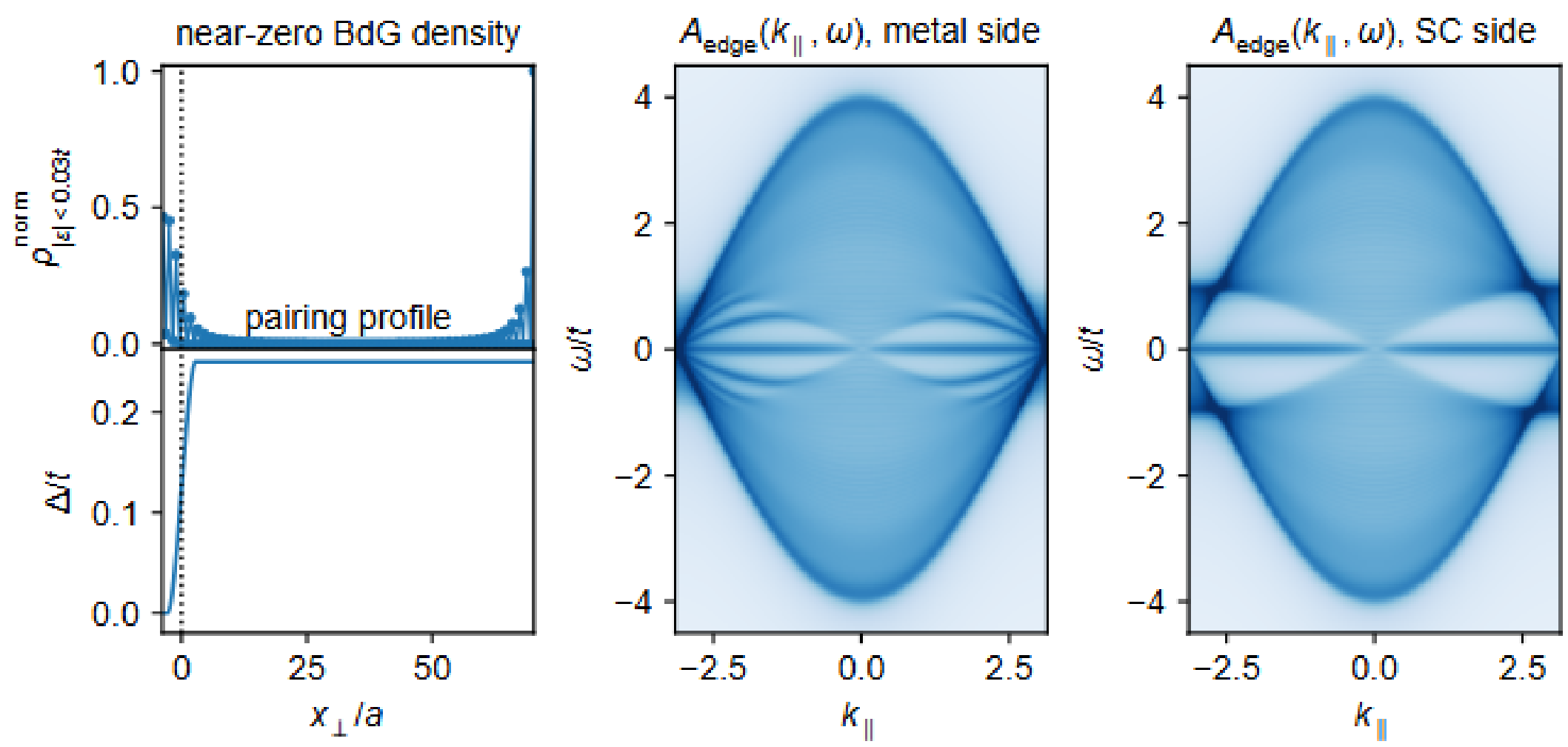

FIG. 16. Momentum-integrated, spatially resolved low-energy density of states for a three-atomic-layer-thick metallic layer proximitized by a [11] edge of a d-wave superconductor (left panel). The metallic layer gives rise to quantum-well states in the left-edge spectral function (central panel), which are absent for a clean superconducting edge (right panel).

Supplementary: Estimation of the doping level:

On top of the two figures below, this new supplementary text also contains a detailed description of the fitting procedure and is highlighted in blue in the manuscript.

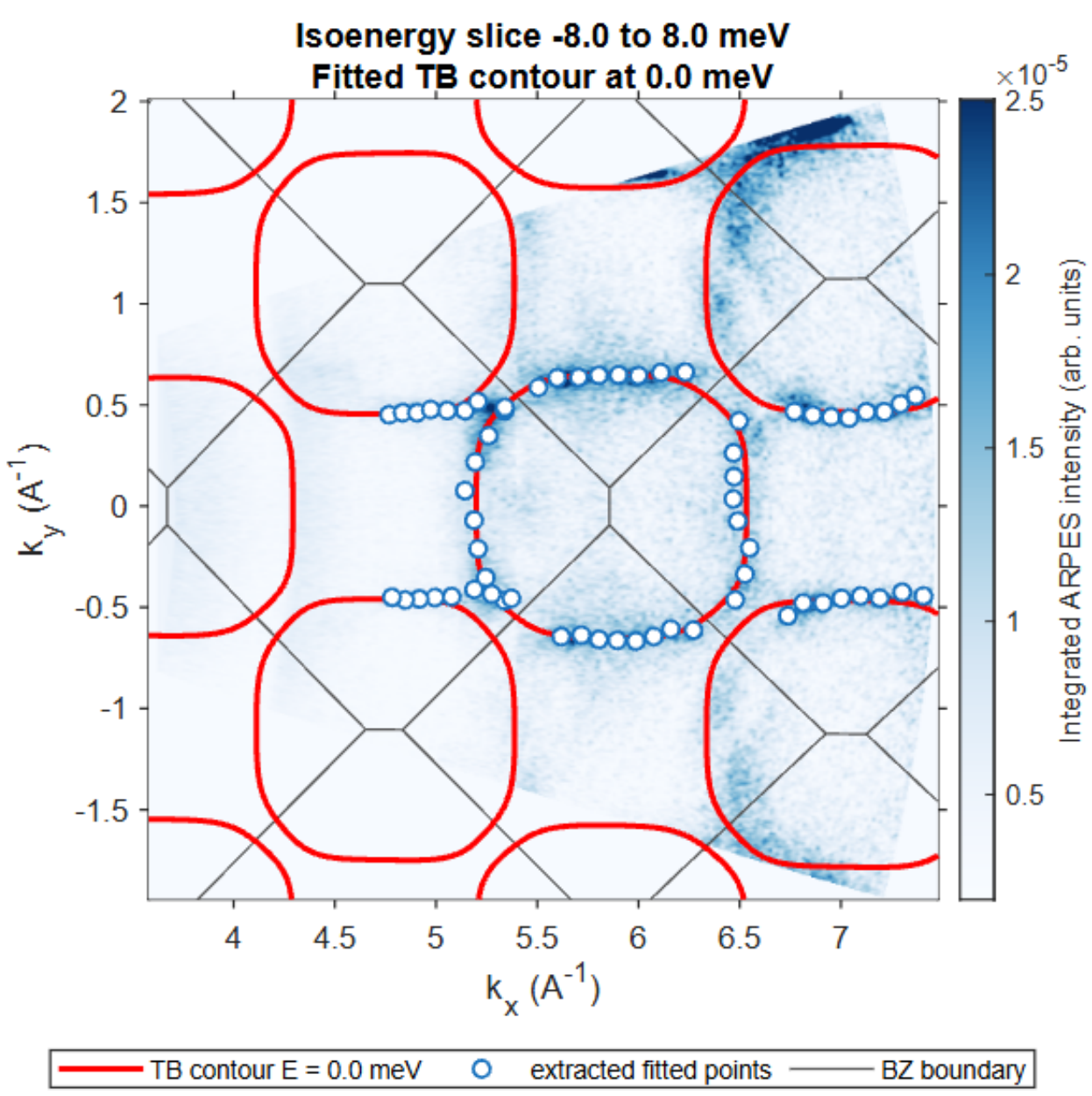

FIG. 10. **Tight-binding fit to the experimental Fermi surface.** ARPES intensity integrated over the Fermi-level window -8 meV $< E <$ 8 meV, shown with the extracted experimental Fermi-surface points and the selected tight-binding Fermi contour. Blue circles mark the fitted points obtained from the radial extraction procedure, red lines show the $E_{\mathrm{TB}} = 0$ contour of the selected tight-binding solution, and gray lines indicate the projected Brillouin-zone boundaries.

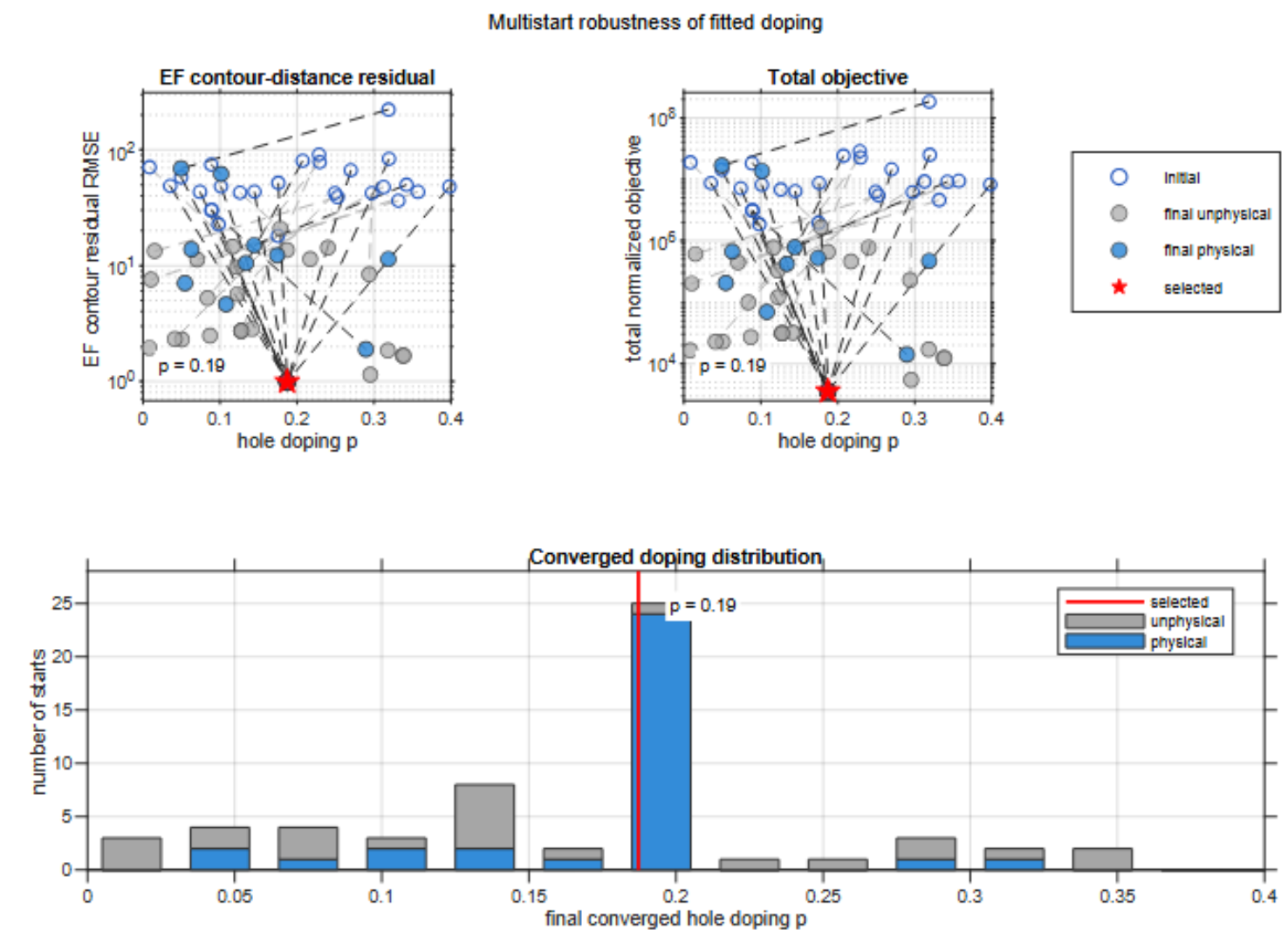

FIG. 11. **Multistart robustness of the fitted doping.** The upper panels show the Fermi-level contour-distance residual and the total normalized objective as functions of the hole doping obtained from the converged multistart fits. Open symbols denote initial guesses, gray filled symbols denote converged solutions rejected by the physical-topology criteria, blue filled symbols denote physical converged solutions, and the red star marks the selected solution. The lower panel shows the distribution of converged hole dopings. The dominant physical basin is centered near $p \simeq 0.19$, consistent with the Luttinger count of the selected tight-binding Fermi surface.

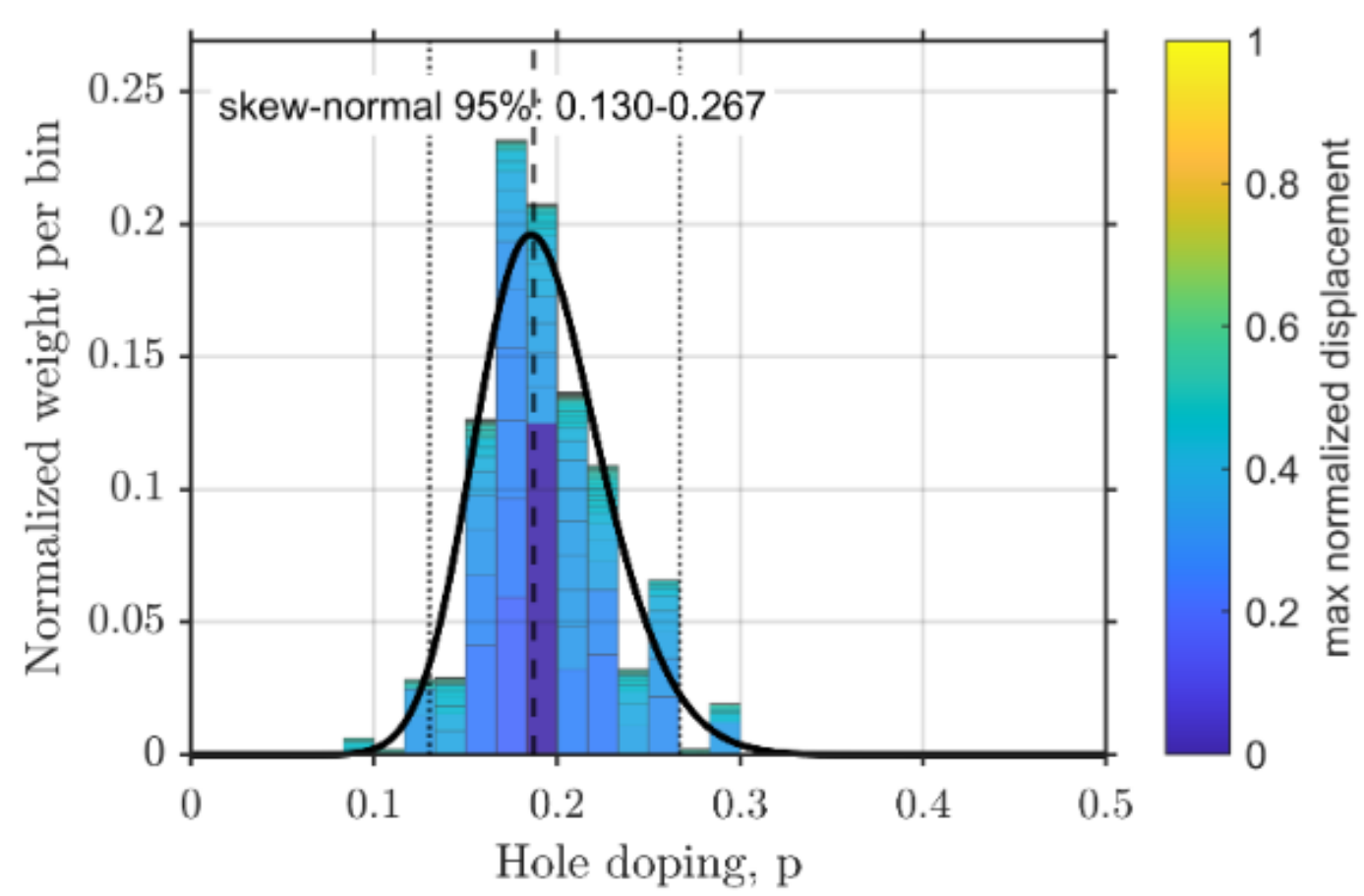

FIG. 12. **Weighted parameter-ensemble estimate of the uncertainty in the Luttinger count.** Distribution of hole dopings obtained by perturbing the parameters of the selected tight-binding solution and retaining the resulting models with a continuous compatibility weight. Each block in the histogram columns corresponds to one candidate tight-binding solution; its horizontal position gives the intrinsic Luttinger doping p, while its height is proportional to the normalized compatibility weight of that solution. The color encodes the maximum normalized model displacement from the selected fit, measured in units of the experimental uncertainty. The vertical dashed line marks the doping obtained from the selected multistart solution. The black curve is a skew-normal fit to the weighted distribution, and the dotted vertical lines indicate the corresponding 2.5th and 97.5th percentiles. This analysis gives a 95% interval of approximately $0.130 < p < 0.267$, corresponding to $p = 0.19^{+0.08}_{-0.06}$.

Comment 3: Second, surface flatness does not necessarily guarantee surface cleanliness, which is particularly critical for measurements performed at low temperature (6 K). It is important to establish that the (110) surface remains sufficiently clean and free from adsorbates during the measurement. In this regard, additional evidence such as shallow core-level spectroscopy would be helpful. Furthermore, a discussion of possible surface aging effects would strengthen confidence in the reliability of the spectra.

Response to comment 3:

We agree with the reviewer that topographic flatness alone does not establish chemical cleanliness.

The side surface was exposed by in-situ mechanical cleaving under ultrahigh vacuum, with a base pressure of approximately $1–2 \times 10^{-10}$ mbar, and the ARPES measurements were performed immediately after cleavage at low temperature. Importantly, no clearly resolved flat-band feature was observed even in the first spectra acquired immediately after cleavage. No nonstandard glue or capping layer was present on the cleaved surface. In addition, the observation of well-defined dispersive features, a recognizable Fermi surface, and the ability to distinguish nodal and antinodal regions provide direct spectroscopic evidence that the measured surface is sufficiently clean for momentum-resolved ARPES. Moreover, the LSCO (001) surface has been studied both by us and other ARPES groups which successfully measured the superconducting gap over the years with cleaving at low temperature[2,3], which because of the resolution requirements and the relatively low photo-emission cross-section of cuprates, require relatively long acquisition times. It is reasonable to assume that the adsorption rate does not differ significantly between the (110) and (001) surface, so that we can safely rule out adsorbates as the direct cause for the absence of a spectral gap on the (110) surface.

This is further supported by the fact that we have performed the measurements by integrating over approximately 10-15 minutes intervals, in order to keep track of possible changes in the data quality over time, and we did not observe any significant change over the acquisition time. Because it is unlikely for the density of adsorbates to be large immediately after cleaving, we believe that the relevant mechanism explaining our results must be at play already immediately after cleaving the sample. Finally, also notice that disorder at the edge actually reduces the order parameter suppression at the edge (see Fig. 4b). This can be understood by realizing that the introduction of disorder reduces the deconstructive interference leading to the gap suppression while effectively introducing different edge orientations that are not pair-breaking (e.g. 100 and 001).

---

[2] Shi, M., Chang, J., Pailhés, S., Norman, M. R., Campuzano, J. C., Mansson, M., Claesson, T., Tjernberg, O., Bendounan, A., Patthey, L., Momono, N., Oda, M., Ido, M., Mudry, C., & Mesot, J. (2008). Coherent d-wave superconducting gap in underdoped La2−xSrxCuO4 by angle-resolved photoemission spectroscopy. Physical Review Letters, 101(4), 047002. https://doi.org/10.1103/PhysRevLett.101.047002

[3] Yoshida, T., Hashimoto, M., Ideta, S., Fujimori, A., Tanaka, K., Mannella, N., Hussain, Z., Shen, Z.-X., Kubota, M., Ono, K., Komiya, S., Ando, Y., Eisaki, H., & Uchida, S. (2009). Universal versus material-dependent two-gap behaviors of the high-Tc cuprate superconductors: Angle-resolved photoemission study of La2−xSrxCuO4. Physical Review Letters, 103(3), 037004. https://doi.org/10.1103/PhysRevLett.103.037004

The detailed analysis of the shallow core-level spectra, together with the Figures, as presented in the Supplementary section VII is shown in the pages below in the Revision to the manuscript section.

Thus, the main result of our work is that a time-independent source of disorder broadens the edge states filling the spectral gap. Adsorbates, residual defects, oxygen disorder, dopant disorder, and other microscopic sources of scattering would all enter this broader category of disorder mechanisms capable of broadening the surface spectral weight, however energy scale and time-dependence considerations suggest that the leading source of disorder are likely chemical and lattice inhomogeneities. Regarding aging, several samples were measured over extended periods of time. Within the experimental reproducibility, we did not observe a systematic time-dependent degradation of the Fermi surface, disappearance of the dispersive bands, or emergence of new low-energy spectral features. To address this point more directly, we have added a new Supplementary section presenting valence-band and shallow core-level photoemission spectra acquired as a function of time after cleavage. The survey spectrum contains the expected LSCO shallow core levels, while no distinct feature can be unambiguously assigned to an adsorbate. Nevertheless, gradual changes are observed in the features near 3eV and 12 eV, with the saturation of the evolution occurring over approximately the first 10–15h after cleavage. This timescale indicates progressive surface aging rather than substantial contamination immediately after cleavage. Since the low-energy ARPES measurements were initiated directly after cleavage and show no corresponding time-dependent reconstruction, the shallow core-level analysis further supports our conclusion that adsorbates are unlikely to be the primary origin of the observed gap filling and suppression of the flat-band peak. We have also added a corresponding discussion and reference to this Supplementary section in the main text.

As an additional check, we performed a simple time-dependent adsorption calculation coupled to a [110]-edge tight-binding model. The residual-gas composition was taken from a measured UHV composition found in the literature, which is H2 dominated with smaller CH4, H2O, CO, and CO2 components[4]. Adsorbates were treated as irreversible at 6 K and modelled as local weak-to-moderate scalar scatterers. For the scattering strengths we used values from cuprate disorder modelling[5]. These values should be regarded as conservative upper-bound estimates for ordinary residual-gas adsorbates, since the reference values describe dopant/native-defect disorder in or near the cuprate planes, which we expect to perturb the low-energy electronic structure more strongly than weakly physisorbed residual-gas molecules. Within this approximation, the time needed to produce a significant additional broadening of the [110] flat band exceeds the few-hour measurement window of our experiment. Despite the simplicity of the model, this supports the conclusion that residual-gas adsorption is unlikely to be the primary origin of the gap filling observed immediately after cleavage.

---

[4] M. Grabski and E. Al-Dmour, "Commissioning and operation status of the MAX IV 3 GeV storage ring vacuum system," *Journal of Synchrotron Radiation* **28**, 718–731 (2021), doi: **10.1107/S1600577521002599**.

[5] T. S. Nunner, B. M. Andersen, A. Melikyan, and P. J. Hirschfeld, "Fourier transform spectroscopy of ddd-wave quasiparticles in the presence of atomic scale pairing disorder," *Physical Review B* **73**, 104511 (2006).

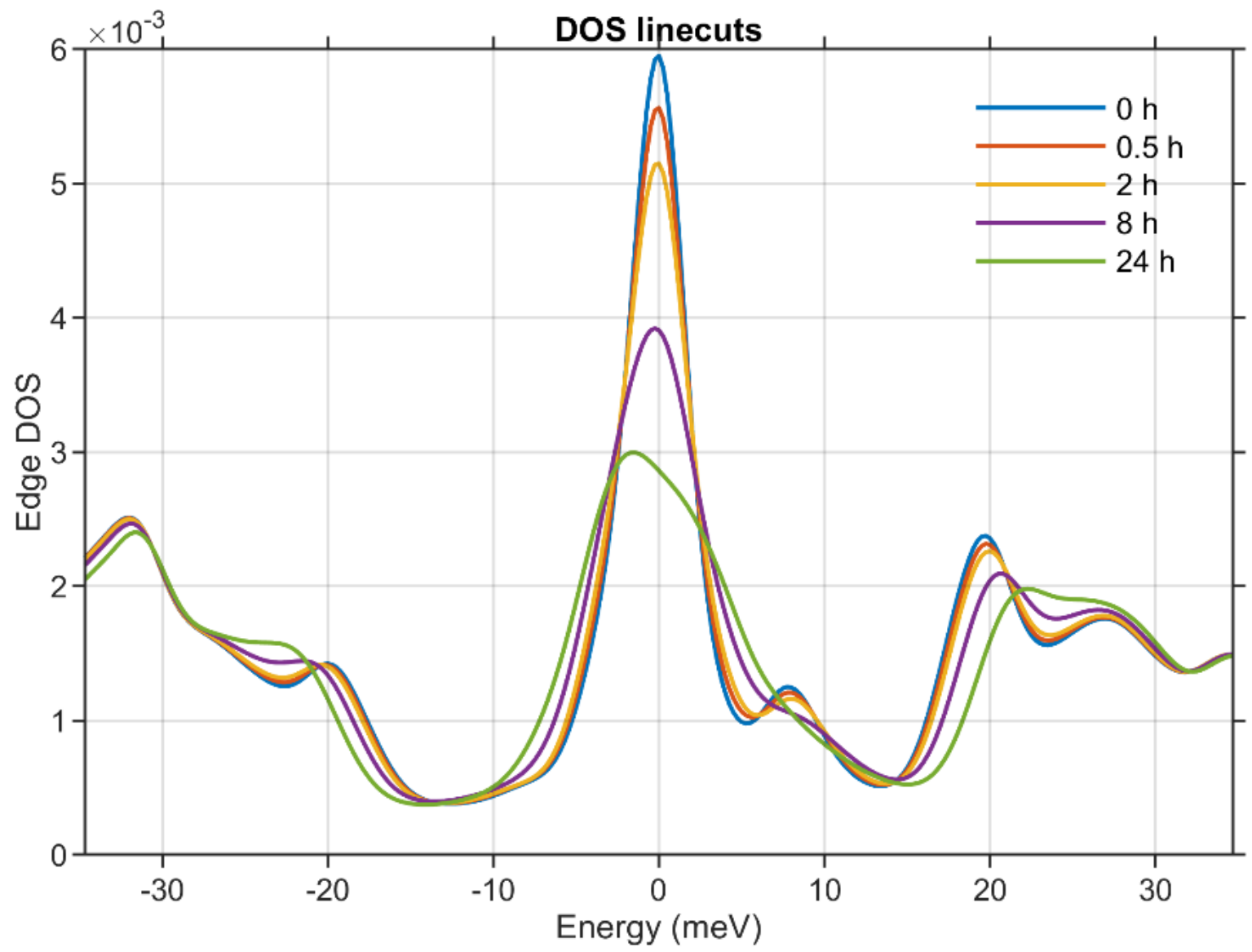

Revision to the manuscript

Discussion:

ment is thus evidently challenging in an experimental setting. Adsorbates provide a possible source of surface disorder. However, their spectroscopic signatures evolve gradually over several hours after cleavage (Supplementary Sec. VII), implying that any associated broadening of the flat-band states or modification of the superconducting gap should likewise develop progressively. Since we observe no corresponding time-dependent reconstruction of the low-energy spectra, adsorbates are unlikely to be the leading mechanism behind our observations. We also note that interaction-driven surface instabilities may also reconstruct

Methods:

approximate beam spot diameter (FWHM) of 40–50 μm. Several samples were measured over extended periods, as required for high-resolution measurements, by acquiring consecutive datasets that allowed us to track the temporal evolution of the spectra. Within the experimental reproducibility, we did not observe any systematic time-dependent degradation of the Fermi surface, disappearance of the dispersive bands, or emergence of new low-energy spectral features.

Supplementary Section VII:

VII. SHALLOW CORE-LEVEL PHOTOEMISSION SPECTRA

To assess the surface composition and its evolution during the measurement, we acquired photoemission spectra over the valence-band and shallow core-level energy ranges. The survey spectrum in Fig. 23a contains the expected shallow core levels of $La_{2-x}Sr_xCuO_4$. Possible impurity and adsorbate reference levels are indicated in red. Since shallow levels such as C 2s and N 2s depend strongly on the local chemical environment, they are represented by Gaussian envelopes rather than by unique binding energies. These envelopes therefore indicate plausible energy ranges but do not constitute direct elemental assignments. This chemical variability, together with the overlap of several candidate contributions, is consistent with the absence of a distinct peak that can be unambiguously assigned to an adsorbate. We therefore characterize the gradual surface degradation through the spectral changes near the valence band, where the strongest evolution is observed. Fig. 23b compares spectra measured shortly after cleavage and after $\Delta t = 18$ h 56 min. By contrast, the remaining features at larger binding energies are mostly unchanged. The intensity of the approximately 3 eV feature increases with time, whereas the weaker feature near 12 eV decreases in relative intensity. The reference binding energies were obtained from the NIST X-ray Photoelectron Spectroscopy Database [90, 91].

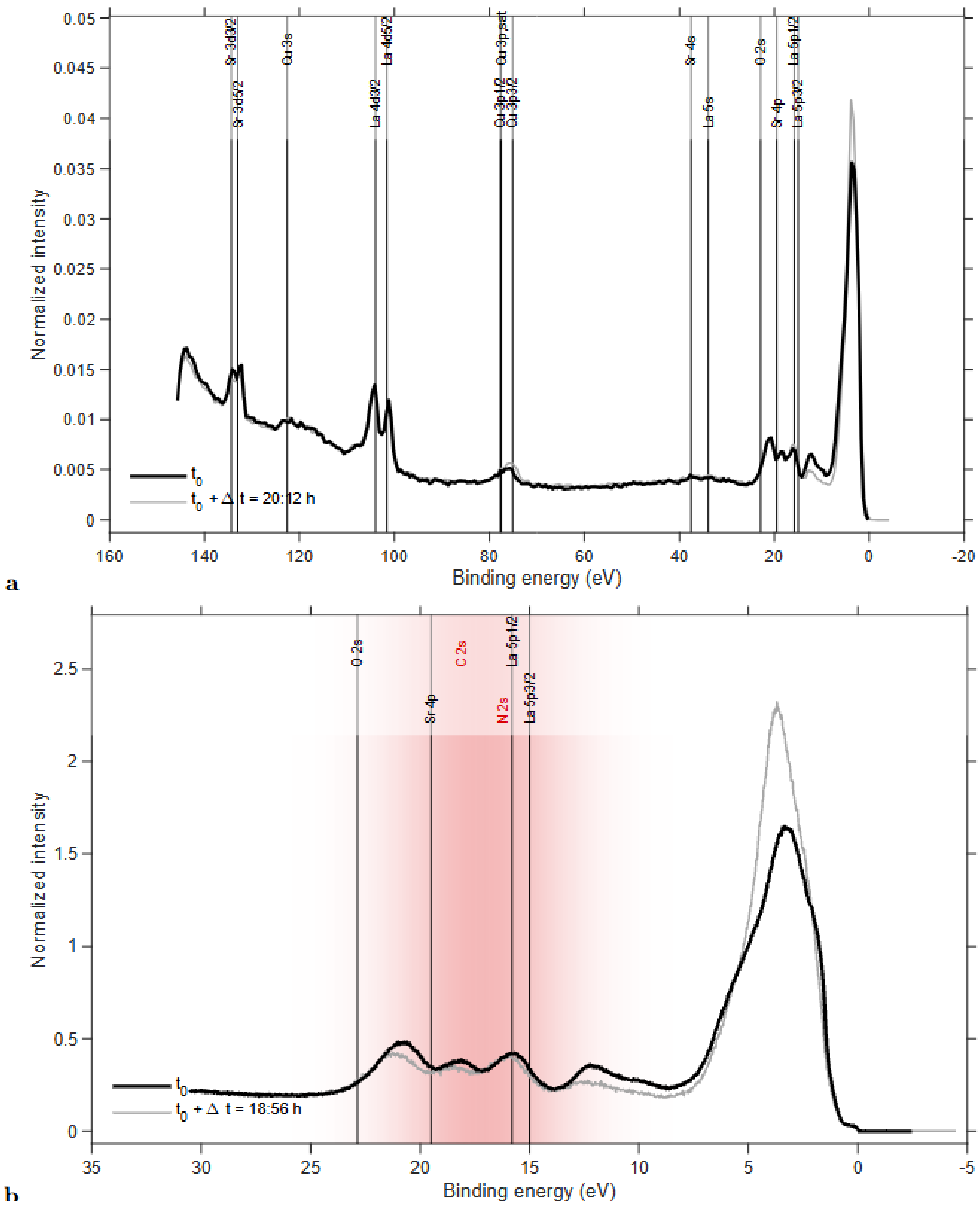

FIG. 23. **Shallow core-level and low-binding-energy photoemission spectra. a)** Survey spectrum measured at the beginning of the experiment and after Δt = 20 h 12 min with the expected LSCO shallow core-level binding energies indicated by black reference lines. **b)** Low-binding-energy spectra measured at the beginning of the experiment and after Δt = 18 h 56 min. Red lines and shaded envelopes indicate reference energies and chemically dependent energy ranges associated with possible impurities or adsorbates. Reference data were obtained from the NIST X-ray Photoelectron Spectroscopy Database [90, 91].

To quantify this evolution, the features near 3 eV and 12 eV were integrated after subtraction of their local backgrounds. Their peak positions were obtained from the maxima of the background-subtracted spectra using local quadratic interpolation. The resulting time dependences are shown in Fig. 24. The dominant changes in both spectral weight and peak position extend over approximately the first 10–15 h, after which the evolution appears to mostly saturate. The finite evolution time is plausible for a gradual accumulation of adsorbates, with substantial surface coverage being reached only several hours after cleavage. However, a quantitative relation between adsorbate coverage and either flat-band broadening or superconducting-gap suppression cannot be established from these spectra alone. Such a relation would require microscopic knowledge of the adsorbate species, their bonding to the surface, and their spatial distribution. Nevertheless, the observed timescale suggests that any adsorbate-induced modification of the low-energy electronic structure would develop progressively during the typical post-cleavage measurement period, rather than producing an immediate detrimental effect directly after cleavage. For this reason adsorbates are unlikely to be the leading mechanism explaining our ARPES measurements.

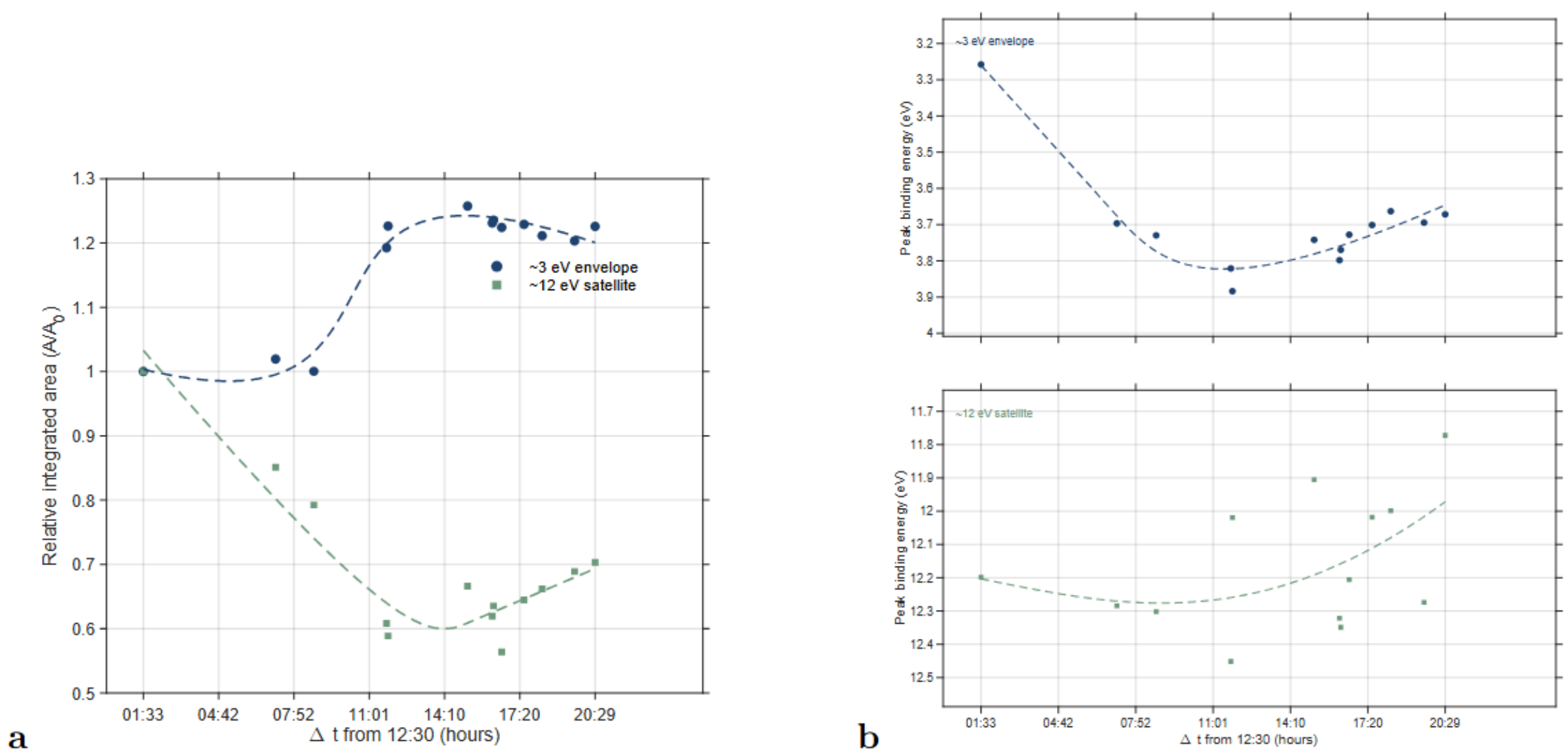

FIG. 24. **Time evolution of the low-binding-energy spectral features.** **a**) Integrated areas of the approximately 3 eV envelope and 12 eV satellite, normalized to their first measured values. **b**) Corresponding peak binding energies, with the 3 eV envelope shown in the upper panel and the 12 eV satellite in the lower panel. Dashed curves are smooth guides to the eye.

Comment 4: Third, I would like to raise a concern regarding the role of strong kz broadening inherent to the present measurement geometry. The manuscript estimates a kz broadening on the order of ~0.4 A-1, which implies that the measured spectra may contain contributions from a wide range of momenta,

including near-nodal regions where the superconducting gap is small or vanishing. Such mixing could effectively mask the superconducting gap even at nominally antinodal momenta. It would therefore be important to clarify whether the absence of a gap can be unambiguously attributed to pair-breaking or disorder effects, rather than being influenced by momentum broadening. In addition, it would be useful to clarify whether this effect is fully captured in the simulations, and to what extent it may affect the visibility of the superconducting gap.

Response to comment 4:

The referee raises a very important point here. We apologize for a factor-of-two error in our original estimate of the k_z broadening: the inelastic mean free path describes attenuation of the photoelectron escape probability, rather than of the wavefunction envelope. This leads to an additional factor of 0.5 compared to our original result. We have corrected this estimate and updated the main text and figures accordingly.

Nonetheless, the measured dispersions in Fig. 3(b) still enable the identification of the nodal and antinodal regions, despite being broadened. Our simulations shown in the same panel, which also include the effect of k_z-broadening, also do not show significant mixing of nodal and antinodal regions. We therefore conclude that k_z-broadening might reduce to some extent the contrast of superconducting-gap features and partially fill spectral weight at the Fermi level, but it would still allow for the identification of a superconducting gap if there were any via its temperature dependence.

For your information, the finite photoelectron mean free path is implemented in the simulations by multiplying the real-space eigenstates by an exponential decay factor into the bulk. The momentum-dependent spectral function is then obtained by Fourier transforming these exponentially weighted eigenstates. This procedure directly produces a finite broadening in the momentum component perpendicular to the surface, corresponding to the experimentally relevant k_z broadening. The characteristic scale is obtained from the inelastic mean free path estimated using the TPP-2M formula, giving $\Delta k_z \sim 0.2$ Å^{-1}. Importantly, in the clean calculation the zero-energy flat-band peak remains visible even after including the finite mean free path and associated k_z broadening. The absence of the flat-band peak in the experimental spectra therefore requires an additional broadening mechanism beyond the finite probing-depth effect alone.

Revision to the manuscript

Fermi surface characterization:

was explicitly included. The inelastic mean free path of the photoemitted electrons was evaluated using the Tanuma–Powell–Penn (TPP-2M) formula [61] (see Supplementary Section III D) and used to determine the magnitude of the k_z broadening, yielding a value on the order of 0.2 Å^{-1}. With this amount of broadening, the nodal and antinodal regions remain distinguishable. Such broadening can reduce the contrast of superconducting-gap features, but it would still allow for the identification of a superconducting gap if there were any.

Methods: Tanuma-Powell-Penn formula

with published estimates [84]. The inelastic mean free path describes the attenuation of the electron escape probability. Therefore, the corresponding wavefunction amplitude has an envelope $e^{-z/(2\lambda)}$ and the squared modulus of the Fourier transform of this exponentially decaying amplitude is a Lorentzian with full width at half maximum equal to $1/\lambda$. This allows us to obtain an estimate for the momentum broadening of $\Delta k_z \approx 0.21$ Å^{-1}.

Comment 5: In summary, this work presents a highly significant advance by enabling side-surface ARPES measurements on cuprate superconductors for the first time. The manuscript is well written and supported by high-quality data and simulations. I would be happy to recommend publication after the above concerns are adequately addressed.

Response

We thank again the reviewer again for their helpful comments and hope that their concerns were adequately addressed.

Response to Reviewer #2

Comment 1: This manuscript aims to investigate the zero-energy topological surface flat band on the side surface of a cuprate superconductor which has long been predicted theoretically but has not been directly observed by ARPES. The authors use a FIB-based preparation method to expose the otherwise inaccessible (110) surface and perform momentum-resolved ARPES measurements. Despite this technically demanding experimental approach the expected flat-band peak is not observed. The authors interpret this absence as a consequence of bulk disorder which broadens the surface-state spectral weight and suppresses the ARPES-detectable peak.

First I would like to emphasize that this work represents a highly notable attempt to overcome a long-standing limitation of ARPES namely that the accessible cleaving surface is usually fixed by the crystal structure. Previous STM and tunneling experiments have reported zero-bias peaks which have often been regarded as important evidence for topological surface flat bands. However the lack of momentum resolution in these probes makes it difficult to distinguish topological surface states from disorder-induced or interface-induced in-gap states. In this respect the authors' FIB-based sample preparation and subsequent ARPES measurement on a cuprate side surface constitute a challenging and important experimental advance. This approach opens a route to examine surface electronic structures in momentum space that were previously difficult to access. Given the surface characterization presented by the authors the absence of the expected topological surface flat band is itself an important result that highlights a

possible gap between theoretical expectations and experimental reality. The attempt to explain this result in terms of surface roughness and disorder is also a reasonable first line of investigation.

The authors further examine this scenario using real-space BdG simulations. In particular they solve a self-consistent BdG gap equation to analyze how surface roughness and disorder affect the pairing field and the surface spectral function. Such calculations require repeated reconstruction and diagonalization of the BdG Hamiltonian at each iteration and therefore demand substantial computational resources as well as careful treatment of convergence and numerical stability. In this sense the calculations go beyond a purely qualitative argument and represent a meaningful microscopic attempt to examine order-parameter suppression at the surface and disorder-induced broadening of the surface state. Nevertheless there remain several issues in the computational setup and presentation. These limitations raise the question of whether the disorder-based interpretation can be sufficiently generalized from the calculations currently presented.

Response to comment 1:

We thank the reviewer for recognizing the technical advance represented by the novel cleaving technique, the importance of a momentum-resolved measurement of a side-surface of cuprates, as well as acknowledging the attempt at providing a quantitative microscopic explanation for our observations.

Comment 2: The manuscript also emphasizes in the Introduction that the topological surface flat band on a cuprate side surface is highly susceptible to various symmetry-breaking instabilities because of its large degeneracy and high density of states. The authors discuss several possible competing surface orders including time-reversal-symmetry-breaking states, mixed-parity superconductivity, phase-crystal states and magnetic order. They also mention several alternative scenarios in connection with previous ZBCP observations such as interface states disorder-induced bound states and facet mixtures. However when interpreting the absence of the flat-band peak in the present experiment the manuscript does not sufficiently discuss how these scenarios may be compatible with or constrained by the present result. The absence of the peak is mainly explained using BdG calculations with surface roughness and bulk disorder while the possible effects of surface instabilities, time-reversal symmetry breaking, phase-crystal formation or edge magnetism on the flat-band spectral weight and ARPES visibility are only limitedly discussed. In addition although interface states and facet mixtures may be partly constrained by the surface characterization and the momentum-resolved nature of ARPES the possible influence of microscopic domain or facet structures on the flat-band spectral weight could be discussed more explicitly. Such a discussion would not weaken the authors' interpretation. Rather it would clarify the relative position of possible explanations and improve the balance and persuasiveness of the manuscript. Therefore the generality and conclusiveness of the present interpretation would benefit from a more balanced discussion of alternative scenarios and a clearer statement of the limitations of the calculations. Since the central interpretation relies substantially on real-space BdG simulations the following points should be considered.

Response to comment 2:

We thank the reviewer for pointing out that the relationship of our model to alternative scenarios for the suppression of the zero-bias peak should be clarified.

The model considered in the works cited in our manuscript for the disorder-induced bound states are closely related to the minimal disorder model used in our calculations, although they address somewhat different physical questions.

- Sulangi et al. did not specifically study topological edge flat bands. Instead, they investigated how different forms of disorder in a two-dimensional d-wave superconductor affect the quasiparticle DOS and localization, considering random onsite disorder, dilute strong scatterers, and smooth off-plane disorder potentials. They found that some disorder realizations can generate

finite or enhanced zero-energy DOS, which may be relevant to certain zero-bias-peak observations in tunneling experiments. This situation is distinct from the present experiment, where the expected momentum-resolved flat-band peak on the side surface is absent. We therefore cite Sulangi et al. mainly as a possible alternative explanation for some ZBCPs reported in the literature and to place our disorder model in the context of previous theoretical works on cuprates.

- Kalenkov et al. are more directly connected to the surface problem, since they considered impurity-induced bound states near a pair-breaking d-wave surface, including surface impurity layers and bulk impurity scattering. Our Anderson-type onsite disorder should therefore be understood as a minimal short-range scalar disorder model, rather than as a microscopic model of every possible disorder source. The essential low-energy effect captured by this model is the presence of large-momentum-transfer scattering, which can couple flat-band states from opposite chirality sectors and thereby broaden or lift the zero-energy degeneracy. Other forms of short-range disorder, including point scatterers, surface impurities, or unresolved atomic-scale faceting, are expected to have a qualitatively similar effect if they generate comparable momentum transfer, whereas smoother disorder should be less efficient.
- Regarding faceting, we have considered two limits of geometric disorder. First, the AFM-derived surface profile was incorporated directly by removing sites along the edge according to the measured height profile, thus setting a lower limit to the spatial frequency of the faceting. Second, a high-spatial-frequency probabilistic roughness model was used to test the limiting case in which clean edge segments are destroyed down to the unit-cell scale, i.e. the upper bound to the frequency of the faceting. We find that the AFM-derived roughness does not remove the flat-band peak in the simulations, whereas sufficiently high-frequency roughness can broaden it more strongly. This constrains facet-based explanations at the length scales resolved by AFM, while leaving open the possibility of unresolved atomic-scale faceting or microscopic domain structure.

We also agree that surface instabilities such as time-reversal-symmetry breaking, phase-crystal formation, mixed-parity surface superconductivity, or edge magnetism could in principle reconstruct, split, or redistribute the flat-band spectral weight. In fact, in our self-consistent calculations, we do find a small subdominant imaginary s-wave component near the edge. Its effect is, however, small on the scale of the experimental energy resolution of 3-4 meV[6], and after convolution with the experimental resolution it does not visibly alter the flat-band peak. We therefore do not find that this instability alone can explain the complete absence of the ARPES-visible flat-band feature. To the best of our knowledge, the energy scale of most, if not all, of these instabilities lies below the limit of our experimental resolution and are therefore not the leading mechanism for the flat band broadening.

Nevertheless, we do not claim that the present experiment categorically excludes all interaction-driven or disorder-based scenarios of flat band suppression. We have revised the manuscript to make clear that our disorder calculation provides a minimal sufficient mechanism within the present BdG framework, rather than an extensive microscopic investigation.

Revision to the manuscript

Introduction

---

[6] Daghero, D., Gonnelli, R. S., Ummarino, G. A., & Stepanov, V. A. (2003). Possible d+id scenario in $La_{2-x}Sr_xCuO_4$ by point-contact measurements. *International Journal of Modern Physics B,* 649–654. https://doi.org/10.1142/S0217979203016376

gether [47–49]. Several mechanisms have been proposed to reconcile these disparate findings, including the presence of inter-domain interfaces [50], disorder-induced low-energy spectral weight in the quasiparticle DOS [51], impurity-induced bound states near pair-breaking surfaces [52], and the coexistence of multiple facets with different orientations [20, 34]. However, the lack of momentum resolution in tunneling experiments fundamentally limits the ability to distinguish topological flat bands from topologically trivial zero-bias features. A surface-

Broadening of the surface states and gap frustration by pair breaking

sensitivity of the flat-band peak to short-length-scale scattering. Other short-range disorder sources, such as pointlike impurities, strongly localized defect potentials, or sufficiently abrupt surface impurity potentials, are expected to have a qualitatively similar effect if they generate comparable large-momentum-transfer scattering. By contrast, spatially smooth

Discussion

in an experimental setting. We also note that interaction-driven surface instabilities may also reconstruct the flat-band spectral weight. For instance, in our self-consistent calculations we find a small subdominant imaginary s-wave component localized near the edge (see Supplementary Section VI Q). This type of instability can split the zero-energy surface states, but since such an order remains subdominant to the primary d-wave gap, its characteristic energy scale is expected to be smaller than the superconducting gap. Consistently, after convolution with the experimental energy resolution, the subdominant component in our calculations does not visibly alter the flat-band peak. Other possible instabilities could in principle also split or redistribute the flat-band spectral weight. However, such orders are typically expected to develop at a secondary transition temperature below the superconducting transition temperature. This both reflects their small characteristic energy scale and suggests that a dominant instability-driven explanation should produce a measurable temperature-dependent reconstruction of the low-energy spectra. Since we do not observe such a reconstruction across the measured temperature range, these instabilities, are unlikely to be the dominant mechanism responsible for our observations. A full quantitative treatment of all such states would require additional interaction channels and order parameters beyond the scope of the present work, which is to provide a minimal sufficient mechanism within our BdG framework.

Supplementary

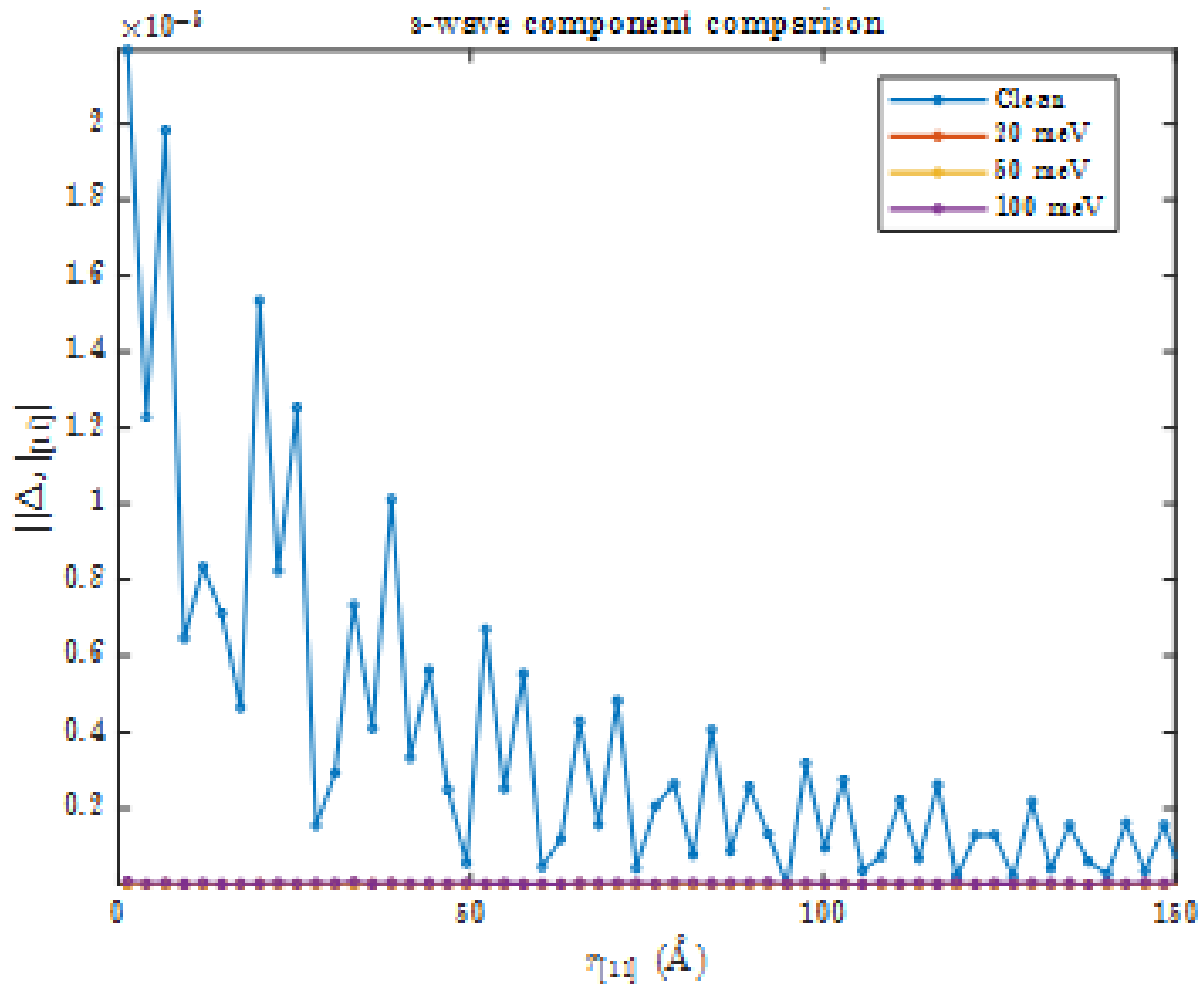

FIG. 22. Spatial profile of the bond-resolved imaginary s-wave superconducting component for the clean system and disorder strengths of 20, 50, and 100 meV. This component is very sensitive to onsite disorder (because of its small associated order parameter) and effectively vanishes already for a 20 meV disorder.

Comment 3:

1. A more systematic examination of disorder strength is needed. The manuscript introduces Anderson disorder as a Gaussian onsite mass term and mainly presents results for a standard deviation of 100meV. This value is considerably larger than the superconducting gap scale. Of course considering that impurity potentials in cuprates can reach several tens to several hundreds of meV the choice of 100meV is not necessarily unrealistic. However if the absence of the flat-band peak is to be attributed to disorder-induced broadening it would be useful to show how the flat-band peak the bulk superconducting gap and the edge spectral weight evolve as the disorder strength is varied. For example results for several representative disorder strengths such as 20meV 50meV and 100meV would help clarify whether the suppression of the flat-band peak depends sensitively on a particular disorder scale or is robust over a broader range.

Response to comment 3:

We agree that a systematic sweep of disorder strength is useful for assessing the robustness of the mechanism. We have included in the Supplementary the calculations corresponding to 20meV and 50meV too and revised the main text accordingly.
Regarding the magnitude of the superconducting gap, in our calculations we adjusted the pairing strength factor in the self-consistent gap equation so as to get a bulk gap in agreement with experimental values. So the result of our calculations compare different disorder potentials given the same bulk spectral gap magnitude.
We also note that the disorder strength cannot be interpreted independently of the disorder model. A Gaussian onsite potential, a dilute impurity distribution, spatially extended dopant potentials, oxygen-vacancy disorder, hopping disorder, and correlated disorder can produce different amounts of large-

momentum-transfer scattering for the same nominal root-mean-square amplitude. Therefore, a strength sweep for one specific model would establish robustness only within that model, not a unique quantitative threshold for LSCO. For this reason, we emphasize that the main qualitative result of our work is that for any given model, a sufficiently short-length-scale potential disorder can efficiently broaden the flat-band manifold.

Revision to the manuscript

Broadening of the surface states and gap frustration by pair-breaking:

lated onsite disorder used here. For the disorder realization shown in Fig. 4d-e, we find that the inclusion of a random mass term at each site, with a magnitude drawn from a Gaussian distribution with a standard deviation of 100 meV, lifts the degeneracy of the flat band and strongly suppresses the associated peak. A comparison of different disorder strengths shows that the flat-band peak remains partially visible for weaker disorder, see the Supplementary Section. VI P where additional disorder realization are also compared. We further observe a

Supplementary:

P. Different disorder strengths

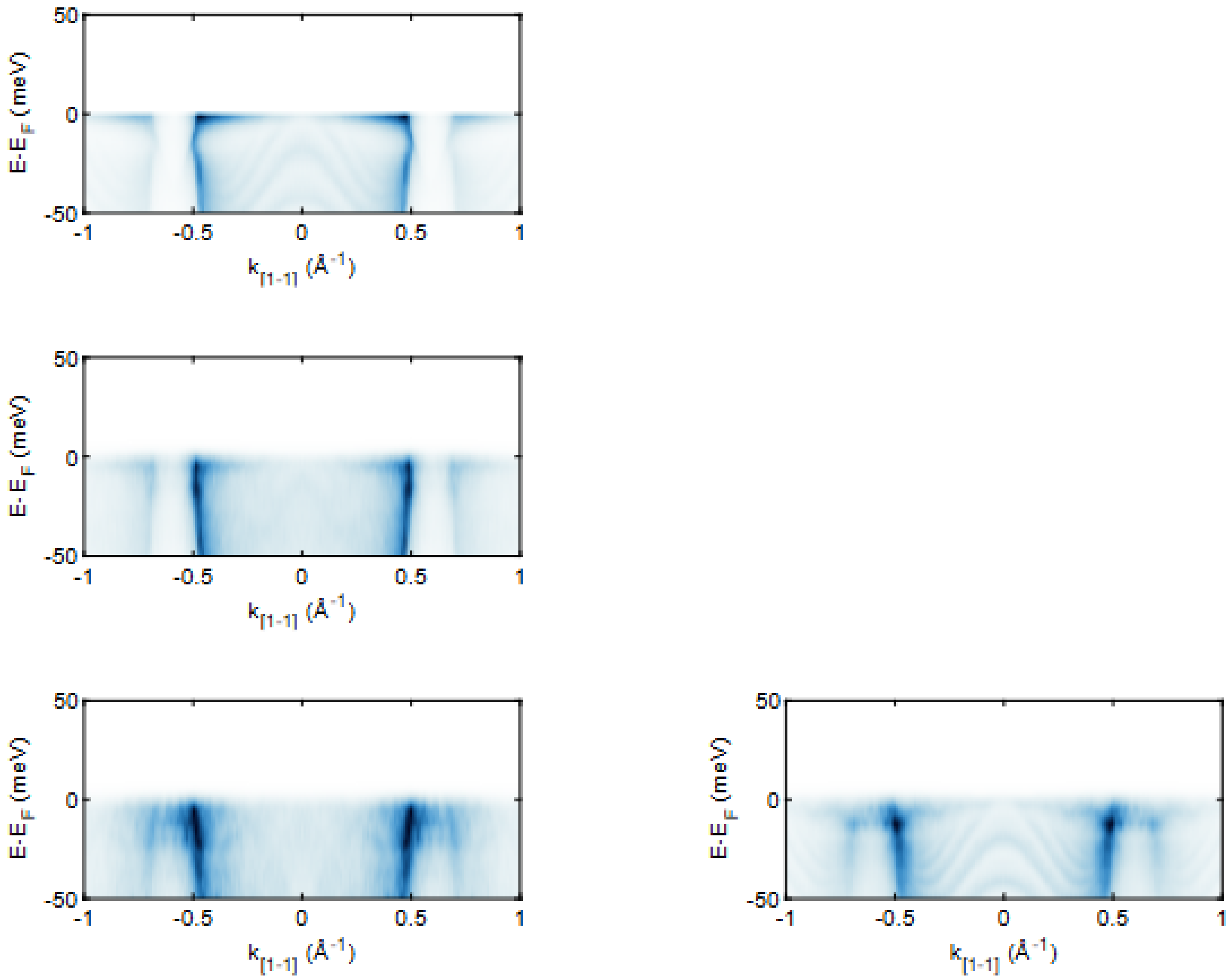

FIG. 20. The same spectral function shown in the main text, now evaluated for different disorder strengths. From top to bottom, the first three panels correspond to disorder strengths of 20 meV, 50 meV, and 100 meV. The bottom-right panel also corresponds to 100 meV disorder, but with the disorder potential applied only to the 6 atomic layers closest to the edge.

Comment 4:

2. The representativeness of the disorder model requires further discussion. The onsite disorder used in the manuscript is a reasonable minimal model for demonstrating that bulk inhomogeneity in cuprates can broaden the flat-band peak. In particular site-to-site Gaussian disorder efficiently introduces short-length-scale scattering and is therefore a suitable starting point for testing whether the flat-band surface state can be broadened by disorder. However disorder in real cuprates may arise from dopants, oxygen vacancies, extended impurity potentials and spatially correlated disorder. These microscopic profiles can directly affect the strength of large-momentum-transfer scattering and the degree of surface-state broadening. For instance spatially smooth or extended disorder may produce weaker scattering between opposite chirality sectors than short-range onsite disorder and may therefore suppress the flat-band peak less strongly than

in the present calculation. Although the authors acknowledge in the Discussion that disorder can take various forms this discussion is not sufficiently connected to the representativeness of the specific disorder model used in the simulations. The authors do not necessarily need to compare all possible disorder types computationally. However a more explicit qualitative discussion is needed regarding how well the chosen disorder model represents the disorder landscape of LSCO and whether this choice may overestimate the suppression of the flat-band peak. Since the key conclusion of the manuscript relies strongly on disorder-induced broadening clarifying the physical meaning and limitations of the chosen disorder model is important.

Response to comment 4:

The purpose of the onsite disorder calculation is to isolate the effect of short-length-scale bulk inhomogeneity on the topological surface flat band. Since the flat-band states of opposite chirality occur at different momenta, their broadening requires disorder components with sufficiently large momentum transfer. A site-to-site random onsite potential has a broad Fourier spectrum and therefore efficiently tests the sensitivity of the flat-band peak to such short-range scattering. In this sense, the model represents the short-range component of the disorder landscape arising from dopants, oxygen defects, local chemical inhomogeneity, and other microscopic imperfections. At the same time, as we already mentioned in the main text, we agree that real cuprate disorder is more complex. Dopant potentials, oxygen vacancies, and structural inhomogeneities may generate a mixture of short-range, extended, and spatially correlated disorder. The efficiency of flat-band broadening will then depend not only on the overall disorder strength, but also on the disorder correlation length and on the weight of the disorder potential at the momentum transfer connecting opposite chirality sectors. Spatially smooth or extended disorder would have reduced large-momentum-transfer components and would therefore be expected to broaden the flat band less efficiently than the uncorrelated onsite disorder used in our calculation.

We have therefore clarified this point in the main text.

Revision to the manuscript

Broadening of the surface states and gap frustration by pair-breaking:

section VI C). The efficiency of this broadening is controlled not only by the overall disorder strength, but also by the disorder correlation length and by the Fourier weight of the disorder potential at the momentum transfer connecting opposite chirality sectors. Since eigenstates of opposite chirality reside at opposite momenta, this inter-sector coupling necessarily involves large-momentum-transfer scattering processes. Uncorrelated onsite disorder has a broad Fourier spectrum and therefore provides an efficient limiting case for testing the sensitivity of the flat-band peak to short-length-scale scattering. Other short-range disorder sources, such as pointlike impurities, strongly localized defect potentials, or high-frequency surface impurity potentials, are expected to have a qualitatively similar effect if they generate comparable large-momentum-transfer scattering. By contrast, spatially smooth or extended disorder potentials would have reduced large-momentum-transfer components and would therefore be expected to broaden the flat-band peak less efficiently than the uncorrelated onsite disorder used here. For the disorder realization shown in Fig. 4d-e, we find that

Comment 5:

3. It is unclear whether disorder realizations were averaged. Anderson disorder can produce different local spectral weights and different degrees of flat-band broadening depending on the random configuration. Therefore an ensemble average over several configurations would be desirable. Although some degree of self-averaging may be expected in a sufficiently large system the flat-band surface state considered here can be sensitive to spatial inhomogeneity near the edge and to the details of boundary segments. Thus realization dependence should not be assumed to be negligible a priori. The manuscript does not specify how many disorder configurations were used whether the results were averaged or how large the variation between realizations is. It would therefore be helpful to clarify whether the presented spectra correspond to a representative realization or to an ensemble-averaged result.

Response to comment 5:

We agree that this should have been stated explicitly. The spectra shown in the manuscript correspond to a single disorder realization, not to an ensemble average. The reason for this is mostly computational. The self-consistent calculations were performed on a large slab containing 280×200 lattice sites, corresponding to $56{,}000$ real-space sites and a BdG Hamiltonian dimension of order 10^5 which needs to be diagonalized at each self-consistency iteration. A systematic ensemble average over disorder realizations at the same system size and with full self-consistency is therefore computationally very expensive. Reducing the system size would proportionally reduce the degree of self-averaging, so by itself shouldn't lead to more representative results.

Nevertheless, we expect substantial self-averaging in the quantities that we compute. The simulated spectral function is not a local density of states at a single point, rather it is obtained by effectively averaging over the full edge length by Fourier transforming all the real-space eigenstates weighted by an exponential decay representing the finite photoelectron mean-free-path in the direction perpendicular to the edge. This means that effectively the spectral function that we present in our manuscript is an average contribution from several hundred sites. Therefore, the result is not expected to be sensitive to a single impurity or isolated local edge segment particular of a specific disorder realization. Similarly, the experimental ARPES measurement, is also an average in this case over a beam spot area of approximately 40–50 μm^2.

We agree, however, that this does not eliminate realization dependence in principle. We have therefore revised the manuscript to state that the disorder calculation is a representative realization demonstrating a broadening mechanism, not an average result over multiple realizations. We have also added in the supplementary section a comparison of two random realizations for each of the 20 meV, 50 meV and 100 meV disorder showing few qualitative variations (Fig. 19-20).

Revision to the manuscript

Broadening of the surface states and gap frustration by pair-breaking:

For the disorder realization shown in Fig. 4d-e, we find that the inclusion of a random mass term at each site, with a magnitude drawn from a Gaussian distribution with a standard deviation of 100 meV, lifts the degeneracy of the flat band and strongly suppresses the associated peak. A comparison of different disorder strengths shows that the flat-band peak remains partially visible for weaker disorder, see the Supplementary Section VI N where additional disorder realization are also compared. We further observe a relative increase

Methods:

The disorder spectra shown in the main text correspond to a single representative realization rather than to an ensemble average. This choice was made because the self-consistent calculation is performed on a 280×200 lattice, corresponding to 56,000 real-space sites and a BdG Hamiltonian of dimension 112,000, which must be diagonalized at each self-consistency iteration. The calculated ARPES spectral function is nevertheless partially self-averaging, since it is obtained from eigenstates extending along the full simulated edge and weighted over the photoelectron escape depth, rather than from the local density of states at a single site. The full Hamiltonian was then diagonalized in real space and to account

New Supplementary section: Different Disorder Strength and Realizations:

N. Different disorder strengths and realizations

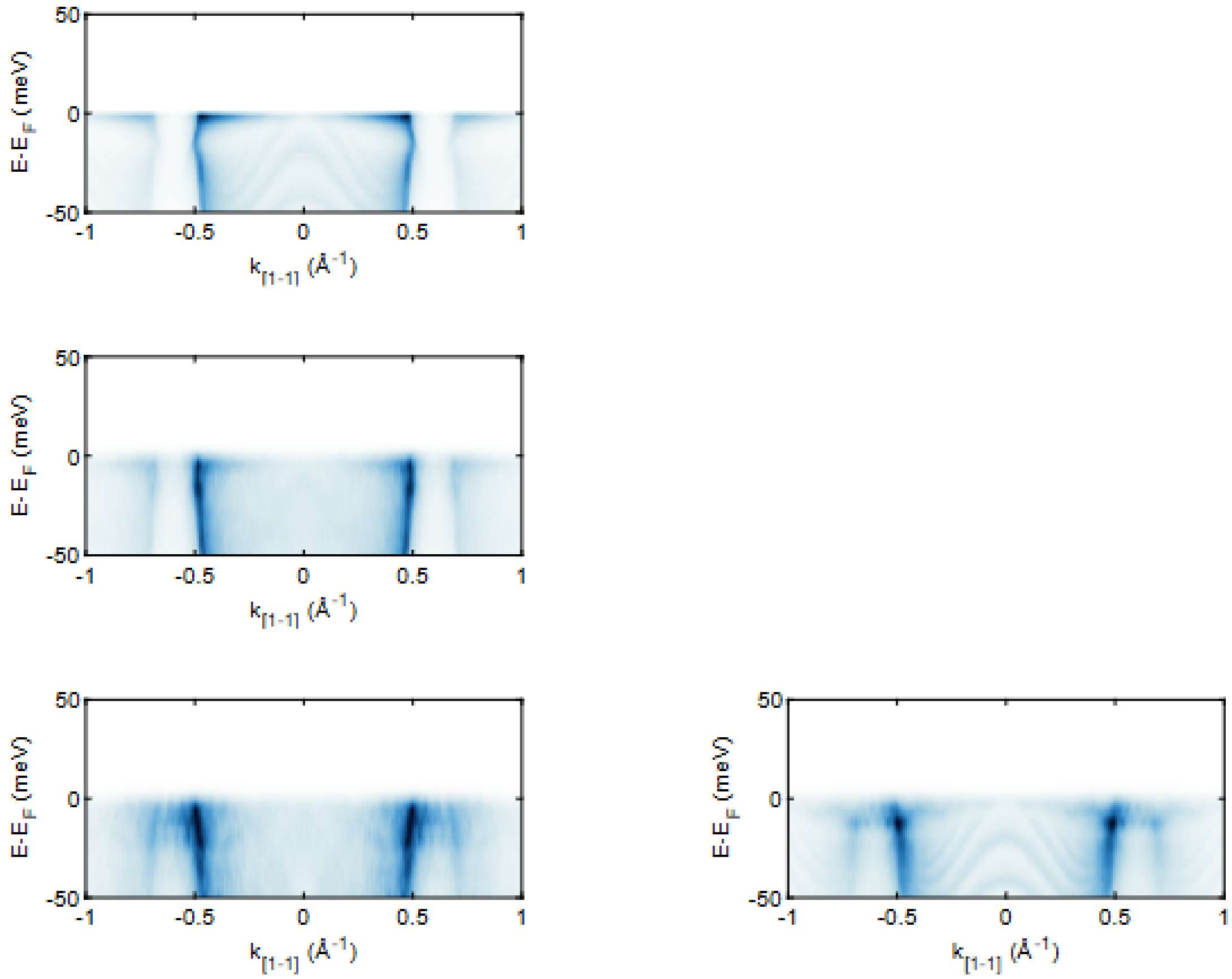

FIG. 17. The same spectral function shown in the main text, now evaluated for different disorder strengths. From top to bottom, the first three panels correspond to disorder strengths of 20 meV, 50 meV, and 100 meV. The bottom-right panel also corresponds to 100 meV disorder, but with the disorder potential applied only to the 6 atomic layers closest to the edge.

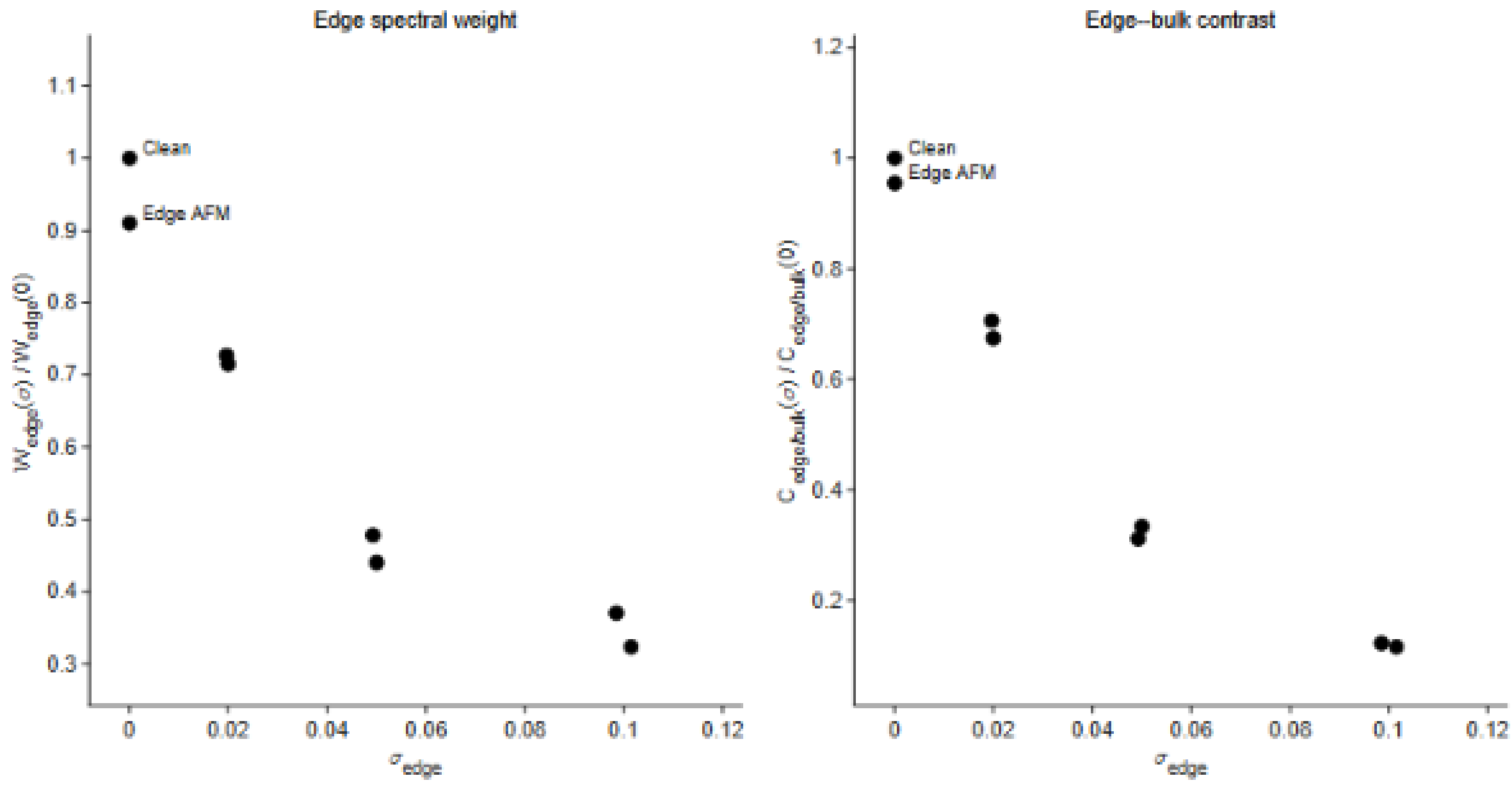

FIG. 20. Disorder dependence of the zero-energy flat-band spectral weight at the edge. The horizontal axis in both panels is the standard deviation of the edge disorder, $\sigma_{\rm edge}$, computed directly from the disorder profile used in each simulation. The spectral weight is integrated over a low-energy window $E \in [-2.5, 2.5]$ meV. Left: normalized integrated edge spectral weight, $W_{\rm edge}(\sigma)/W_{\rm edge}(0)$, where $W_{\rm edge}(\sigma) = \int_{-2.5\,\rm meV}^{2.5\,\rm meV} {\rm DOS}_{\rm edge}(E,\sigma)\,dE$. Right: normalized edge–bulk contrast, $C_{\rm edge/bulk}(\sigma)/C_{\rm edge/bulk}(0)$, where $C_{\rm edge/bulk} = W_{\rm edge}/W_{\rm bulk}$. The bulk spectral weight is evaluated at the center of the slab using the same number of layers as used for the edge region. All quantities are normalized to the clean zero-disorder reference.

Comment 6:

4. The statistical character of surface roughness is not sufficiently analyzed. The manuscript incorporates the AFM-measured roughness profile through edge-site removal and concludes that the experimentally resolvable roughness is insufficient to remove the flat-band peak. This is a meaningful approach because it directly uses the measured surface information. However the influence of surface roughness cannot be fully assessed from the RMS roughness alone. Even for the same roughness amplitude a long lateral correlation length can preserve clean edge segments and allow the flat band to survive whereas unit-cell-scale high-frequency roughness can enhance large-momentum scattering and broaden the surface state more efficiently. The authors mention that high in-plane spatial frequency roughness may be important but the spectral function is not systematically examined as a function of roughness autocorrelation length or high-frequency components. Thus the conclusion that the AFM-derived roughness is insufficient to suppress the flat band is convincing within the measured roughness profile but it does not fully characterize the broader effect of surface roughness disorder.

Response to comment 6:

We agree with the reviewer's distinction between roughness amplitude and roughness correlation length. We have already considered this but we have not clarified this point in the manuscript.

Our calculation did not use only the RMS roughness as a scalar value. We selected and incorporated an arbitrary spatial section of the AFM height profile directly into the tight-binding slab by removing sites along the edge according to the measured profile. The RMS value is quoted as a compact experimental measure of the surface topography, but the simulation uses the full measured spatial profile at the resolution available from AFM.

We also considered a separate probabilistic roughness model, denoted $GE_{0.5}$ in the manuscript, to approximate the opposite limit of high-spatial-frequency roughness. In this case, the roughness varies on the shortest length scale accessible in the lattice model and destroys contiguous clean edge segments much more efficiently. This calculation was included precisely to test the reviewer's point that short-correlation-length roughness can have a stronger effect than long-correlation-length roughness of similar RMS amplitude.

While we did attempt to parametrize the AFM-measured surface roughness in terms of both RMS amplitude and correlation length, unfortunately these two quantities do not uniquely characterize the disorder. Surfaces with the same roughness and nominal correlation length can still differ in the height distribution, the detailed shape of the autocorrelation function or power spectral density, anisotropy, local slope and curvature statistics, and higher-order spatial correlations such as clustering or connectivity of protrusions and depressions. These additional degrees of freedom make it difficult to isolate, in a controlled parametrized model, which specific morphological features are most relevant for the observed physics. For this reason, rather than attempting to reconstruct the disorder from a limited set of scalar descriptors, we incorporated a representative spatial section of the AFM profile directly into the tight-binding geometry. This choice preserves the experimentally measured morphology within the available AFM resolution. It is also justified by the fact that the AFM dataset was largely homogeneous and did not contain obvious outlier regions.

Revision to the manuscript

Broadening of the surface states and gap frustration by pair breaking:

To account for these observations, we incorporated geometric edge disorder in our simulations, implemented by removing sites along the edge using an arbitrary section of the experimentally measured surface topography (Fig. 2e) obtained by AFM (Methods III E).

Methods: Two-dimensional Slab:

field whose local value was determined self-consistently (see the Methods section III F). The geometric edge disorder was implemented using a representative line profile extracted from the AFM topography. To assess whether this profile was statistically representative of the measured surface morphology, we analyzed randomly selected AFM line cuts with the same physical length as the simulated edge (Supplementary Sec. VI O). The line profile used in the main-text tight-binding calculation lies within the 10th–90th percentile range of this distribution for both the RMS roughness and the autocorrelation length, but was deliberately chosen from the lower-autocorrelation-length side of this representative interval. This choice provides a conservative upper estimate of the effect of the measured geometric edge disorder. For a fixed roughness amplitude, a shorter autocorrelation length corresponds to more rapidly varying edge morphology, enhancing large-momentum scattering and therefore maximizing the expected disorder-induced mixing of edge-state momenta. Consequently, if this relatively short-correlated profile does not produce sufficient broadening of the edge states, smoother profiles with longer autocorrelation lengths are unlikely to account for our observations.

O. AFM topography

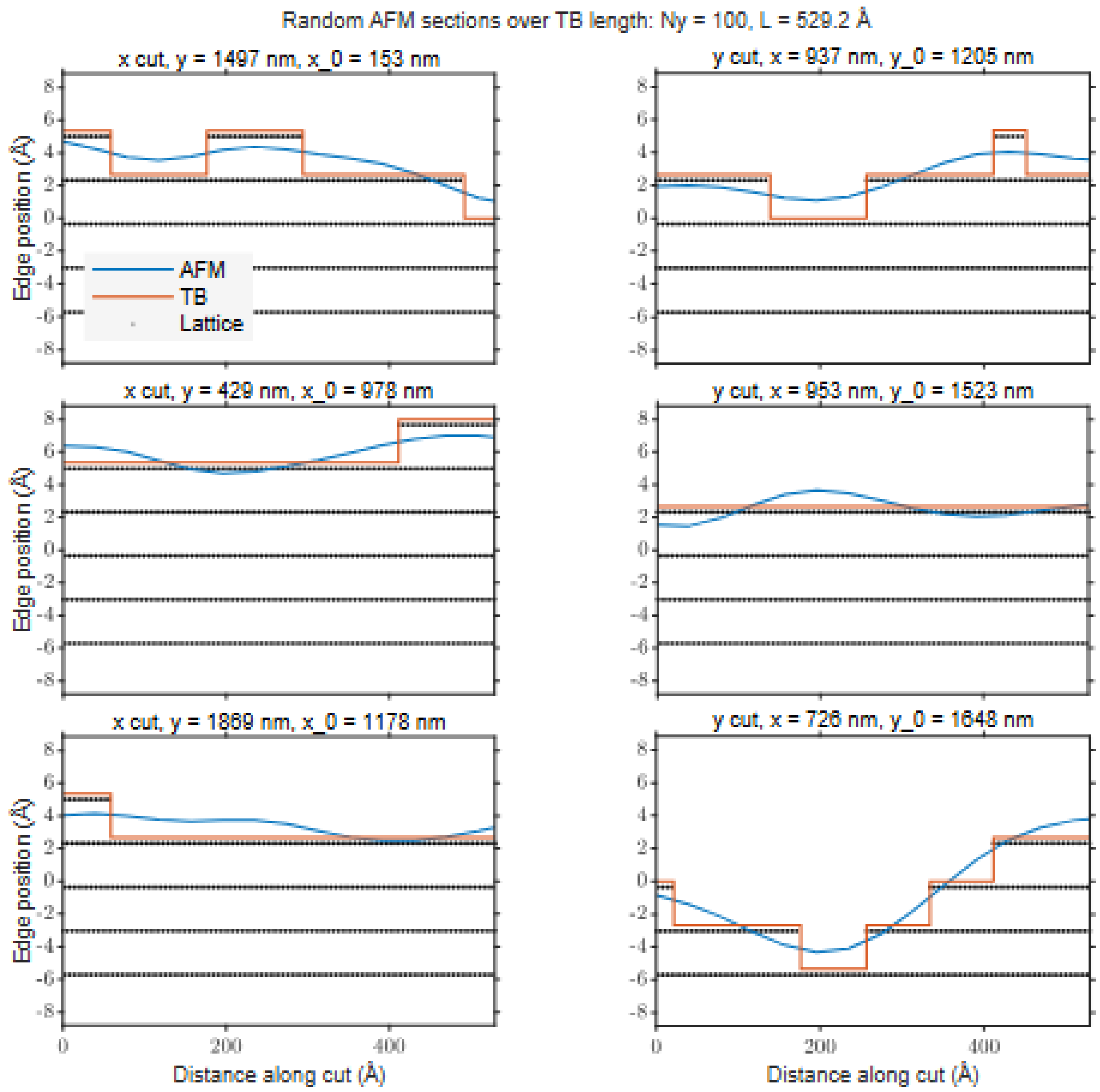

FIG. 18. Representative AFM-derived edge profiles used to construct the corresponding tight-binding edge geometries. Each panel shows a line cut extracted from the AFM topography, converted to an edge displacement in Å, together with the discretized tight-binding edge profile and the retained lattice sites. The first panel shows the AFM section used for the calculations presented in the main text. The labels "x cut" and "y cut" denote line cuts taken along the fast-scan and slow-scan directions of the AFM image, respectively. For each cut, the listed x_0 and y_0 denotes the distance of the plotted segment from the edge of the AFM data.

To assess the representativeness of the AFM-derived edge profile used in the main-text calculation, we randomly sampled line cuts from the AFM topography with the same physical length as the simulated tight-binding edge. For each line cut we computed the RMS roughness R_q and an autocorrelation length ξ. The AFM section used in the main-text calculation lies within the region bounded by the 10th and 90th percentiles of the random-section distribution, indicating that it is representative of the measured surface morphology rather than an outlier.

Within this representative set, the chosen section has a relatively short autocorrelation length. This choice is conservative for the purpose of estimating the effect of AFM-resolvable roughness on the flat-band surface state, since shorter lateral correlation lengths enhance large-momentum scattering and are expected to broaden the surface state more efficiently than smoother profiles with the same RMS roughness. Therefore, the persistence of the flat-band peak for this AFM-derived profile places an upper bound on the broadening attributable to the experimentally resolved surface morphology.

We emphasize that this bound is limited by the lateral resolution of the AFM measurement. Roughness components with wavelengths below the AFM lateral-resolution limit, in particular unit-cell-scale height variations, are not contained in the measured profile. When the AFM profile is mapped onto the lattice model it therefore produces comparatively extended flat segments relative to the atomic length scale. The separate probabilistic $GE_{0.5}$ model is used in the main text to approximate this opposite, high-spatial-frequency limit.

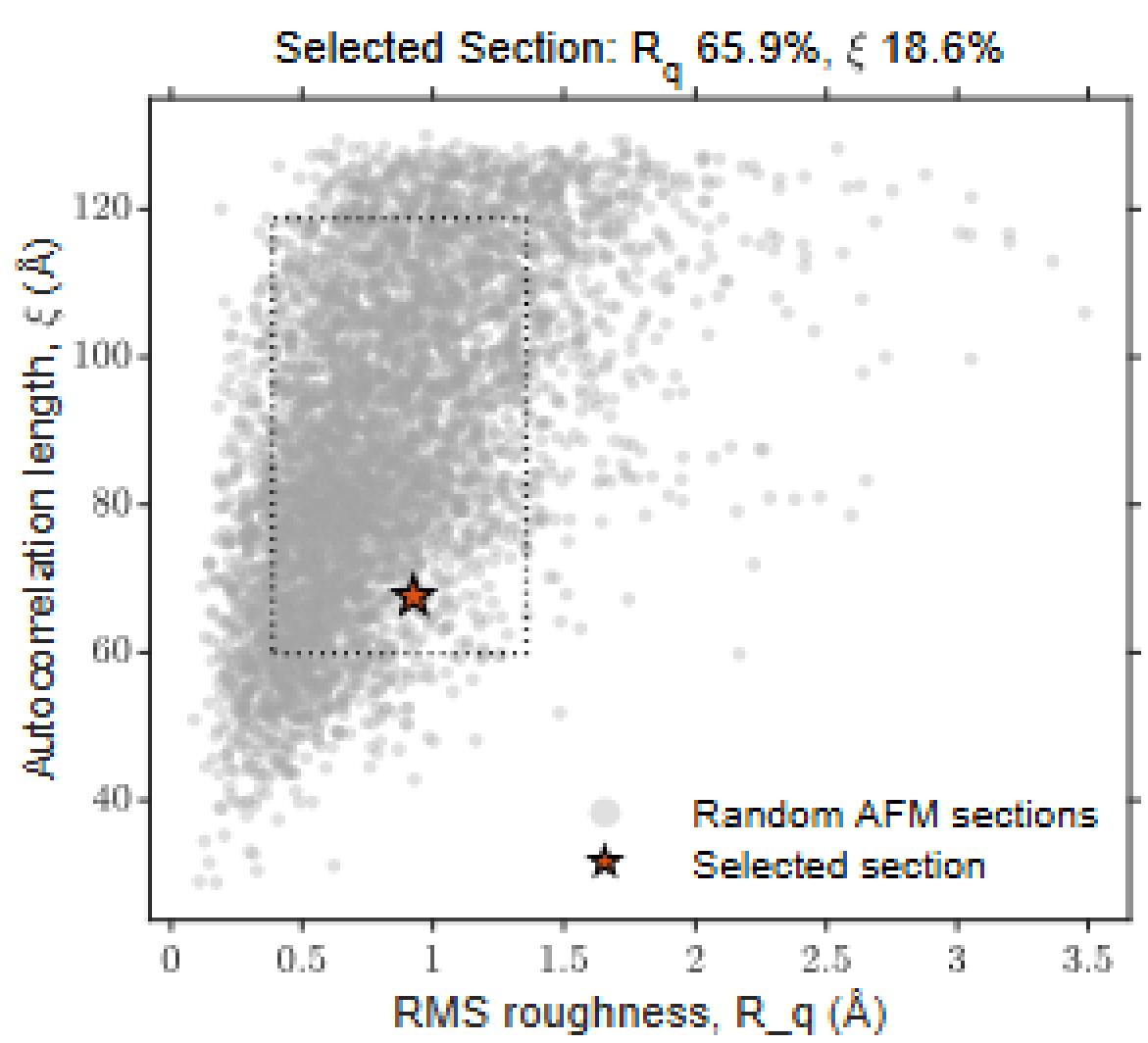

FIG. 19. Representativeness analysis of the fixed AFM section used in the first panel of the previous Figure. Gray points show randomly sampled AFM sections of the same physical length as the tight-binding model. Each section is characterized by its RMS roughness, R_q, and autocorrelation length, ξ, computed from the AFM-derived edge profile. The highlighted marker denotes the arbitrary section used in our calculations. The dotted rectangle indicates the region bounded by the 10th and 90th percentiles of the random-section distribution.

Comment 7:

5. The effects of edge disorder and bulk disorder are not sufficiently separated. Since the flat-band surface state is localized near the boundary its response can depend strongly on whether disorder is located at the edge layer within the near-surface region of the coherence length or throughout the bulk. The manuscript does account for the finite probing depth of ARPES through exponential weighting and kz broadening which appropriately reflects the surface sensitivity of the measurement. However the spectral response is not systematically separated according to whether the disorder is edge-localized near-surface-limited or bulk-distributed. Since ARPES is a surface-sensitive probe it would be useful to compare how edge-local onsite disorder and bulk-distributed disorder differently affect the flat-band peak and how bulk disorder is projected onto the surface spectral weight. Although the manuscript compares geometric roughness with bulk Anderson disorder the relative contributions of surface disorder and bulk inhomogeneity to the suppression of the flat-band peak remain in need of further clarification.

Response to comment 7:

We agree that the distinction between edge-localized and bulk-distributed disorder should be clarified. In the manuscript, "bulk Anderson disorder" denotes an onsite random potential applied throughout the simulated slab, rather than a disorder potential restricted to the outermost edge row. This choice was made because the relevant intrinsic disorder sources in cuprates, such as dopant disorder, chemical inhomogeneity, and oxygen vacancies, are generally bulk-distributed rather than confined to the cleaved surface. At the same time, the flat-band states are localized near the boundary over a length scale set by the superconducting coherence length. Therefore, the matrix elements responsible for broadening the flat band are dominated by the component of the disorder potential that overlaps with the surface-state wave functions. Disorder far from the edge has exponentially reduced overlap with the surface component of the flat-band states and contributes much less efficiently to their broadening. In this sense, even though the random potential is implemented throughout the slab to be consistent with the known sources of disorder in cuprates, the part relevant for flat-band suppression is effectively the near-surface projection of the bulk disorder.

Revision to the manuscript

Broadening of the surface states and gap frustration by pair breaking:

its impact on the surface states. Accordingly, consistent with the deliberately minimal character of the present model, we approximate the effects of such intrinsic bulk inhomogeneity by an Anderson-type random onsite mass term applied throughout the simulated slab. This choice reflects the fact that the relevant disorder sources in cuprates, such as dopant disorder and oxygen vacancies, are not confined to the outermost surface layer. Nevertheless, the broadening of the flat-band surface state is governed mainly by the component of this disorder that overlaps with the surface-state wave functions, which are localized near the boundary over a length scale set by the superconducting coherence length. Importantly,

New Supplementary section: Different Disorder Strength and Realizations

Same Figure shown in the reply to comment 5. The bottom right Figure shows the result for disorder localized at the edge.

N. Different disorder strengths and realizations

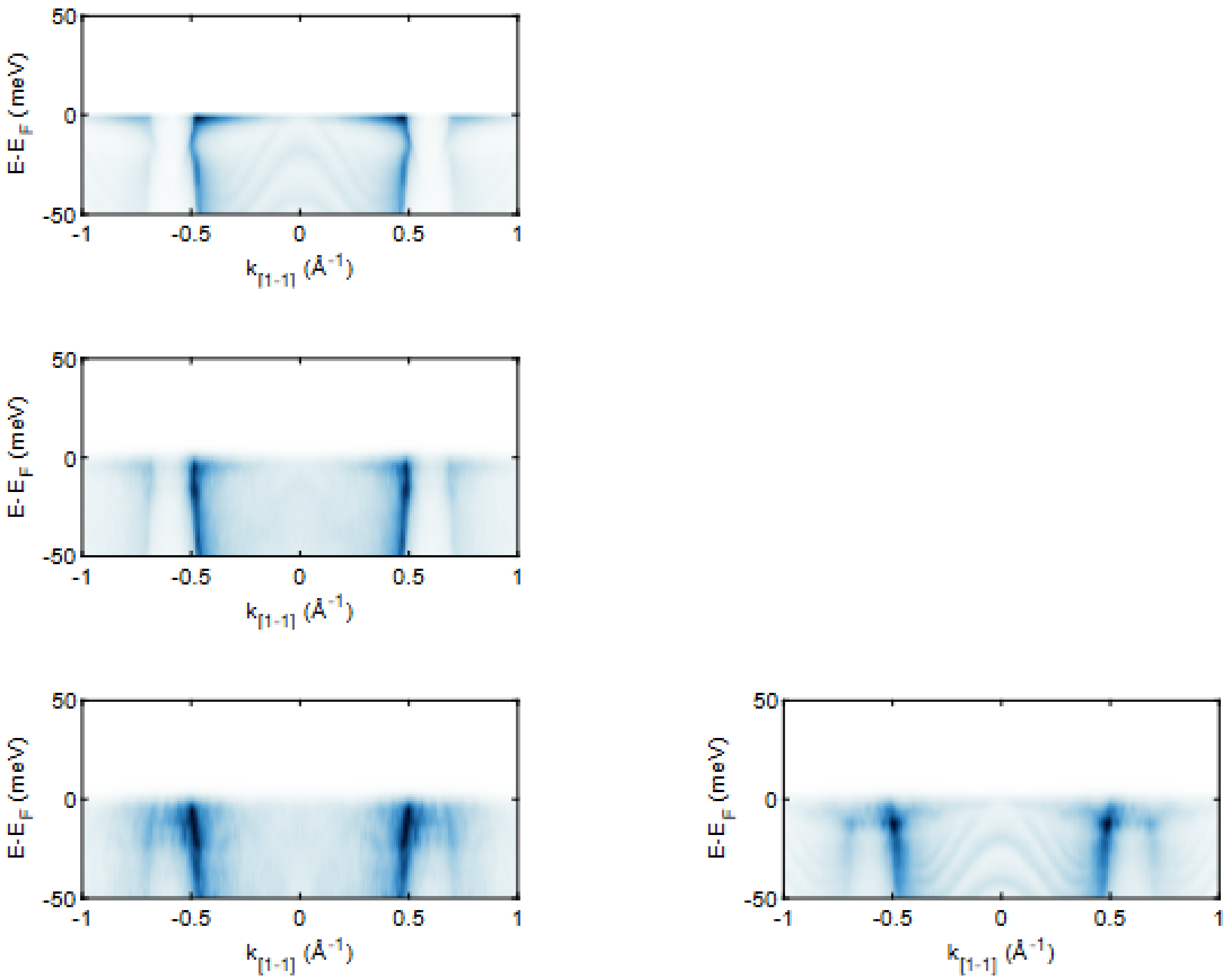

FIG. 17. The same spectral function shown in the main text, now evaluated for different disorder strengths. From top to bottom, the first three panels correspond to disorder strengths of 20 meV, 50 meV, and 100 meV. The bottom-right panel also corresponds to 100 meV disorder, but with the disorder potential applied only to the 6 atomic layers closest to the edge.

Comment 8:

6. The numerical implementation of the dx2−y2 pairing symmetry should be described more clearly. The manuscript states that a nearest-neighbor pairing field is determined self-consistently but the way in which the dx2−y2 symmetry is implemented in the actual calculation is not described in sufficient detail. For example it is unclear whether the relative sign between the x-bond and y-bond pairing amplitudes is imposed only through the initial condition whether the d-wave symmetry is explicitly constrained during the self-consistency loop or how freely the bond-resolved pairing field is allowed to vary in the presence of disorder and boundaries. Since the central analysis relies on surface order-parameter suppression and the resulting changes in

the flat-band spectral function it is important to clarify how the symmetry structure of the pairing field is maintained and interpreted during the calculation. Providing this information would improve the reproducibility and physical transparency of the self-consistent BdG simulations.

Response to comment 8:

We agree that the numerical implementation of the pairing symmetry should be described more explicitly. We have revised the Methods section accordingly.

Revision to the manuscript

Methods:

In the numerical implementation, the pairing field is represented explicitly on nearest-neighbor bonds and bonds connected to removed boundary sites are excluded from the self-consistency loop. The initial guess is chosen to have a dominant $d_{x^2-y^2}$ sign structure, with opposite signs on the two inequivalent nearest-neighbor bond directions, together with a small imaginary extended-s-wave seed, similarly to what was done in other works [26]. This seed is used only to avoid artificially excluding a possible subdominant time-reversal-symmetry-breaking component. During the self-consistency loop, each complex nearest-neighbor bond amplitude is updated independently. Thus, the magnitude and phase of the bond-resolved pairing field are allowed to vary spatially near the boundary, near rough edge segments, and in the presence of onsite disorder. We do not impose a spatially uniform d-wave amplitude, nor do we project the solution onto a fixed d-wave or $d+is$-wave form during the iteration. The only projection applied after each update is the Hermitian consistency projection of the pairing, $\Delta \to (\Delta + \Delta^\dagger)/2$, which ensures that the matrix used in the Hamiltonian remains Hermitian and allows for a faster diagonalization.

Comment 9:

In summary this manuscript presents a technically challenging and meaningful attempt to measure the cuprate side surface using momentum-resolved ARPES. The FIB-based preparation of an otherwise inaccessible crystallographic surface and the direct measurement of its electronic structure are clear strengths of the work. The self-consistent BdG calculations also provide an important framework for examining how surface roughness and disorder affect flat-band visibility. However the central interpretation that the absence of the flat-band peak can be explained by bulk-disorder-induced broadening is still not sufficiently established by the present calculations and discussion. In particular the robustness of the disorder-based scenario the representativeness of the chosen disorder model the role of competing surface-order scenarios and the details of the self-consistent BdG implementation require further clarification. Therefore while I find the experimental approach valuable and potentially important I do not think the manuscript is ready for publication in its present form. I would recommend publication only after major revision addressing the issues raised above.

Response to comment 9:

We thank the reviewer for the careful assessment and for recognizing the technical significance of the side-surface ARPES measurements and the self-consistent BdG framework. In the revised manuscript, we have addressed the points raised above by clarifying the scope and limitations of the disorder-based interpretation, the representativeness of the onsite disorder model, the possible role of competing surface orders, and the implementation of the self-consistent pairing field. These revisions make clear that we

present disorder-induced broadening as a physically plausible mechanism consistent with the data, rather than as a unique microscopic reconstruction of the LSCO disorder landscape.

Response to Reviewers

Reviewer #1

I have carefully read the revised manuscript and the authors' responses to my previous comments. The authors have made substantial efforts to improve the manuscript and have satisfactorily addressed the concerns raised in my previous report.
In particular, the authors have provided a more quantitative analysis of the surface carrier concentration based on the experimentally determined Fermi surface, added further discussion and experimental evidence regarding surface cleanliness and possible aging effects, and clarified the influence of kz broadening on the observed spectra and its treatment in the simulations. I find the authors' responses to these points convincing and the corresponding revisions appropriate.
Overall, I believe that the manuscript has been significantly improved and that my previous concerns have been adequately addressed. I therefore recommend publication in its present form.

Response:

We thank Reviewer #1 for the careful re-evaluation of our revised manuscript and for the positive assessment of the changes made in response to the previous report. We appreciate the reviewer's recognition of the additional quantitative analysis of the surface carrier concentration, the expanded discussion and evidence concerning surface cleanliness and aging, and the clarification of the role of kz broadening in the experiment and simulations. We are pleased that these revisions have addressed the reviewer's concerns and thank the reviewer for recommending publication in the present form.

Reviewer #2

The authors have responded carefully and constructively to my previous comments. In particular, the revised manuscript now includes a disorder-strength dependence, additional disorder realizations, a statistical analysis of the AFM-derived roughness, a comparison with near-surface-localized disorder, a clearer description of the self-consistent BdG implementation, and a more balanced discussion of alternative surface-instability scenarios. These additions substantially improve the robustness and transparency of the analysis. Given the significant experimental advance represented by the first momentum-resolved ARPES study of a cuprate side surface, I am satisfied that the manuscript is now suitable for publication in Nature Communications.
I retain a modest concern regarding the strength of the causal language used for the disorder interpretation. The calculations convincingly demonstrate that short-range Anderson-type disorder provides a sufficient and physically plausible mechanism for suppressing the flat-band peak, but they do not uniquely establish that disorder is the key microscopic factor in the measured LSCO surface. This limitation is appropriately acknowledged in the rebuttal and Discussion, although the final sentence of the Abstract remains somewhat stronger than the evidence warrants. I encourage the authors to consider a modest softening of this wording, but I do not regard this issue as an obstacle to publication.

Response:

We thank Reviewer #2 for the careful assessment of the revised manuscript and for the positive evaluation of the additional analyses. We agree with the reviewer that our calculations establish

short-range Anderson-type disorder as a sufficient and physically plausible mechanism for broadening the flat-band states beyond detectability, but do not uniquely identify disorder as the microscopic origin of the suppression observed experimentally.

In response to this comment, we have softened the causal language in the final sentence of the Abstract so that it more accurately reflects our results. The revised sentence now reads:

"Our results provide the first momentum-resolved view of the electronic structure on a cuprate side surface and identify disorder as a plausible mechanism obscuring the spectroscopic signatures of the predicted flat-band states."
